# Meta-Analysis of Gene Expression in Bulk-Processed Post-Mortem Spinal Cord from ALS Patients and Normal Controls

**DOI:** 10.3390/neurosci6030065

**Published:** 2025-07-16

**Authors:** William R. Swindell

**Affiliations:** Division of Hospital Medicine, Department of Internal Medicine, University of Texas Southwestern Medical Center, Dallas, TX 75390-9175, USA; william.swindell@utsouthwestern.edu

**Keywords:** microglia, motor neuron disease, neuromuscular, oligodendrocyte, RNA-seq, spatial transcriptomics

## Abstract

Amyotrophic lateral sclerosis (ALS) is characterized by upper and lower motor neuron failure and poor prognosis. This study performed a meta-analysis of gene expression datasets that compared bulk-processed post-mortem spinal cord from ALS and control (CTL) patients. The analysis included 569 samples (454 ALS, 115 CTL) from 348 individuals (262 ALS, 86 CTL). Patterns of differential expression bias, related to mRNA abundance, gene length and GC content, were discernable from individual studies but attenuated by meta-analysis. A total of 213 differentially expressed genes (DEGs) were identified (144 ALS-increased, 69 ALS-decreased). ALS-increased DEGs were most highly expressed by microglia and associated with MHC class II, immune response and leukocyte activation. ALS-decreased DEGs were abundantly expressed by mature oligodendrocytes (e.g., the MOL5 phenotype) and associated with myelin production, plasma membrane and sterol metabolism. Comparison to spatial transcriptomics data showed that DEGs were prominently expressed in white matter, with increased DEG expression strongest in the ventral/lateral white matter. These results highlight white matter as the spinal cord region most strongly associated with the shifts in mRNA abundance observed in bulk-processed tissues. These shifts can be explained by attrition of mature oligodendrocytes and an ALS-emergent microglia phenotype that is partly shared among neurodegenerative conditions.

## 1. Introduction

Amyotrophic lateral sclerosis (ALS) is a fatal neurodegenerative disease caused by death of upper and lower motor neurons due to an interplay of environmental and genetic factors [1]. Upper and lower motor neuron involvement is a distinctive feature of the disease, although those with more severe upper motor neuron impairment may progress more quickly towards disability [2]. Pathologic environmental exposures include nanosize polystyrene plastics [3] and industrial nanoparticles that have, unfortunately, been identified in tissues from children and young adults [4]. These and other predisposing factors promote cytoplasmic TDP-43 mislocalization [1], disrupt mitochondrial function and morphology [5,6], dysregulate RNA metabolism [1], and promote elevated levels of amyloid precursor protein [7]. ALS is treatable but there is no cure. Approved treatments include riluzole and edaravone, along with Nuedexta (dextromethorphan/quinidine) for pseudobulbar affect [1]. Innovative but so far unproven treatments under development include nanozymes that mimic SOD activity [8] or graphene quantum dots able to penetrate nuclear membranes and inhibit TDP-43 aggregation [9]. Other ideas include low-intensity pulsed ultrasound [10], probiotics and bacterial metabolites [11], fecal microbiota transplant [12], and nanocarrier constructs to deliver therapeutic proteins across the blood–brain barrier [13]. All of this work has been supported by an understanding of basic ALS biology and candidate disease mechanisms [14].

Transcriptomic studies of post-mortem spinal cord have provided insights into molecular-level features of lower motor neuron dysfunction in ALS [15,16]. Prior studies have been limited by sample size and/or limitations associated with bulk tissue analysis, wherein it is difficult to localize gene expression shifts with respect to cell type or anatomical location [17,18,19,20,21,22]. These challenges have been addressed, in part, by laser capture microdissection (LCM) to enrich samples for a target cell population [23,24,25,26,27,28,29]. More recent studies have utilized single-cell or single-nucleus RNA-seq to localize ALS-related gene expression shifts in post-mortem brain tissue although not yet in spinal cord [30,31,32]. Due to cost and technical factors, the LCM studies have been performed with limited sample size (≤20 patients) [33]. Meta-analysis can be useful in this setting to aggregate results across studies and identify the most robust patterns [34,35,36]. A recent meta-analysis, for example, identified 500 genes with robustly altered expression in LCM-dissected motor neurons from ALS patients (222 ALS-increased, 278 ALS-decreased) [33]. Nonetheless, aggregate sample size in this meta-analysis was limited to 89 individuals (52 ALS, 37 controls), which may be insufficient for a profoundly heterogeneous disease such as ALS [37,38,39].

Bulk tissue analysis provides an imperfect but scalable alternative to LCM or single-cell technologies [40]. A recent study, for example, used samples from the New York Genome Center ALS Consortium to perform bulk RNA-seq on 380 spinal cord sections from 203 individuals (154 ALS, 49 controls) [17]. An initial analysis of these data compared ALS to control individuals and identified 7349, 256 and 4694 genes differentially expressed (FDR < 0.05) with respect to the cervical, thoracic and lumbar spinal cord regions, respectively [17]. These totals include both protein-coding and non-coding genes with regional differences in the number of differentially expressed genes (DEGs) likely explained by sample size (*n* = 174, cervical; *n* = 52, thoracic; *n* = 154 lumbar). These data provide a rich community resource although multiple analyses will be needed to maximize its utility. The initial analysis, for example, tested for differential expression by applying fixed effect linear models using data from each spinal cord region separately [41], but an alternative strategy using mixed models could incorporate non-independent samples from the same patient to identify a consolidated set of DEGs across all spinal cord regions [42]. There is also a need to integrate and reconcile results with smaller scale gene expression studies [18,19,20,22], the catalogue of cell types found in lumbar spinal cord from normal individuals as recently delineated by single-nucleus RNA-sequencing [43], and the anatomic mapping of transcripts inferred from spatial transcriptomic studies of cord sections from both normal individuals [43] and ALS patients [44].

The current study used meta-analysis to identify and characterize genes with robustly altered expression in bulk-processed post-mortem spinal cord sections from ALS and control individuals [17,18,19,20,21,22]. Most samples were obtained from the New York Genome Center ALS Consortium (GSE137810) [17], which are here re-analyzed using a platform-specific approach with mixed effect models to identify common patterns across spinal cord regions (cervical, thoracic, lumbar). Differential expression effect size estimates are then integrated with those from prior datasets using a random effect meta-analysis framework. The resulting DEGs, representing core features of transcriptional dysregulation in the post-mortem ALS spinal cord, are then further tested for overlap with several gene sets, including those dysregulated in LCM-dissected ALS lower motor neurons [33], located within or near disease-linked genomic risk loci [45], expressed by spinal cord cell types [43], or expressed within specific spinal cord regions [43,44].

## 2. Materials and Methods

### 2.1. Study Selection

This meta-analysis included 6 studies that performed bulk tissue expression profiling of post-mortem whole spinal cord from ALS patients and control (CTL) samples [17,18,19,20,21,22]. Studies were identified through a search of Gene Expression Omnibus, Sequence Read Archive and ArrayExpress databases with inclusion of all datasets publicly released prior to 1 January 2025. Two studies meeting these inclusion criteria could not be incorporated because data were no longer available and had not been submitted to a public database [46,47]. Two studies that evaluated expression within the spinal cord anterior horn were excluded [48,49]. One study that evaluated expression in gray matter only was excluded [50]. Experiments that have used LCM to isolate cellular subpopulations were excluded [23,24,25,26,27,28,29]. One study was excluded because RNA extraction targeted microRNA rather than total cellular RNA [51].

### 2.2. GSE137810 Sample Filtering

Raw New York Genome Center (NYGC) data was obtained from Gene Expression Omnibus (GEO) under the series accession GSE137810 [17]. Samples had been obtained from 8 clinical sites, including 5 associated with the Target ALS project (Barrow Neurological Institute, Columbia University Medical Center, Georgetown University, Johns Hopkins University, University of California San Diego) and 3 associated with the ALS consortium (Academic Medical Center, University College London, Mount Sinai). Samples had been generated using either the Illumina NovaSeq 6000 (GPL24676) or Illumina HiSeq 2500 platform (GPL16791) sequencing platform. Most clinical sites had groups of samples sequenced on either platform with samples processed in multiple batches.

An initial set of 579 spinal cord samples was screened for inclusion (cervical, thoracic and lumbar), including 361 samples generated from the Illumina NovaSeq platform and 218 samples from the Illumina HiSeq platform. Samples with RNA Integrity Number (RIN) below 5 were excluded, yielding 519 samples, of which 441 were annotated as having neurologic disease and 78 were annotated controls (1 sample had unknown phenotype). Only 406 neurologic disease samples annotated as “Classical/Typical ALS” were included in the analysis. Of the 484 total samples (406 ALS, 78 CTL), there were three sample pairings representing cases in which two samples had been generated from the same spinal cord region in the same patient (SRR12167725|SRR12167978, SRR12165719|SRR12167910, SRR12167462|SRR12167687). In these cases, the sample with highest RIN was chosen, or highest library size if the RIN was the same (retained: SRR12167725, SRR12167910, SRR12167687; excluded: SRR12167978, SRR12165719, SRR12167462). Following these filtering steps, there remained 481 samples from 248 subjects evaluated in further analyses (403 ALS samples from 198 subjects, 78 CTL samples from 50 subjects).

### 2.3. GSE137810 Read Mapping

Raw fastq files with paired end reads were downloaded from the Sequence Read Archive [52]. The median read count per sample was 41.3 million (Figure S1A). FastQC (version 0.12.1) was used to perform quality control analysis of raw sequence files [53]. Trim Galore! (version 0.6.10) [54] was used for removal of illumina adaptors and trimming of reads based upon a Phred quality score threshold of 20. Quality-trimmed reads shorter than 50 bp were removed. The BBTools program bbduk.sh (version 39.08) was used to filter out reads mapping to rRNA sequences [55]. Following these pre-processing steps, the median read count per sample was 40.0 million (Figure S1B). Quality-trimmed and filtered reads were aligned to the GRCh38/hg38 genome sequence using STAR (version 2.7.10a) [56]. Binary alignment map (BAM) files generated by STAR were analyzed using samtools (version 1.13) [57]. The distribution of reads over different gene regions (e.g., exons, introns, intergenic) was evaluated using the read_distribution.py function from RSeQC (version 5.0.2) [58]. The program prepDE.py3, distributed within the stringtie software (version 2.2.1) [59], was used to calculate the number of reads mapping to each GRCh38/hg38 feature. A median of 95.4% of reads were mapped (Figure S1C) with 92.0% of reads mapped uniquely (Figure S1D). Of mapped reads, a median of 98.4% mapped to intragenic regions (Figure S1E) and 67.2% mapped to exons (Figure S1F). The median percentage of reads mapping to ribosomal genes was only 1.5% (Figure S1G). The median percentage of protein-coding genes with detectable expression was 74.6% (Figure S1H), where a gene was considered to have detectable expression in a given sample if at least 1 mapped read had been assigned with FPKM estimate ≥ 0.30 [33,60,61].

### 2.4. GSE137810 Covariates

The 481 samples were plotted with respect to the first two principal component (PC) axes to visualize sources of variation associated with gene expression (Figure S2A–J). ALS and CTL samples overlapped within two-dimensional PC space although a linear discriminant function could distinguish these groups with a balanced accuracy of 80.4% (Figure S2A). There were strong differences between ALS consortium and Target ALS samples (Figure S2B,D), and likewise, differences were seen between samples sequenced on the NovaSeq and HiSeq platforms (Figure S2C,D). These differences were related to and difficult to separate from variation in clinical site (Figure S2E) and processing batch (Figure S2F). Sample variation was also noted with respect to RNA quality, with lower RIN values seen among the Target ALS project samples sequenced on the HiSeq platform (Figure S2G). Other factors such as spinal cord region, sex and patient age were comparatively less important as factors contributing to gene expression variation (Figure S2H–J). These trends were supported by random forest variable importance scores [62], which showed that sample cluster assignment was best predicted by batch, clinical site, project (ALS consortium vs. Target ALS), sample RIN and sequencing platform, with age, sex and spinal cord region having less importance as predictor variables (Figure S2K). The same conclusion was supported by analyzing each variable separately using likelihood ratio tests to determine which most strongly decreased model deviance when added to linear models (Figure S2L). From these analyses it was clear that multivariate models would be needed to identify genes differentially expressed between ALS and CTL samples. Differential expression testing was performed separately by sequencing platform to limit model complexity and multicollinearity, as well as to remove sequencing platform as a potential confounder variable.

### 2.5. GSE137810-NovaSeq Differential Expression Analysis

The mapping protocol generated counts for 19,913 protein-coding genes and 304 samples generated using the NovaSeq (GPL24676) platform (253 ALS samples from 137 subjects, 51 CTL samples from 35 subjects). Cluster analysis did not reveal any of the 304 samples to be a strong outlier (Figure S3A). Of 19,913 genes, differential expression testing was performed for 16,044 with detectable expression in at least 15% of the ALS samples (≥38 of 253) or 15% of the CTL samples (≥8 of 51). Raw read counts were normalized by applying a variance stabilizing transformation based upon the dispersion-mean relationship within a subset of 2000 genes chosen deterministically to span the full range of mean normalized counts (R package: DESeq2, function: vst) [63].

Differential expression testing was performed using linear mixed-effect models with restricted maximum likelihood (REML) parameter estimation (R package: lmer, function: lme4) [42]. Expression of gene *i* in sample *j* (*Y*_ij_) was modeled as a function of phenotype (ALS vs. CTL), subject (treated as a random effect), batch, RIN, sex and age.

*Y*_ij_ = *β*_0_ + *β*_1_Phenotype_ij_ + β_2_Subject_ij_ + *β*_3_Batch_ij_ + *β*_4_RIN_ij_ + *β*_5_Sex_ij_ + *β*_6_Age_ij_ (1)

This model generated a non-singular fit for 99.6% of genes (15,983 of 16,044). For the remaining 61 genes with singular fits, it was possible to generate a non-singular mixed-effect model by dropping age (4 genes) or both age and sex (1 gene). Coefficients associated with phenotype were thus generated using a mixed-effect model for nearly all genes meeting criteria for detectable expression (15,988 of 16,044). For the remaining 56 genes, ALS versus CTL effect sizes were estimated using a simplified fixed effect model (R package: stats, function: lm).

Y*_ij_ = *β*_0_ + *β*_1_Phenotype_ij_ + *β*_2_Sex_ij_ + *β*_3_Age_ij_ (2)

In Equation (2), Y* represents gene expression adjusted for batch and RIN and then averaged across subject. Specifically, Y* represents residuals obtained from a linear model with only batch and RIN as predictors (R package: stats; function: lm). The residuals Y* were then averaged across samples from the same individual, such that Y* could be modeled using a fixed effect linear model (Equation (2)) without needing to include subject as a random effect (R package: stats, function: lm).

### 2.6. GSE137810-HiSeq Differential Expression Analysis

A subset of 177 samples from the GSE137810 dataset had been generated using the HiSeq (GPL16791) platform (150 ALS samples from 75 subjects, 27 CTL samples from 17 subjects). Cluster analysis of the 177 samples did not reveal a strong outlier (Figure S3B). There were 15,936 protein-coding genes with detectable expression (as defined above) in at least 15% of the ALS samples (≥23 of 150) or 15% of the CTL samples (≥5 of 27). The total number of mapped reads was normalized as above by applying a variance stabilizing transformation (R package: DESeq2, function: vst) [63]. Differential expression was then evaluated as above (Equation (1)) using linear mixed effect models (R package: lmer, function: lme4) [42]. This generated non-singular model fits for 15,857 of 15,936 genes (99.5%). For the remaining 79 genes wherein the full model (Equation (1)) yielded a singular fit, it was possible to generate a non-singular fit by dropping age (4 genes) or both age and sex (6 genes) from the model, leaving 69 genes for which the mixed-model approach did not appear suitable. For these 69 genes, fixed effect linear models were fit based upon gene expression adjusted for batch and RIN and then averaged across samples from the same individual (see Equation (2)).

### 2.7. GSE255683 Analysis

The GSE255683 dataset includes lumbar spinal cord samples from 10 ALS and 10 CTL subjects. All 20 samples were from unique individuals [20]. Processed data are available under GEO accession GSE255683. Both raw and processed data have been submitted to the European Genome-phenome Archive (EGA) (study no. EGAS50000000575, datasets EGAD50000000820 and EGAD50000000833). Raw fastq files were quality-assessed, filtered and mapped to the GRCh38/hg38 genome sequence using the protocol described above. There was an average of 54.7 million reads per sample prior to quality-filtering (Figure S4A) and 48.1 million per sample after filtering (Figure S4B). An average of 96.6% of reads mapped to the GRCh38/hg38 genome (Figure S4C) and 93.6% of these mapped uniquely (Figure S4D). The average number of intragenic reads was 95.4% (Figure S4E) with 93.0% of reads assigned to exons (Figure S4F). The average number of reads assigned to ribosomal genes was 4.6% among samples (Figure S4G). An average of 68.4% of protein-coding genes had detectable expression (i.e., FPKM ≥ 0.30 with at least one read mapped to the gene; see Figure S4H) [33,60,61]. Raw gene counts were obtained for 19,913 protein-coding genes in 20 samples. Cluster analysis did not demonstrate any sample outliers (Figure S3C). Of the 19,913 genes, differential expression analyses included 15,204 having detectable expression in at least 15% of ALS or 15% of CTL samples. Since all samples had been generated from unique subjects, expression was modeled using a fixed effect linear model as specified in Equation (3) (R package: stats, function: lm).

*Y*_ij_ = *β*_0_ + *β*_1_Phenotype_ij_ + *β*_2_Sex_ij_ + *β*_3_Age_ij_ (3)

### 2.8. SRP064478 Analysis

The SRP064478 dataset includes 15 samples from unique patients (8 CTL, 7 ALS) with an average of 68.6 million reads per sample (Figure S5A) [21]. Reads were quality-filtered as described above, yielding an average of 65.7 million reads per sample (Figure S5B). Filtered reads were mapped to the GRCh38/hg38 genome using STAR [56]. An average of 96.6% of reads were mapped (Figure S5C) with 90.6% of reads having been mapped uniquely (Figure S5D). An average of 70.0% of reads were assigned to intragenic regions (Figure S5E) with 47.1% assigned to exons (Figure S5F). Prior to read mapping, the BBTools program bbduk.sh (version 39.08) had been used to filter out some reads mapping to rRNA sequences [55]. Despite this, a relatively high percentage of the filtered reads (40.4%) were assigned to ribosomal genes on average (Figure S5G).

The mapping protocol generated counts for 19,913 protein-coding genes. One sample, CTL-54M-2 (SRR2558720), was a questionable outlier based on cluster analysis (Figure S3D). This sample was retained, however, since given the small sample size (*n* = 15), there was limited power to detect true outliers. Moreover, the sample was otherwise unremarkable with respect to mapping statistics (Figure S5). An average of 69.3% of these genes had detectable expression among the 15 samples based on above-stated criteria (Figure S5H). Differential expression testing was performed for 14,791 genes having detectable expression in at least 15% of ALS (≥2 of 7) or 15% of CTL samples (≥2 of 8). Gene counts were normalized by applying a variance-stabilizing transformation as above (R package: DESeq2, function: vst) [63]. Differential expression testing was then performed using fixed effect linear models with sex and age as covariates (see Equation (3)).

### 2.9. GSE26927 Analysis

The GSE26927 dataset was generated from BrainNet Europe network samples [18,19]. The current analysis focused on 20 samples from cervical spinal cord (10 ALS samples from 9 subjects, 10 CTL samples from 7 subjects). Expression profiling was carried out using the Illumina humanRef-8 v2.0 expression beadchip (GPL6255). This platform includes 20,589 probes corresponding to 16,718 unique human genes. Non-normalized probe signals were background-corrected using a normal/exponential convolution model (R library: limma; function: backgroundCorrect) [64]. The distribution of raw and background-corrected signal intensities was dissimilar among samples (Figure S6A,B). Quantile normalization was thus performed to equalize the empirical distribution of intensities to ensure comparability of samples (Figure S6C; R library: limma, function: normalizeBetweenArrays) [65]. Final normalized signal intensities were log_2_-transformed prior to differential expression analysis. If multiple probes were associated with the same human gene [66], the single probe with highest average normalized expression across all samples was selected for further analysis. This generated a raw data matrix with 16,718 probes uniquely assigned to the same number of human genes, of which 16,258 encoded a known protein.

The number of protein-coding genes having detectable expression varied from 11,202 to 12,778 among samples (Figure S6D). Genes with a Rosetta error model detection *p*-value less than 0.05 were considered to have detectable expression [67]. Based on this criterion, differential expression testing was performed for 10,960 protein-coding genes having detectable expression in at least 15% of ALS (≥2 of 10) or at least 15% of CTL samples (≥2 of 10). Linear mixed effect models specified as below, with subject as a random effect, were used to test for differential expression (R package: lmer, function: lme4) [42].

*Y*_ij_ = *β*_0_ + *β*_1_Phenotype_ij_ + *β*_2_Subject_ij_ + *β*_3_RIN_ij_ + *β*_4_Sex_ij_ + *β*_5_Age_ij_ (4)

The above model led to non-singular model estimates for 91.0% of genes (9974 of 10,960). For 291 of the remaining 986 genes, it was possible to generate a non-singular fit by dropping RIN as a predictor. For the remaining 695 genes an alternative approach was followed, in accordance with Equation (2) above, with normalized expression intensities first adjusted for the sample-specific variable (RIN), averaging of adjusted expression intensities across samples from the same subject, with a final model having only phenotype, sex and age as fixed effects (R package: stats, function: lm).

### 2.10. E-MTAB-8635 Analysis

The E-MTAB-8635 dataset includes 40 single-channel (Cy3) microarray hybridizations performed using the Agilent-014850 Whole Human Genome Microarray 4x44K [22]. Samples had been generated from post-mortem lumbar spinal cord of unique individuals (10 CTL, 30 ALS). Raw data files generated from GenePix Pro 6.0 software were downloaded from ArrayExpress [68]. Raw files included median Cy3 foreground and background signals for 45,215 probes. There were 4 samples with foreground and background signal intensities higher than those seen on other arrays (ALS-38M-20, ALS-54M-21, ALS-61F-24, ALS-67F-25; see Figure S7A,B). Foreground intensities remained high for these samples following background correction using a normal/exponential convolution model (library: limma, function: backgroundCorrect) (Figure S7C) [64]. Probe intensity distributions were equalized among microarray samples using quantile normalization (library: limma, function: normalizeBetweenArrays) [65].

Inspection of microarray pseudoimages revealed mild-to-severe spatial artifacts for several hybridizations (Figure S8). To correct for this, a loess model was fit to the surface of each array, using probe intensities as the response variable and two-dimensional probe coordinates as predictors [69] (R package: stats; function: loess). Residuals from this model had similar distributions among samples (Figure S7E) and were used for further analyses. Re-inspection of microarray pseudoimages, based on background-corrected quantile-normalized spatially corrected probe intensities, revealed improvement or resolution of spatial artifact for most samples, although prominent artifacts remained for two samples (ALS-40F-1 and ALS-43M-22) (Figure S9). Subsequent cluster analysis identified 5 samples as outliers, including CTL-60M-1 and the 4 other samples already flagged as problematic due to an aberrant signal intensity distribution (Figure S3F).

Differential expression testing was performed after removal of the 7 problematic samples mentioned above (ALS-38M-20, ALS-54M-21, ALS-61F-24, ALS-67F-25, ALS-40F-1, ALS-43M-22, CTL-60M-1). Following this filtering, there remained 33 samples (9 CTL, 34 ALS). Of 45,215 probes, some had been assigned to the same gene symbol [66], in which case the single probe with highest average expression among samples was retained. This yielded 18,960 probes each corresponding to a unique human gene, of which 17,322 were protein-coding. A probe signal was considered to be detectable if its expression exceeded the 95th percentile of normalized signals among 153 negative control probes. Differential expression testing was performed for 16,903 genes with detectable expression in at least 15% of ALS (≥6 of 34) or 15% of CTL (≥2 of 9) samples. This was performed using fixed effect linear models with a phenotype term plus sex and age as covariates (see Equation (3)) (R package: stats, function: lm).

### 2.11. Meta-Analysis

The number of protein-coding genes included in differential expression analyses varied from 13,634 to 16,921 among the six studies (Table 1). Meta-analysis was performed for 15,852 genes included in at least 3 of the 6 differential expression analyses. For these genes, effect size in each study was calculated based upon Hedge’s g estimator of the standardized mean difference (SMD) [70,71]. This was calculated from linear model coefficient estimates as defined above, which for each study included a fixed effect indicator variable (phenotype) defined to have a value of 1 for ALS patients (otherwise 0 for CTL patients; see Equations (1)–(3) above). The unstandardized regression coefficient associated with this variable was used to calculate adjusted SMD for each gene (R package: esc, function: esc_B) [72]. A random effects meta-analysis model was then used to calculate the SMD meta-estimate based on the inverse variance method (R package: meta, function: metagen) [73]. Raw *p*-values generated from the meta-analysis model were adjusted using the Benjamini–Hochberg method to control the false-discovery rate [74].

### 2.12. Single-Nucleus Transcriptomics of Normal Human Spinal Cord (GSE190442)

Meta-analysis DEGs were evaluated to assess their expression pattern across cell types that had been identified from single-nucleus RNA-sequencing of post-mortem lumbar spinal cord from normal adults (GSE190442) [43]. This prior study had generated sequencing data for 7 donors (55289 nuclei) using the 10x Genomic Chromium 3′ and Illumina HiSeq 3000 platforms. Prior analysis of these data had assigned nuclei to one of 11 major cell type classes (astrocytes, endothelial, ependymal cells, lymphocytes, meninges, microglia, neurons, oligodendrocytes, oligodendrocyte precursor cells (OPCs), pericytes, Schwann cells), which had been further subdivided into 64 cellular subtypes (35 neuronal, 29 non-neuronal) [43]. The current analysis was performed using feature counts generated by mapping reads to the GRCh38 (hg38) genome (file: GSE190442_aggregated_counts_postqc.csv.gz). Raw counts were analyzed using the log_10_(CPM + 1) transformation, where CPM represents counts per million mapped reads.

### 2.13. Spatial Transcriptomics of the Normal Spinal Cord (GSE222322)

Previously reported spatial transcriptomics data from post-mortem lumbar spinal cord sections from normal individuals was analyzed [43]. Data was available for 20 sections from 4 donors and had been generated using the 10x Genomics Visium and Illumina HiSeq 3000 platforms. Analyses were performed using Spaceranger output files (H5 file and image data) provided by Gene Expression Omnibus (GSE222322). Raw counts for each slide were normalized using a variance stabilizing transformation with regularized negative binomial regression model (R package: Seurat, function: SCTransform) [75]. The molecular count distribution, feature count distribution, and percentage of features mapping to mitochondrial genes was similar among 16 of 20 sections (Figure S10). There were 4 slides, however, which had been generated from the same donor, which had markedly lower molecular and feature counts (GSM6919905, GSM6919906, GSM6919907, GSM6919908; Figure S10A,B). For nearly all slides, molecular counts, feature counts and the percentage of mitochondrial features was highest in gray matter regions (Figures S11–S13).

### 2.14. Spatial Transcriptomics of ALS Spinal Cord

Spatial transcriptomic analysis of ALS spinal cord was performed using previously published data from 11 patients [44]. The analysis was carried out using data from 80 slides (39 cervical, 41 lumbar) with 3 to 12 slides having been generated per patient. Transcriptome data has been generated using the Illumina NextSeq 550 System sequencer. Downloaded supplemental files from the original publication included raw H&E images, feature count matrices and spot coordinates with regional annotations. Spots had been assigned to one of 11 anatomical regions (central canal, dorsal edge, dorsal horn, dorsal medial white, lateral edge, medial gray, medial lateral white, ventral edge, ventral horn, ventral lateral white, ventral medial white). Counts were associated with 64,330 spots with an average of 804 spots per slide (range: 249–985). Raw counts were normalized using a variance stabilizing transformation as above (R package: Seurat, function: SCTransform) [75]. Counts were generated from an average of 17,338 unique symbols per slide (range: 10,923–18,712). Of these, the current analysis considered 13,931 protein-coding genes expressed at sufficiently high levels to have been included in the transcriptome meta-analysis.

## 3. Results

### 3.1. Dataset Comparison

**Figure 1:**
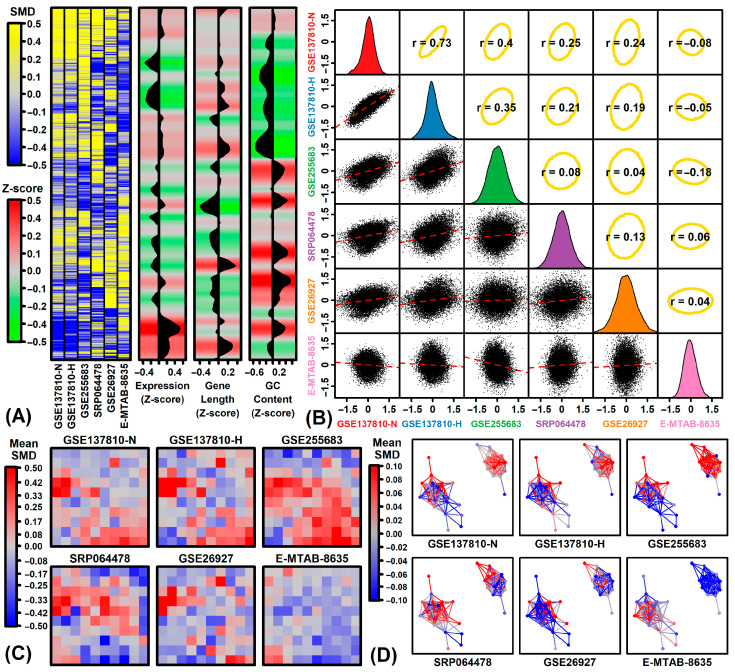
SMD estimates. (**A**) Clustered heatmap. SMD estimates from each experiment are shown in the blue–yellow heatmap. Rows (genes) have been ordered using hierarchical cluster analysis with average linkage and the Euclidean distance metric. Z-scores corresponding to gene characteristics are shown on the right (i.e., relative mRNA abundance, gene length, GC content). Black regions outline the z-score trend across genes based on a loess curve fit (horizontal axis). (**B**) SMD correlations between experiments. Diagonal: Distribution of SMD estimates from each experiment. Below-diagonal: SMD scatterplots with each point representing an individual gene (red line: least square regression fit). Above-diagonal: Ellipses outline the middle 90% of genes (based on Mahalanobis distance). Spearman rank correlation estimates are shown (center of each ellipse). (**C**) Self-organizing map (SOM). An SOM was generated by assigning genes to locations within the square space, based on the similarity of expression patterns in an independent RNA-seq dataset generated from a broad range of human tissue and cell line samples (GSE138734). The SOM was then color-coded based on the average of SMD estimates among genes assigned to each SOM sub-region for each experiment. (**D**) Module network. A network was generated using an independent tissue atlas dataset (GSE138734). Hierarchical clustering was used to assign genes to each node representing a gene expression cluster (≥100 genes). Connections are drawn between nodes having correlated centroids (*r* > 0.90). Node layout was determined based upon the Kamada-Kawai algorithm. Nodes are color-coded based upon the average SMD among genes assigned to that node (see scale). Likewise, connections are color-coded based upon the average SMD among genes assigned to both nodes (see scale).

Cluster analysis of SMD estimates among protein-coding genes showed strong agreement between GSE137810-N and GSE137810-H (Figure 1A). Many genes having ALS-decreased expression in GSE137810-N and GSE137810-H were highly expressed in spinal cord (Figure 1A). Likewise, ALS-decreased genes from E-MTAB-8635 often had low GC content (Figure 1A). The correlation between SMD estimates from GSE137810-N and GSE137810-H was strong (*r* = 0.73), although most pairwise correlations among datasets were positive (*r* > 0) (Figure 1B). SMD estimates from E-MTAB-8635 were negatively correlated with 3 of the 5 other datasets (Figure 1B). An independent gene expression tissue atlas dataset [76] was used to generate a self-organizing map (SOM) and module network (Figure 1C,D). Color-coding based upon SMD estimates then revealed a sub-network of genes down-regulated in E-MTAB-8635 but up-regulated in GSE255683 and to a lesser degree in GSE137810-N and GSE137810-H (Figure 1C,D).

ALS and CTL samples for each dataset overlapped when plotted in two-dimensional principal component (PC) space (Figure S14A–F). However, a linear discriminant function could distinguish ALS and CTL samples with a balanced accuracy that ranged from 52.7% (SRP064478) to 81.1% (GSE137810-H) (Figure S14A–F). For GSE137810-N and GSE137810-H, ALS and CTL samples differed significantly with respect to one or both of the top 2 PC axes (*p* < 0.05, two-sample two-tailed *t*-test; Figure S14G,H). For the 4 other datasets, there was a significant ALS vs. CTL sample difference with respect to at least one of the top 7 PC axes (*p* < 0.05, two-sample two-tailed t-test; Figure S14I,L).

### 3.2. Differential Expression Bias (mRNA Abundance, Gene Length, GC Content)

Differential expression testing (ALS vs. CTL) demonstrated significant findings with respect to GSE137810-N and GSE137810-H (Figure S15). For these datasets, *p*-value distributions were L-shaped with an overabundance of genes having low *p*-values (Figure S15G,H). This trend was less prominent for GSE255683 (Figure S15I), SRP064478 (Figure S15J) and GSE26927 (Figure S15K), with a uniform *p*-value distribution observed for E-MTAB-8635 (Figure S15L). Based on a stringent threshold (FDR < 0.05 with SMD > 0.80 or SMD < −0.80) it was only possible to identify DEGs with respect to GSE137810-N and GSE137810-H (Table 1). When a less stringent differential expression threshold was applied (*p* < 0.05 with SMD > 0.80 or SMD < −0.80), it was possible to identify between 716 (E-MTAB-8635) and 1811 DEGs (GSE137810-H) (Figure S16). The number of genes having ALS-increased and ALS-decreased expression was balanced for each dataset (Figure S16A–F). Genes having higher expression tended to be ALS-decreased with respect to GSE137810-N (Figure S16G), GSE255683 (Figure S16I) and SRP064478 (Figure S16J) (not observed for other datasets; see Figure S16H,K,L).

Differential expression bias was detected with respect to gene length and GC content (Figure S17). For GSE137810-N and GSE137810-H, ALS-decreased DEGs were more frequent among longer genes (Figure S17A,B), but an opposite pattern was seen with respect to GSE255683 (Figure S17C) and GSE26927 (Figure S17E), whereas no clear trend was observed for SRP064478 (Figure S17D) and E-MTAB-8635 (Figure S17F). ALS-increased and ALS-decreased DEGs were more frequent among genes having intermediate GC content for GSE137810-N and GSE137810-H (Figure S17G,H). However, for GSE255683, ALS-increased DEGs were more common among genes having low GC content (Figure S17I) and the opposite was observed for E-MTAB-8635 (Figure S17L).

### 3.3. Meta-Analysis Moderates Differential Expression Bias

Meta-analysis of SMD estimates was performed for 15,852 protein-coding genes having detectable expression in ≥3 of 6 studies. This identified 213 DEGs based on stringent criteria, including 144 ALS-increased DEGs (FDR < 0.05, SMD > 0.80) and 69 ALS-decreased DEGs (FDR < 0.05, SMD < −0.80) (Figure S18C). All ALS-increased and ALS-decreased DEGs are listed in Additional Files 1 and 2, respectively. These files also list 62 non-coding mRNAs meeting the same differential expression criteria (26 ALS-increased, 36 ALS-decreased), although the current manuscript focuses only on the protein-coding DEGs identified.

Raw meta-analysis *p*-values among protein-coding genes were L-shaped with an overabundance of genes having low *p*-values (Figure S18A,B). There was an absence of ALS-decreased DEGs among genes having weak expression, but no overall relationship between SMD estimates and mRNA abundance was appreciated (Figure S18D). Likewise, although short genes were rarely differentially expressed, there was no systematic relationship between DEG frequency and gene length (Figure S18E). There remained an increased frequency of ALS-decreased DEGs among genes with intermediate GC content (Figure S18F) although this pattern was attenuated compared to that seen for individual datasets (Figure S17G–L).

### 3.4. Genes with Expression Consistently Altered in ALS Spinal Cord

**Figure 2:**
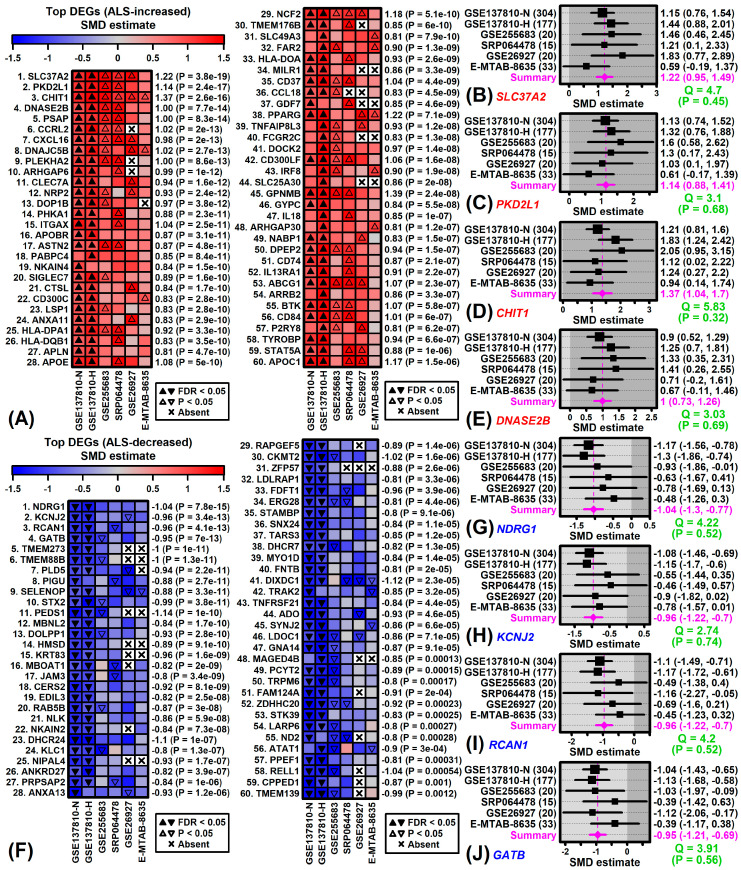
ALS-increased and ALS-decreased DEGs (meta-analysis). (**A**) Top ALS-increased DEGs (ranked by *p*-value). Heatmaps are color-coded based on experiment-specific SMD estimates. (**B**) *SLC37A2* forest plot. (**C**) *PKD2L1* forest plot. (**D**) *CHIT1* forest plot. (**E**) *DNASE2B* forest plot. (**F**) Top ALS-decreased DEGs (ranked by *p*-value). Heatmaps are color-coded based on experiment-specific SMD estimates. (**G**) *NDRG1* forest plot. (**H**) *KCNJ2* forest plot. (**I**) *RCAN1* forest plot. (**J**) *GATB* forest plot. In (**B**–**E**,**G**–**J**), SMD estimates from each experiment are shown with meta-estimate (bottom). The total number of samples from each experiment is shown (left margin parentheses) along with the SMD point estimate and confidence interval (right margin). The heterogeneity test statistic (Cochran’s Q) is shown with *p*-value (bottom-right).

Meta-analysis identified genes most consistently altered in ALS spinal cord with similar trends across the 6 studies (Figure 2A,F). Genes most strongly increased by ALS included *SLC37A2* (Figure 2B), *PKD2L1* (Figure 2C), *CHIT1* (Figure 2D) and *DNASE2B* (Figure 2E). Expression of solute carrier family 37 member 2 (*SLC37A2*) was increased in ALS patients from all studies (SMD ≥ 0.59) with a meta-SMD estimate of 1.22 (*p* = 3.8 × 10^−19^) (Figure 2B and Figure S19). *SLC37A2* expression was significantly increased in both GSE137810-N and GSE137810-H (FDR < 0.05) and also increased with *p* < 0.05 in 3 of 4 other studies (Figure 2B and Figure S19). Genes most strongly decreased by ALS included *NDRG1* (Figure 2G), *KCNJ2* (Figure 2H), *RCAN1* (Figure 2I) and *GATB* (Figure 2J). N-myc downstream regulated 1 (*NDRG1*) expression was only significantly decreased in GSE137810-N and GSE137810-H (FDR < 0.05) (Figure 2G and Figure S20). However, a similar trend was seen in each study (SMD ≤ −0.48) and the overall meta-SMD estimate was −1.04 (*p* = 7.8 × 10^−15^) (Figure 2G and Figure S20).

**Figure 3:**
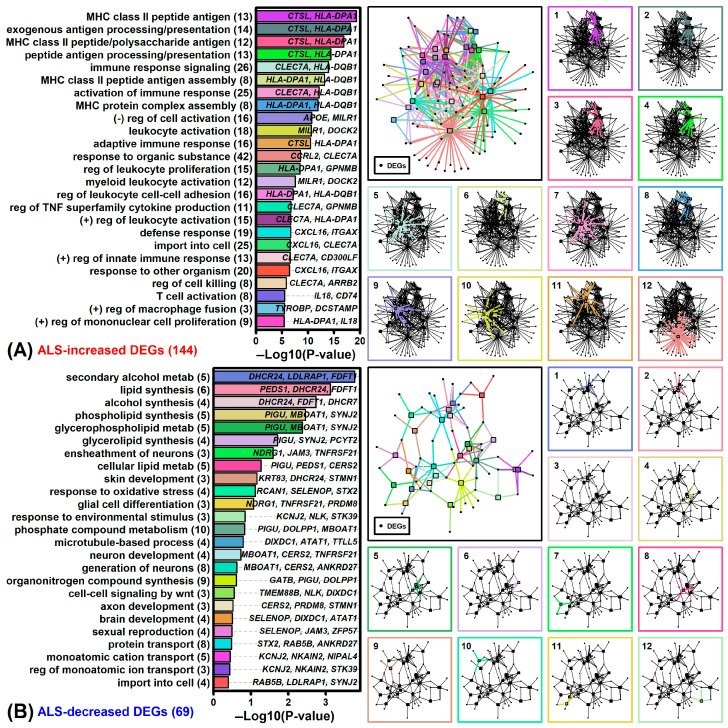
GO BP terms. (**A**) GO BP terms enriched among ALS-increased DEGs (SMD ≥ 0.80, FDR < 0.05). (**B**) GO BP terms enriched among ALS-decreased DEGs (SMD ≤ -0.80, FDR < 0.05). In (**A**) and (**B**), bar charts (left) show the level of enrichment for the 25 top-ranked GO BP terms (exemplar genes listed within each figure). The degree of enrichment (horizontal axis) is proportional to the -log_10_-transformed *p*-value (conditional hypergeometric test for enrichment). The number of DEGs associated with each GO BP term is listed in parentheses (left margin). A color-coded network of the top 25 GO BP terms is shown (right) for both (**A**) increased and (**B**) decreased DEGs. Networks were generated using the Kamada-Kawai algorithm and show connections between GO BP terms (squares) and DEGs (circles). In the larger network (top-left), connections between GO BP terms and DEGs are color-coded (see bar chart colors on left). Smaller networks show only color-coded connections between DEGs and one of the 12 top-ranked GO BP terms (rank shown in upper-left).

ALS-increased DEGs were most strongly associated with Gene Ontology (GO) biological process (BP) terms related to major histocompatibility complex class II, immune response and leukocyte activation (Figure 3A and Figure S21A). ALS-increased DEGs were also enriched for genes related to phagosome, vesicle, plasma membrane, TYROBP microglia network, CD3/TCR zeta chain phosphorylation, demyelinating disease, macrophage activation and the nonsteroidal anti-inflammatory drug lumiracoxib (*p* < 0.05, Additional File 1). ALS-increased DEGs were frequently identified as targets of lncRNAs (e.g., *lnrCXCR4*, *RMEL3*, *DINO*) and microRNAs (e.g., *hsa-miR-4537*, *hsa-miR-5739*, *miR-708*) (*p* < 0.05, Supplementary Material File S1). Several ALS-increased DEGs were known to interact with the mRNA encoding ELAV like RNA binding protein 1 (ELAVL1) (Supplementary Material File S1).

ALS-decreased DEGs were most strongly associated with GO BP terms related to lipid or alcohol metabolism/synthesis, neuron ensheathment and oxidative stress response (Figure 3B and Figure S21B). Such genes were also associated with endoplasmic reticulum membrane, iron ion binding, steroid/cholesterol biosynthesis, Down syndrome and event-related potentials (*p* < 0.05, Supplementary Material File S2). ALS-decreased DEGs were enriched as targets of lncRNAs (e.g., *CAT8*, *lincZFP161*, *FMR4*) and microRNAs (*hsa-miR-6894-3p*, *hsa-miR-3944-5p*, *miR-219-1-3p*) (*p* < 0.05, Supplementary Material File S2). ALS-decreased DEGs frequently interacted with RNAs encoding myocilin opposite strand (*MYOCOS*) and the chaperone protein calreticulin (*CALR*) (Supplementary Material File S2).

Meta-analysis DEGs were compared to those identified from the prior NYGC data analysis of Humphrey et al. [17]. Of 142 ALS-increased DEGs identified by meta-analysis, only 25 were significantly increased in each spinal cord region (FDR < 0.05) based on the prior analysis (*p* = 4.2 × 10^−33^, Fisher’s exact test, Figure S22A). Likewise, of 64 ALS-decreased DEGs identified by meta-analysis, only 8 were significantly decreased in each cord region (FDR < 0.05) (*p* = 6.4 × 10^−11^, Fisher’s exact test, Figure S22B). All genes significantly increased across cord segments (FDR < 0.05) in the prior analysis had meta-analysis SMD estimates > 0 (Figure S22C), and all genes decreased across cord segments (FDR < 0.05) had meta-analysis SMD estimates < 0 (Figure S22D), demonstrating similar effect size trends in both analyses despite differences in DEG sets identified. Genes such as *GPNMB*, *PPARG* and *OTOA* met increased DEG criteria in both analyses (Figure S22E), while *DHCR24*, *NDRG1* and *CKMT2* satisfied decreased DEG criteria in both analyses (Figure S22F).

### 3.5. Spinal Cord DEGs Overlap Significantly with Those Identified in LCM-Dissected Motor Neurons and mRNAs Associated with ALS-Dysregulated Proteins

A recent meta-analysis of 6 studies identified 222 ALS-increased DEGs (FDR < 0.10, SMD > 0.80) and 278 ALS-decreased DEGs (FDR < 0.10, SMD < −0.80) with consistent expression changes in LCM-dissected motor neurons [33]. Although only 13 of 213 DEGs from the current study were identified as DEGs in this previous work, this overlap was significant when considering only genes with detectable expression in both analyses (Figure S23A,B). Most ALS-increased DEGs from the LCM meta-analysis were also ALS-increased in the current study (Figure S23C), and conversely, the majority of ALS-decreased DEGs from the LCM meta-analysis were correspondingly decreased in the current study (Figure S23D). Genes increased in both meta-analyses included *GPNMB*, *NCF2* and *NCKAP1L* (Figure S23E), whereas genes decreased in both studies included *NLK1*, *LDOC1* and *PPEF1* (Figure S23F).

DEGs from the current study were next compared to differentially expressed proteins (DEPs) identified in post-mortem ALS spinal cord [77]. Only 13 DEGs from this study were associated with a correspondingly altered DEP from the prior study although this overlap was significant (Figures S24A and S24B). Nearly 80% of ALS-increased DEPs were associated with mRNAs having ALS-increased expression (SMD > 0; Figure S24C). Likewise, most (63%) of the ALS-decreased DEPs were associated with mRNAs having ALS-decreased expression (SMD < 0; Figure S24D). ALS-increased DEGs linked to ALS-increased proteins included *GPNMB*, *IQGAP2* and *APOE* (Figure S24E), whereas ALS-decreased DEGs associated with ALS-decreased proteins included *CKMT2*, *EDIL3* and *LDLRAP1* (Figure S24F).

RNA species within the CNS may permeate into peripheral blood via extracellular vesicle transport [78]. However, DEGs from the current study did not overlap significantly with 752 ALS-increased and 764 ALS-decreased DEGs previously identified in whole blood from ALS patients (Figure S25A,B) [79]. Most ALS-increased DEGs from blood did have elevated expression in spinal cord (SMD > 0; Figure S25C), although ALS-decreased DEGs from blood were not biased towards ALS-decreased expression in spinal cord (SMD < 0; Figure S25D). Genes having significantly increased expression in both tissues from ALS patients included *ABCG1*, *CTSS* and *CXCL16* (Figure S25E), while genes having significantly decreased expression in both tissues included *PIGU*, *SYNJ2* and *MMAB* (Figure S25F).

### 3.6. ALS DEGs and Genes near ALS Risk Loci Are Associated with Plasma Membrane and Sterol Metabolism

The NHGRI-EBI GWAS catalog was used to identify 410 genes within or near ALS risk loci [45], of which 257 were protein-coding genes with detectable expression in spinal cord (Figure S26A). The 257 genes did not have disproportionately increased or decreased expression in ALS spinal cord (*p* = 0.14, Figure S26B). Additionally, there was no significant overlap between DEGs from the current study and genes near ALS risk loci (Figure S26C–F). Genomic distance between ALS DEGs and risk loci did not differ significantly from that seen in randomly sampled sets of genes of the same size (Figure S26G–I). DEGs from the current study, however, did demonstrate functional correspondence to those genes near ALS risk loci (Figure S26J–M). For example, among 136 GO BP terms enriched among genes near risk loci (*p* < 0.05), 29 were also significantly enriched among ALS-increased DEGs (*p* < 0.05), which is a larger number than that seen among randomly sampled genes of the same size (*p* = 0.02, Figure S26J). It was possible to identify gene annotations enriched among ALS-increased DEGs, ALS-decreased DEGs and genes near risk loci (*p* < 0.05 for each), which were related to sterol metabolism, plasma membrane, maturation of protein 3a, phospho-PLA2 pathway and NTRK3 signaling (Figure S26J,K,M).

### 3.7. ALS-Increased DEGs Are Most Strongly Expressed by Microglia

Gene expression changes in bulk-processed tissue are partly reflective of changes in cellular composition that may occur with disease onset or progression [80,81]. To better understand how this may impact differential expression in ALS spinal cord, expression of DEGs was evaluated in cell populations characterized by snRNA-seq analysis of adult human post-mortem lumbar spinal cord [43].

**Figure 4:**
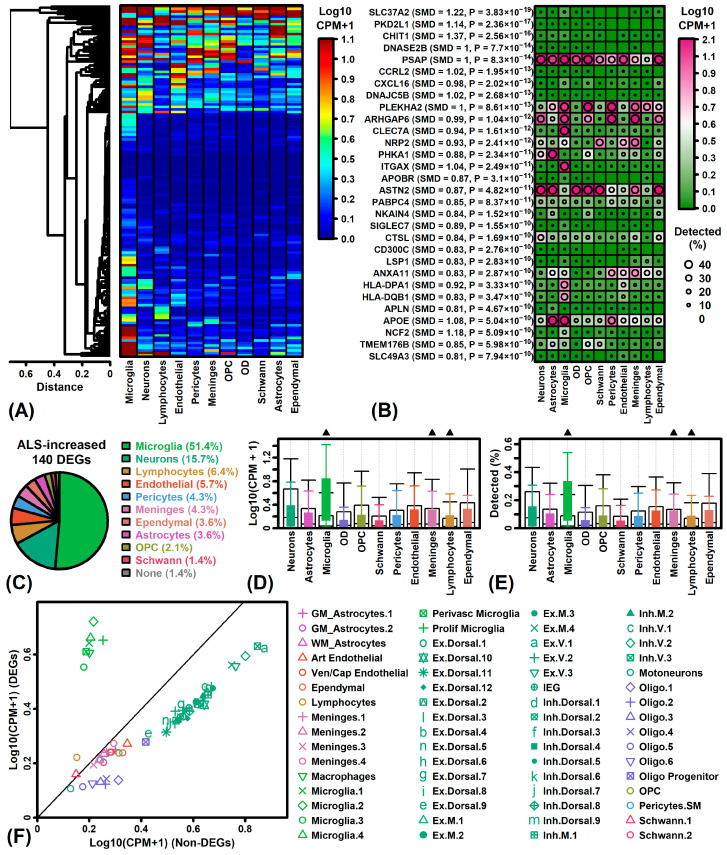
ALS-increased DEGs and their expression in human spinal cord cell types (GSE190442). (**A**) Cluster analysis. The heatmap shows average expression of ALS-increased DEGs across 11 cell type categories. DEGs have been hierarchically clustered using average linkage and the Euclidean distance. (**B**) Top-ranked ALS-increased DEGs. The heatmap shows top ALS-increased DEGs and their average expression across 11 cell type categories. The heatmap color corresponds to average expression and circles indicate the percentage of nuclei with detectable expression. (**C**) Percentage of DEGs assigned to each cell type category. ALS-increased DEGs were assigned to the cell type category for which average expression was highest. (**D**) Expression level of DEGs versus non-DEGs by cell type category. (**E**) Percentage of nuclei with detectable expression in DEGs versus non-DEGs by cell type category. In (**D**,**E**), boxes outline the middle 50% of values (whiskers: 10th to 90th percentiles). Clear boxes (background) correspond to non-DEGs whereas colored boxes correspond to DEGs. Filled triangles (top margin) denote cases in which DEG expression is significantly greater than non-DEGs (up-triangles) or significantly less than non-DEGs (down-triangles) (Wilcoxon rank sum test, FDR < 0.05). (**F**) Average expression of DEGs versus non-DEGs by cell type sup-population. A different symbol is used for each sub-population whereas all sub-populations within the same category share the same color.

ALS-increased DEGs often had strong expression in microglia, neurons and endothelial cells, although many were expressed by multiple cell types and some weakly expressed in all cell types (Figure 4A). Certain increased DEGs were much more strongly expressed in microglia compared to other cell types (e.g., *CLEC7A*, *ITGAX*, *HLA-DPA1*, *HLA-DQB1*; Figure 4B). Approximately half of ALS-increased DEGs (51.4%) had the highest expression in microglia (Figure 4C). On average, ALS-increased DEGs had significantly higher expression in microglia, meninges and lymphocytes as compared to non-DEGs with detectable expression in bulk-processed spinal cord (*p* < 0.05; Figure 4D,E). Analysis of cellular subpopulations revealed similar trends, with ALS-increased DEGs having higher average expression in microglia subpopulations, but lower average expression in most other subpopulations (Figure 4F). Consistent with these findings, ALS-increased DEGs overlapped significantly with microglia genes having increased expression in a prior snRNA-seq study of post-mortem motor cortex in ALS patients (e.g., *GPNMB*, *APOC1*, *SIGLEC8*; Figure S27) [31].

**Figure 5:**
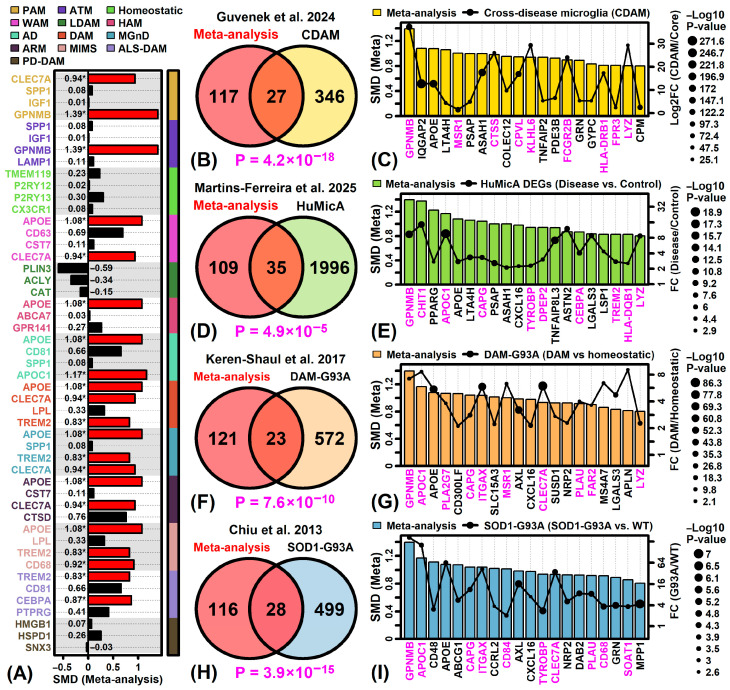
Microglia signature genes. (**A**) Microglia phenotypes and marker genes. SMD estimates are shown for marker genes associated with homeostatic microglia and 12 other phenotypes (PAM: proliferative associated microglia; ATM: axon tract associated microglia; WAM: white matter associated microglia; LDAM: lipid droplet accumulating microglia; HAM: human AD microglia; AD: AD microglia; DAM: disease associated microglia; MGnD: microglia neurodegenerative phenotype; ARM: activated response microglia; MIMS: microglia inflamed in MS; ALS-DAM: disease associated microglia in ALS; PD-DAM: disease associated microglia in Parkinson’s disease). ALS-increased DEGs are indicated by red bars with an asterisk (*) (FDR < 0.05 with SMD > 0.80). Microglia marker genes were previously reported by [82]. (**B**,**D**,**F**,**H**) Venn diagrams. Overlap is shown between meta-analysis ALS-increased DEGs and microglia-associated genes. *p*-values (bottom) were generated using Fisher’s exact test. (**C**,**E**,**G**,**H**) ALS-increased DEGs associated with Venn diagram overlap regions. Gene labels with magenta font are expressed more highly in microglia than any other spinal cord cell type (based on snRNA-seq data, GSE222322). Bars denote meta-analysis SMD estimates (left axis). Black circles represent effect size estimates (right axis) and *p*-values (see legend) reported by primary study authors. In (**B**,**C**), ALS-increased DEGs are compared to genes within the cross-disease-associated microglia (CDAM) cluster (see Table S4 from [83]). In (**D**,**E**) ALS-increased DEGs are compared to genes having higher expression in microglia from patients with neurological disease relative to control individuals in Human Microglia Atlas (HuMicA) samples (see Supplementary Data 6 from [84]). In (**F**,**G**), ALS-increased DEGs are compared to those having higher expression in DAM versus homeostatic microglia cell populations from SOD1-G93A mouse spinal cords (see Table S6 from [85]). In (**H**,**I**), ALS-increased DEGs are compared to genes having elevated expression in SOD1-G93A microglia (endstage phenotype) compared to wild type microglia (see Table S3 from [86]).

Several ALS-increased DEGs were known transcriptional biomarkers for disease-associated microglia phenotypes (Figure 5A) [82]. These include, for example, marker genes for disease-associated microglia (DAM), the microglia neurodegenerative phenotype (MGnD), and microglia inflamed in multiple sclerosis (e.g., *APOE*, *CLEC7A*, *TREM2*, *CD68*; Figure 5A) [82]. On the other hand, markers linked to homeostatic microglia [82] were not among ALS-increased DEGs and only weakly increased in the ALS meta-analysis (e.g., *TMEM119*, *P2RY12*, *P2RY13*, *CX3CR1*; Figure 5A). There was significant overlap between ALS-increased DEGs and signatures linked to multiple neurodegenerative conditions, including the recently characterized cross-disease-associated microglia module (CDAM) (Figure 5B) [83] and genes with increased expression in disease samples from the Human Microglia Atlas (HuMicA) (Figure 5D) [84]. ALS-increased DEGs linked to these disease-related microglia phenotypes included *GPNMB*, *APOE*, *LTA4H*, *PSAP* and *ASA11* (Figure 5C,D). There was also significant overlap between ALS-increased DEGs and orthologues of microglia-expressed genes identified from SOD1-G93A mouse spinal cords, including genes up-regulated in disease-associated microglia from this model (Figure 5F) [85] and genes having increased expression in SOD1-G93A microglia (endstage disease) as compared to wild type mice (Figure 5H) [86]. Several of the ALS-increased DEGs linked to SOD1-G93A microglia in these ways (Figure 5G,I) were also part of the above-mentioned CDAM/HuMicA signatures linked to neurogenerative conditions in humans (Figure 5C,E) (e.g., *GPNMB*, *APOC1*, *APOE*, *CAPG*, *LYZ*).

### 3.8. ALS-Decreased DEGs Are Most Strongly Expressed by Mature Oligodendrocyte Phenotypes

**Figure 6:**
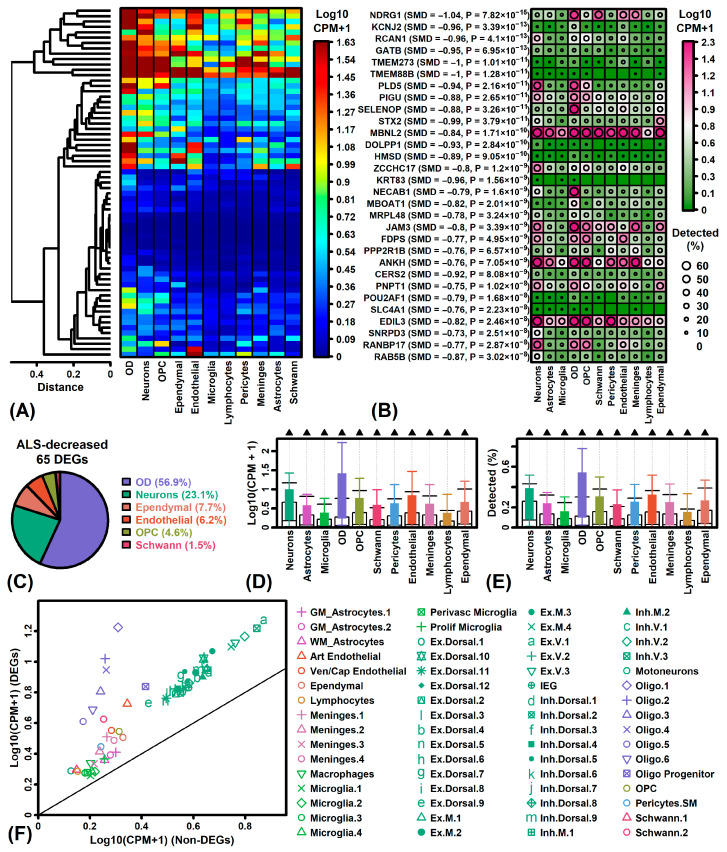
ALS-decreased DEGs and their expression in human spinal cord cell types (GSE190442). (**A**) Cluster analysis. The heatmap shows the average expression of ALS-decreased DEGs across 11 cell type categories. DEGs have been hierarchically clustered using average linkage and the Euclidean distance. (**B**) Top-ranked ALS-decreased DEGs. The heatmap shows top ALS-decreased DEGs and their average expression across 11 cell type categories. The heatmap color corresponds to the average expression and circles indicate the percentage of nuclei with detectable expression. (**C**) Percentage of DEGs assigned to each cell type category. ALS-decreased DEGs were assigned to the cell type category for which average expression was highest. (**D**) Expression level of DEGs versus non-DEGs by cell type category. (**E**) Percentage of nuclei with detectable expression in DEGs versus non-DEGs by cell type category. In (**D**,**E**), boxes outline the middle 50% of values (whiskers: 10th to 90th percentiles). Clear boxes (background) correspond to non-DEGs whereas colored boxes correspond to DEGs. Filled triangles (top margin) denote cases in which DEG expression is significantly greater than non-DEGs (up-triangles) or significantly less than non-DEGs (down-triangles) (Wilcoxon rank sum test, FDR < 0.05). (**F**) Average expression of DEGs versus non-DEGs by cell type sup-population. A different symbol is used for each sub-population whereas all sub-populations within the same category share the same color.

ALS-decreased DEGs had the highest expression in oligodendrocytes and neurons from normal spinal cord samples (Figure 6A). Expression of *SELENOP* and *NECAB1*, for example, was higher and more often detected in oligodendrocytes as compared to other cell types (Figure 6B). More than half of ALS-decreased DEGs (56.9%) were most highly expressed by oligodendrocytes (Figure 6C). On average, ALS-decreased DEGs had significantly higher expression than non-DEGs in all cell types (Figure 6D,E). However, this trend was exaggerated with respect to oligodendrocytes and present with respect to multiple oligodendrocyte subpopulations (Figure 6F).

Oligodendrocytes are a heterogenous population having diverse transcriptional phenotypes in health and disease. Expression of signature genes linked to 50 oligodendrocyte phenotypes [87] exhibited a spectrum of patterns, with some phenotypes predominantly associated with ALS-decreased genes and others mostly associated with ALS-increased genes (Figure S28A). For example, genes linked to previously described MOL5 [88] and Int3 [89] phenotypes overlapped significantly with ALS-decreased DEGs such as *DHCR24*, *NDRG1*, *ZDHHC20* and *SYNJ2* (Figure S28B–E). On the other hand, genes linked to immune ODs (ImOLs) [90] and Int6 [89] phenotypes often had ALS-increased expression and included several increased DEGs such as *APOC1*, *APOE*, *MSR1* and *NRP2* (Figure S28F–I).

### 3.9. ALS-Increased DEGs Are Expressed in Dorsal/Lateral White Matter Whereas Decreased DEGs Are Expressed in Ventral/Lateral White Matter

**Figure 7:**
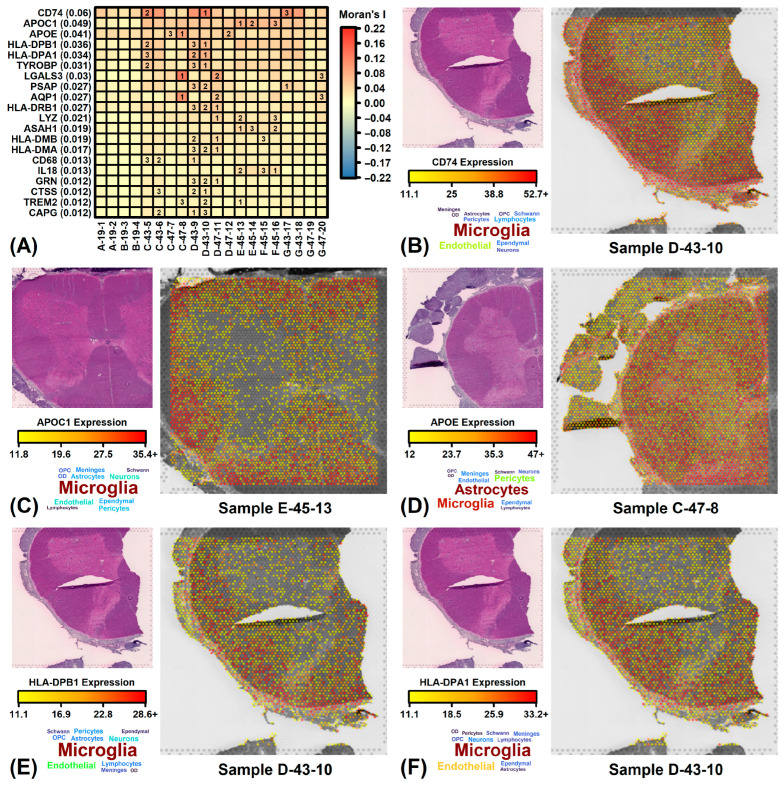
ALS-increased DEGs with high spatial heterogeneity in normal human spinal cord (GSE222322). (**A**) Moran’s I statistic. The heatmap shows ALS-increased DEGs with the highest average Moran’s I statistic across 20 tissue sections (10x Genomics Visium array). The average Moran’s I statistic is listed in parentheses for each gene (left margin). The top 3 samples with the highest Moran’s I statistic for each gene are indicated in each row. (**B**) *CD74* expression (sample D-43-10). (**C**) *APOC1* expression (sample E-45-13). (**D**) *APOE* expression (sample C-47-8). (**E**) *HLA-DPB1* expression (sample D-43-10). (**F**) *HLA-DPA1* expression (sample D-43-10). In (**B**–**F**), the raw H&E image is shown (upper left) alongside the same image overlaid with spots color-coded based upon gene expression (see scale). Colors indicate expression of the gene based upon SCT-normalized expression values scaled to the [0, 100] interval. The word cloud (bottom left) indicates average expression of the gene among spinal cord cell types, with larger font sizes used to denote cell types having relatively higher expression of the indicated gene.

Spatial expression of ALS-increased DEGs was compared to that of non-DEGs in spinal cord sections from normal individuals (Figure S29). ALS-increased DEGs often had relatively increased expression in white matter (Figure S29). There was often prominence of ALS-increased DEG expression in the dorsal and lateral white matter (e.g., see Figure S29E,F,H,I,J,N,P,Q and R). ALS-increased DEGs with greatest spatial heterogeneity on average among spinal cord sections included *CD74*, *APOC1*, *APOE*, *HLA-DPB1* and *HLA-DPA1* (Figure 7A). These genes were most highly expressed by microglia and/or astrocytes with greater white matter expression (Figure 7B–F). Expression of *HLA-DPB1* and *HLA-DPA1* was strongest within the dorsal/lateral white matter (Figure 7E,F).

ALS-decreased DEGs were also more highly expressed in white compared to gray matter (Figure S30). However, there was frequent prominence of decreased DEG expression in the ventral/lateral white matter, whereas such genes had lower dorsal/medial white matter expression (e.g., see Figure S30E–G,I,L,Q,R,S). ALS-decreased DEGs with the most prominent spatial heterogeneity on average included *SELENOP*, *STMN1*, *KLC1*, *DHCR24*, *EDIL3* and *RAPGEF5* (Figure S31A), all of which were highly expressed by oligodendrocytes and neurons (Figure S31B–F). Expression of *STMN1* and *DHCR24* was sharply increased in white matter (Figure S31B,D), whereas *EDIL3* and *RAPGEF5* followed a similar but less prominent spatial pattern (Figure S31E,F). Interestingly, an inverse pattern was seen for *KLC1*, which was predominantly expressed in gray matter (ventral > dorsal; Figure S31C).

### 3.10. ALS-Increased Genes Are Weakly Expressed in ALS Cord but Are Most Prominent in Ventral/Lateral White Matter

**Figure 8:**
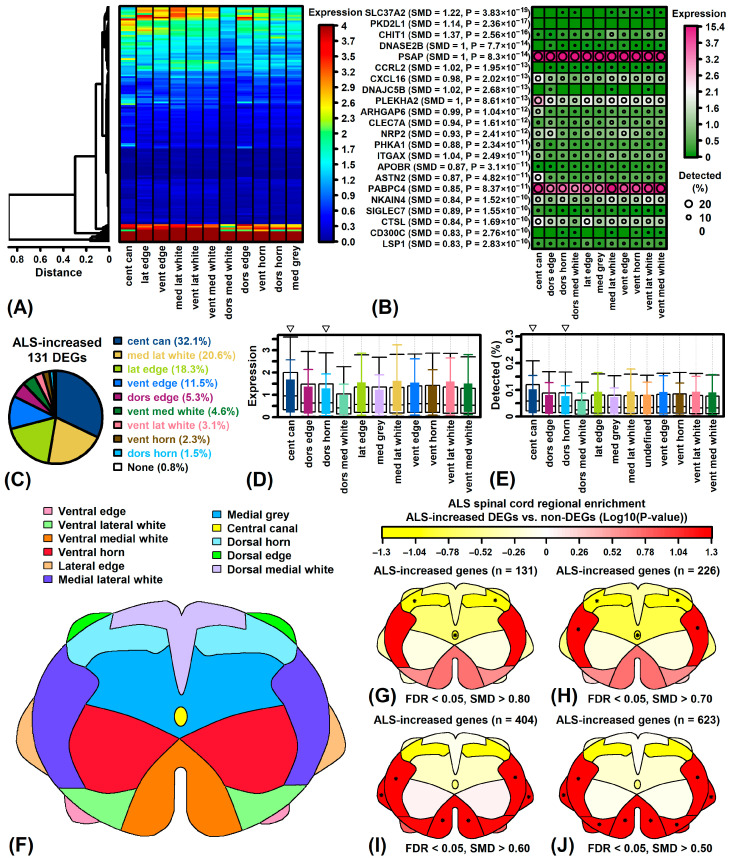
ALS-increased DEGs and their regional expression in ALS patient cervical/lumbar cord segments [44]. (**A**) Cluster analysis. The heatmap shows the average expression of ALS-increased DEGs across 11 spinal cord regions. DEGs have been hierarchically clustered using average linkage and the Euclidean distance. (**B**) Top-ranked ALS-increased DEGs. The heatmap shows top ALS-increased DEGs and their average expression across 11 spinal cord regions. The heatmap color corresponds to average expression and circles indicate the percentage of regional spots with detectable expression. (**C**) Percentage of DEGs assigned to each spinal cord region. ALS-increased DEGs were assigned to the region for which average expression was highest. (**D**) Expression level of DEGs versus non-DEGs by region. (**E**) Percentage of regional spots with detectable expression among DEGs versus non-DEGs. In (**D**,**E**), boxes outline the middle 50% of values (whiskers: 10th to 90th percentiles). Clear boxes (background) correspond to non-DEGs whereas colored boxes correspond to DEGs. Open triangles (top margin) denote cases in which DEG expression is significantly greater than non-DEGs (up-triangles) or significantly less than non-DEGs (down-triangles) (Wilcoxon rank sum test, *p* < 0.05). (**F**) Spinal cord regions (see legend). (**G**–**J**) Relative enrichment of DEG expression by region. Analyses were performed using ALS-increased genes (FDR < 0.05) with SMD > 0.80 (**G**), SMD > 0.70 (**H**), SMD > 0.60 (**I**) and SMD > 0.50 (**J**). The number of genes analyzed is indicated for each case (top margin). For each region, expression of DEGs was compared to non-DEGs (Wilcoxon rank sum test) and colors reflect the Log10(*p*-value) obtained in each comparison (greater than zero if DEG expression > non-DEG expression; less than zero if DEG expression < non-DEG expression). An asterisk (*) is used to denote regions for which DEG expression differs significantly from non-DEG expression (*p* < 0.05, Wilcoxon rank sum test).

ALS-increased DEGs were expressed at low levels in the ALS spinal cord (< 2% of the maximally expressed gene) and usually their expression was detectable at fewer than 20% of spots assayed (Figure 8A,B). Spatial heterogeneity was limited although *PLEKHA2* had relatively higher expression in central canal (Figure 8A,B). Expression of increased DEGs was highest in the central canal, medial lateral white matter and lateral edge (Figure 8C). In relative terms, however, in comparison to non-DEG expression, ALS-increased DEG expression was higher in the medial lateral white matter, with lower expression in the central canal and dorsal horn (Figure 8D–G). Among ALS-increased genes having moderate effect sizes (i.e., FDR < 0.05 with SMD > 0.50, SMD > 0.60 or SMD > 0.70), expression was significantly increased within the lateral edge and medial lateral white matter of ALS patient cords (*p* < 0.05) with a trend towards increased expression in the ventral medial/lateral white matter as well (Figure 8H–J). These trends were illustrated by ALS-increased genes having the greatest spatial heterogeneity, such as *APOE*, *GPNMB*, *APOC1* and *AQP1* (Figure S32A). These genes are most strongly expressed by microglia or astrocytes and in ALS patients their expression is most prominent in the lateral/ventral white matter (Figure S32F–H).

ALS-decreased DEGs were expressed at higher absolute levels in the ALS spinal cord with many detected at more than 20% of spots assayed (Figure S33A,B). Decreased DEGs had highest expression in central canal, ventral lateral white matter and lateral edge (Figure S33C) although DEGs as a group had higher expression than non-DEGs in each region examined (Figure S33E). Decreased DEGs having the greatest spatial heterogeneity included *SELENOP*, *KLC1*, *EDIL3* and *STMN1* (Figure S34A). *KLC1* expression was highest in gray matter and was expressed most highly by neurons (Figure S34C,G). On the other hand, *SELENOP* and *EDIL3* were most prominently in white matter and expressed at high levels in oligodendrocytes (Figure S34F,H).

## 4. Discussion

**Figure 9:**
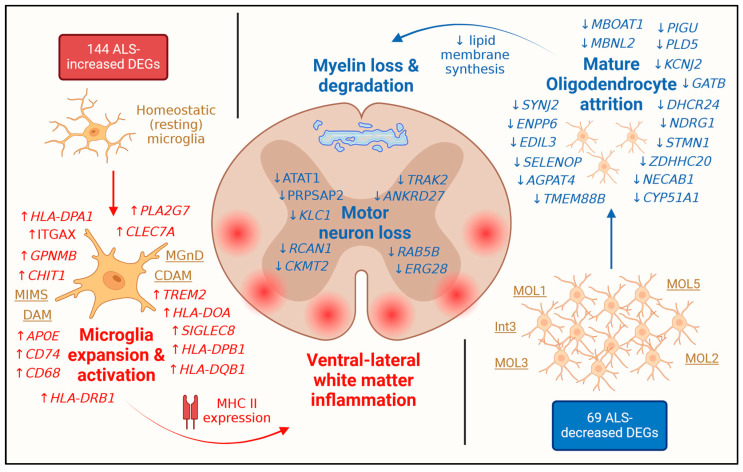
Gene expression shifts in bulk-processed post-mortem spinal cord from ALS patients are consistent with microglia expansion, mature oligodendrocyte attrition and motor neuron loss. ALS-increased DEGs support a model of end-stage disease in which homeostatic (resting) microglia undergo expansion and conversion to an inflammatory phenotype similar to that seen in other neurodegenerative conditions (e.g., MIMS, DAM, CDAM, MGnD). Such phenotypes exhibit increased expression of MHC class II proteins (e.g., *HLA-DBP1*, *HLA-DQB1*, *HLA-DPA1*). This leads to the increased abundance of inflammatory microglia transcripts within the ventral-lateral white matter. ALS-decreased DEGs support a process of oligodendrocyte attrition and loss of mature phenotypes such as MOL5, MOL2 and Int3. This results in decreased synthesis of lipid membrane components and myelin degradation within the white matter. These processes are concurrent with loss of neuron-expressed transcripts within the gray matter, such as kinesin light chain 1 (*KLC1*), likely reflecting motor neuron death.

ALS is a heterogeneous disease and large sample sizes are needed to establish the key molecular and histological features distinguishing tissues from patients and normal controls. Prior studies of post-mortem spinal cord have frequently been limited with respect to sample size [18,19,20,21,22] with loss of statistical power due to the relatively small size of control cohorts [17]. These studies have also utilized varying expression profiling platforms, each of which may be subject to unique patterns of differential expression bias [91], and most published studies have not cross-compared their results to prior datasets. This study used mixed effect linear models [42] and meta-analysis [73] to robustly identify DEGs from 6 prior expression profiling datasets [17,18,19,20,21,22]. This approach moderated patterns of differential expression bias related to mRNA abundance, gene length and GC content (Figures S16–S18). No single mRNA was universally increased or decreased in all ALS spinal cord samples, reflecting the heterogenous nature of the disease [37]. It was possible, however, to identify robust DEGs with consistent trends in all studies and spinal cord regions (cervical, thoracic, lumbar). Analysis of these genes suggests ways in which the spinal cord is reshaped by end-stage ALS, both in terms of cellular composition and the spatial distribution of resident or infiltrating cell types (Figure 9).

The largest prior transcriptome study of post-mortem ALS spinal cord was performed using NYGC ALS Consortium data and evaluated 380 samples total from 203 individuals (154 ALS, 49 CTL), leading to identification of 7349, 256 and 4694 DEGs (FDR < 0.05) with respect to the cervical, thoracic and lumbar regions, respectively [17]. The current meta-analysis incorporated these prior data with an aggregate of 569 samples from 348 individuals (262 ALS, 86 CTL) and identified 213 DEGs (144 ALS-increased, FDR < 0.05, SMD > 0.80; 69 ALS-decreased, FDR < 0.05, SMD < −0.80). Most of these DEGs were not significantly altered in each spinal cord region from the NYGC ALS consortium analysis [17] (Figure S22) and there are multiple explanations for this. First, the current study used mixed effect linear models to allow data from each spinal cord region to be combined into a single test for differential expression, rather than testing for differential expression with respect to each region separately. Second, differential expression models in the current study included fewer covariates as compared to the prior analysis [17], which was carried out to guard against model overfitting and multicollinearity [92,93,94]. Third, the current study used a random effect meta-analysis to combine signals from multiple datasets generated by different expression profiling technologies. Fourth, effect size in the current study was estimated based upon standardized mean difference (SMD), rather than fold-change, and thus a different effect size threshold criterion was necessarily applied to define DEGs. While the combination of data from multiple studies and platforms likely increased technical and clinical variation, this approach also generated a larger sample size and moderated sources of differential expression bias (Figures S16–S18). This should in turn facilitate more reproducible findings as future studies emerge and utilize new clinical samples and/or expression profiling technologies [95,96].

DEGs from the current study had higher expression than non-DEGs in white matter from normal individuals and ALS patients (Figures S29 and S30). Spinal cord white matter contains axons organized into descending corticospinal tracts to propagate signals that control voluntary motor activity [97,98]. Although classically considered a disease of motor neurons, pathological white matter changes in ALS may precede loss of motor neurons [99,100]. Such microstructural changes include loss of myelin volume [101] along with specific corticospinal tract signals detectable by magnetic resonance imaging [102,103,104,105,106]. Based on spatial transcriptomic data [43,44], ALS-increased DEGs predominantly expressed in white matter include *CD74*, *APOC1*, *HLA-DPB1*, *HLA-BPA1* and *GPNMB*. Likewise, ALS-decreased genes with abundant white matter expression include *STMN1* and *DHCR24*. The distribution of ALS-increased DEG expression differed between ALS patients and normal individuals, with stronger expression in the dorsal/lateral white matter of normal individuals (Figure S29) but more ventral/lateral expression among those with ALS (Figure 8). This may reflect a heightened presence of neuroinflammatory cells near the anterior corticospinal tract and anterior horn cells, which in prior work has been correlated with rapid disease progression [107].

Most ALS-increased DEGs (51.4%) from this study were expressed at higher levels by microglia than any other spinal cord cell type (Figure 4). This finding agrees with the prior NYGC ALS Consortium data analysis [17] and is consistent with white matter predominance of ALS-increased DEG expression. Several ALS-increased DEGs had previously been recognized as markers for microglia phenotypes detected in other neurodegenerative diseases (e.g., *GPNMB*, *APOE*, *LTA4H*) [83,84]. Certain of these DEGs were orthologous to mouse genes having increased expression in spinal cord microglia from SOD1-G93A mice or associated with a disease-associated microglia phenotype identified from this model (e.g., *APOC1*, *CAPG*, *LYZ*) [85,86]. These findings characterize a microglia phenotype that is linked to ALS but also shared, at least in part, by other forms of neurodegenerative disease. In recent years, it has become clear that microglia are too complex to be classified using a homeostatic/surveillant (M1) versus protective (M2) dichotomy [108], but rather more complex classification systems are needed to capture the range of microglia states that exist in healthy and diseased tissues [82]. Along these lines, findings from this analysis identify features of an ALS-emergent microglia phenotype, which may include expression of MHC class II glycoproteins as previously identified in other neurodegenerative conditions [109,110,111,112,113,114]. Such diverse microglia forms may have disease-promoting, neuroprotective or context-dependent functions. A good example may be glycoprotein non-metastatic melanoma protein B (*GPNMB*) [115], which encodes a transmembrane protein detected in microglia and motor neurons from early-stage SOD1-G93A mice [116,117]. Prior work demonstrated a neuroprotective role for *GPNMB* in SOD1-G93A mice as well as other neurodegenerative disease models [117,118,119]. On the other hand, an inverse relationship has been observed between *GPNBM* levels in CSF and ALS patient survival [77,120]. Microglia phenotypes and their marker genes may thus contribute to ALS pathology in complex ways that cannot be characterized by a simple narrative.

Astrocytes have diverse supportive roles in the healthy central nervous system but in response to injury will undergo reactive gliosis and develop neurotoxic properties [121]. Several ALS-increased DEGs did have strong and detectable expression in astrocytes, such as *PSAP*, *PHKA1*, *ASTN2* and *APOE*, although as a group increased DEGs did not differ from non-DEGs with regard to their expression in astrocyte populations. Astrocyte gene expression was discerned from a prior study that used scRNA-seq to characterize gene expression in post-mortem spinal cords from neurologically normal individuals [43]. It is possible, however, that ALS astrocytes develop an aberrant phenotype that is predominantly observed in disease states but not in the absence of disease [122]. Astrocytes may therefore contribute to differential expression in bulk-processed ALS spinal cords primarily by shifting towards a reactive phenotype triggered within an inflammatory microenvironment.

Oligodendrocytes (ODs) generate a myelin sheath plasma membrane that provides nutritional support and insulates CNS axons to facilitate saltatory nerve conduction [123,124]. OD dysfunction may contribute to multiple forms of neurodegenerative disease including ALS [125]. Although demyelinating lesions in ALS are not characteristically seen by MRI imaging [126], demyelination has been observed in post-mortem ALS tissues [127,128], in which ODs exhibit a high burden of cytoplasmic inclusions, potentially reflecting defects in mRNA transport [129] and lipid metabolism [130]. These processes may be enhanced among those with *C9orf72* mutations [131]. In this study, most ALS-decreased DEGs were expressed more highly by ODs than any other cell type in normal spinal cord (e.g., *SELENOP* and *NECAB1*; Figure 6B,C). More specifically, ALS spinal cords appear deficient in the expression of genes linked to certain OD phenotypes, such as the MOL5 phenotype [88], which has been localized to corticospinal tract [132] and is one of the predominant mature resting-state OD phenotypes observed in the mouse central nervous system. Other OD phenotypes linked to ALS-decreased signature genes include the MOL1, MOL2 and MOL3 subtypes described by Zeisel et al. [133] and the Int3 subtype described by Sadick et al. [89] (Figure S28A). Despite loss of OD-related gene expression in ALS spinal cords, however, signature genes for some OD phenotypes, such as immune ODs (ImOLs) [90], are increased in post-mortem tissues (e.g., *APOC1*, *APOE*, *MSR1*) (Figure S28A). This underscores the inherent heterogeneity of OD phenotypes and the possibility that some minority OD subtypes undergo expansion within the ALS spinal cord, despite the broader trend towards loss of OD-associated gene expression.

## 5. Conclusions

Gene expression analysis of post-mortem tissue can provide insights into the molecular and histological abnormalities of end-stage disease. This approach has now been applied on a large scale to analyze spinal cord segments from ALS patients and normal individuals, with results available from multiple studies that have utilized varying statistical methods and expression profiling platforms [17,18,19,20,21,22]. This study reports findings from a meta-analysis of expression datasets that together included 569 samples (454 ALS, 115 CTL) from 348 individuals (262 ALS, 86 CTL), representing the largest aggregate sample size of any analysis performed to date. The analysis identified 144 ALS-increased DEGs (FDR < 0.05, SMD > 0.80) and 69 ALS-decreased DEGs (FDR < 0.05, SMD < −0.80) and evaluated expression of these genes in prior snRNA-seq and spatial transcriptomic datasets [43,44]. ALS-increased DEGs were highly expressed by microglia and include marker genes previously linked to neurodegenerative diseases [83,84] and the SOD1-G93A mouse model [85,86]. In contrast, ALS-decreased DEGs were highly expressed by mature oligodendrocytes, such as the MOL5 phenotype [88], and enriched for annotations related to myelin production. Both increased and decreased DEGs were primarily expressed by white matter, with prominence of increased DEGs expression in the ventral aspect of ALS cord samples. These findings support a model in which ALS progression disproportionately impacts the spinal cord white matter, with disease-related changes driven by expansion of an ALS-emergent microglia phenotype and concurrent attrition of mature oligodendrocytes and their myelin-generating ability (Figure 9).

## Figures and Tables

**Table 1:** Meta-analysis datasets. Datasets used for the meta-analysis are listed in the table below. All data evaluated gene expression in bulk-processed post-mortem spinal cord from ALS and CTL individuals. Study identifiers (first column) represent accessions under which processed and/or raw data can be obtained (see footnotes).

Identifier	*n* (ALS) ^1^	*n* (CTL) ^1^	Gene Count ^2^	ALS-Increased ^3^	ALS-Decreased ^4^
GSE137810-N ^a^	253 (137)	51 (35)	16,044	1000	810
GSE137810-H ^b^	150 (75)	27 (17)	15,936	291	93
GSE255683 ^c^	10 (10)	10 (10)	15,204	0	0
SRP064478 ^d^	7 (7)	8 (8)	14,791	0	0
GSE26927 ^e^	10 (9)	10 (7)	13,634	0	0
E-MTAB-8635 ^f^	24 (24)	9 (9)	16,921	0	0

^1^ Number of ALS and CTL samples. The number of unique patients represented among samples is given in parentheses. ^2^ Number of protein-coding genes included in differential expression analyses. ^3^ Number of ALS-increased genes identified (FDR < 0.05, SMD > 0.80). ^4^ Number of ALS-decreased genes identified (FDR < 0.05, SMD < −0.80). ^a^ Sequence data generated using the Illumina NovaSeq 6000 platform (GPL24676). Samples were obtained from the cervical, thoracic and lumbar regions. Raw and processed data have been submitted to GEO (GSE137810). ^b^ Sequence data generated using the Illumina HiSeq 2500 platform (GPL16791). Samples were obtained from the cervical, thoracic and lumbar regions. Raw and processed data have been submitted to GEO (GSE137810). ^c^ Sequence data generated using the Illumina NovaSeq 6000 platform (GPL24676). Processed data have been submitted to GEO (GSE255683). Samples were obtained from the lumbar region. Raw and processed data have been submitted to the European Genome-Phenome Archive (study no. EGAS50000000575; dataset nos. EGAD50000000820 and EGAD50000000833). ^d^ Sequence data were generated using the Illumina NextSeq 500 platform (GPL18573). Samples were obtained from the cervical region. Raw files have been submitted to the Sequence Read Archive (SRP064478). ^e^ Microarray data were generated using the Illumina humanRef-8 v2.0 expression beadchip array (GPL6255). Samples were obtained from the cervical region. Raw and processed files have been submitted to GEO (GSE26927). ^f^ Microarray data were generated using the Agilent-014850 Whole Human Genome Microarray 4x44K. Samples were obtained from the lumbar region. Raw data files were submitted to the ArrayExpress database (E-MTAB-8635).

## Data Availability

Datasets analyzed in the current study are available from the Gene Expression Omnibus (GSE137810, GSE255683, GSE26927), ArrayExpress (E-MTAB-8635f), or Sequence Read Archive (SRP064478) databases.

## Supplementary Materials

The following supporting information can be downloaded at: https://www.mdpi.com/article/10.3390/neurosci6030065/s1, Supplementary Material File S1: ALS-increased DEGs and enrichment analyses; Supplementary Material File S2: ALS-decreased DEGs and enrichment analyses; Figure S1: GSE137810 mapping results (*n* = 481 samples); Figure S2: GSE137810 covariates and variable importance (*n* = 481 samples); Figure S3: Hierarchical cluster analyses; Figure S4: GSE255683 read mapping results (*n* = 20 samples); Figure S5: SRP064478 read mapping results (*n* = 15 samples); Figure S6: Microarray signal intensity distributions and number of detected genes (GSE26927); Figure S7: Microarray signal intensity distributions and number of detected genes (E-MTAB-8635); Figure S8: Microarray pseudoimages (E-MTAB-8635); Figure S9: Microarray pseudoimages (E-MTAB-8635); Figure S10: Spatial transcriptomic quality control metrics for human lumbar spinal cord samples (GSE222322, 10x Genomics Visium array); Figure S11: Spatial transcriptomic molecular counts for human lumbar spinal cord samples (GSE222322, 10x Genomics Visium array); Figure S12: Spatial transcriptomic gene counts for human lumbar spinal cord samples (GSE222322, 10x Genomics Visium array); Figure S13: Spatial transcriptomic mitochondrial molecular count percentage for human lumbar spinal cord samples (GSE222322, 10x Genomics Visium array); Figure S14: Principal component analyses; Figure S15: Analysis of differential expression *p*-values (ALS vs. CTL); Figure S16: Differential expression volcano and MA plots; Figure S17: Differential expression associations with gene length and GC content; Figure S18: Differential expression meta-analyses (ALS vs. CTL); Figure S19: SLC37A2 expression summary; Figure S20: NDRG1 expression summary; Figure S21: GO BP term cluster analysis; Figure S22: Comparison of meta-analysis DEGs to those previously identified from NYGC ALS Consortium data [17]; Figure S23: Meta-analysis comparison of ALS gene dysregulation in human spinal cord (bulk tissue vs. LCM-MN); Figure S24: Overlap between DEGs and proteins dysregulated in ALS spinal cord; Figure S25: Overlap between DEGs and genes dysregulated in whole blood from ALS patients; Figure S26: Overlap between DEGs and genes near ALS GWAS loci; Figure S27: ALS-increased DEG comparison to ALS-increased microglia genes from motor/premotor cortical gray matter (snRNA-seq study by Limone et al. [31]); Figure S28: Oligodendrocyte (OD) phenotypes; Figure S29: Regional enrichment of ALS-increased DEG expression in normal human spinal cord (GSE222322, 10x Genomics Visium array); Figure S30: Regional enrichment of ALS-decreased DEG expression in normal human spinal cord (GSE222322, 10x Genomics Visium array); Figure S31: ALS-decreased DEGs with high spatial heterogeneity in normal human spinal cord (GSE222322); Figure S32: ALS-increased DEGs with high spatial heterogeneity in ALS patient spinal cord sections [44]; Figure S33: ALS-decreased DEGs and their regional expression in ALS patient cervical/lumbar cord segments [44]; Figure S34: ALS-decreased DEGs with high spatial heterogeneity in ALS patient spinal cord sections [44].

## Abbreviations

The following abbreviations are used in this manuscript:

ATM	axon tract associated microglia
ARM	activated response microglia
BP	biological process
CDAM	cross-disease-associated microglia module
CDF	cumulative distribution function
CTL	control
CPM	count per million
DAM	disease-associated microglia
DEG	differential expressed gene
FDR	false discovery rate
FPKM	fragments per kilobase of transcript per million mapped reads
GO	gene ontology
GWAS	genome-wide association study
HAM	human AD microglia
HuMicA	human microglia atlas
ImOLs	immune oligodendrocytes
LCM	laser capture microdissection
LCM-MN	laser capture microdissected motor neuron
LDAM	lipid droplet accumulating microglia
LRT	likelihood ratio test
M1	homeostatic/surveillant microglia
M2	protective microglia
MGnD	microglia neurodegenerative phenotype
MIMS	microglia inflamed in MS
NYGC	New York genome center
OD	oligodendrocyte
OPC	oligodendrocyte precursor cells
PAM	proliferative associated microglia
PC	principal component
PD-DAM	disease associated microglia in Parkinson’s disease
RIN	RNA integrity number
SMD	standardized mean difference
SOM	self-organizing map
snRNA-seq	single-nucleus RNA-seq
WAM	white matter associated microglia

## References

[1] Brown R.H., Al-Chalabi A. (2017). Amyotrophic Lateral Sclerosis. N. Engl. J. Med..

[2] Wu J.Y., Ye S., Liu X.Y., Xu Y.S., Fan D.S. (2025). The burden of upper motor neuron involvement is correlated with the bilateral limb involvement interval in patients with amyotrophic lateral sclerosis: A retrospective observational study. Neural Regen. Res..

[3] Sun H., Yang B., Li Q., Zhu X., Song E., Liu C., Song Y., Jiang G. (2024). Polystyrene nanoparticles trigger aberrant condensation of TDP-43 and amyotrophic lateral sclerosis-like symptoms. Nat. Nanotechnol..

[4] Calderón-Garcidueñas L., Stommel E.W., Torres-Jardón R., Hernández-Luna J., Aiello-Mora M., González-Maciel A., Reynoso-Robles R., Pérez-Guillé B., Silva-Pereyra H.G., Tehuacanero-Cuapa S. (2024). Alzheimer and Parkinson diseases, frontotemporal lobar degeneration and amyotrophic lateral sclerosis overlapping neuropathology start in the first two decades of life in pollution exposed urbanites and brain ultrafine particulate matter and industrial nanoparticles, including Fe, Ti, Al, V, Ni, Hg, Co, Cu, Zn, Ag, Pt, Ce, La, Pr and W are key players. Metropolitan Mexico City health crisis is in progress. Front. Hum. Neurosci..

[5] Khan S., Bano N., Ahamad S., John U., Dar N.J., Bhat S.A. (2025). Excitotoxicity, Oxytosis/Ferroptosis, and Neurodegeneration: Emerging Insights into Mitochondrial Mechanisms. Aging Dis..

[6] Shi P., Wei Y., Zhang J., Gal J., Zhu H. (2010). Mitochondrial dysfunction is a converging point of multiple pathological pathways in amyotrophic lateral sclerosis. J. Alzheimer’s Dis. JAD.

[7] Abyadeh M., Gupta V., Paulo J.A., Mahmoudabad A.G., Shadfar S., Mirshahvaladi S., Gupta V., Nguyen C.T.O., Finkelstein D.I., You Y.Y. (2024). Amyloid-beta and tau protein beyond Alzheimer’s disease. Neural Regen. Res..

[8] Cheng F.Y., Kotha S., Fu M., Yang Q., Wang H., He W.W., Mao X.B. (2024). Nanozyme enabled protective therapy for neurological diseases. Nano Today.

[9] Zhang H., Guo H.Z., Li D.N., Zhang Y.L., Zhang S.N., Kang W.Y., Liu C., Le W.D., Wang L., Li D. (2024). Halogen doped graphene quantum dots modulate TDP-43 phase separation and aggregation in the nucleus. Nat. Commun..

[10] Liu Z.H., Zhang H., Lu K.L., Chen L., Zhang Y.Q., Xu Z.W., Zhou H.S., Sun J.F., Xu M.Y., Ouyang Q. (2024). Low-intensity pulsed ultrasound modulates disease progression in the SOD1G93A mouse G93A mouse model of amyotrophic lateral sclerosis. Cell Rep..

[11] Zhang Y.G., Xia Y.L., Sun J. (2024). Probiotics and microbial metabolites maintain barrier and neuromuscular functions and clean protein aggregation to delay disease progression in TDP43 mutation mice. Gut Microbes.

[12] Yan J.S., Chen H.X., Zhang Y., Peng L.H., Wang Z.K., Lan X.Y., Yu S.Y., Yang Y.S. (2024). Fecal microbiota transplantation significantly improved respiratory failure of amyotrophic lateral sclerosis. Gut Microbes.

[13] Huang J.L., Fu Y.L., Wang A.T., Shi K.X., Peng Y.D., Yi Y., Yu R.H., Gao J.C., Feng J.F., Jiang G. (2024). Brain Delivery of Protein Therapeutics by Cell Matrix-Inspired Biomimetic Nanocarrier. Adv. Mater..

[14] Taylor J.P., Brown R.H., Cleveland D.W. (2016). Decoding ALS: From genes to mechanism. Nature.

[15] Sneha N.P., Dharshini S.A.P., Taguchi Y.H., Gromiha M.M. (2024). Tracing ALS Degeneration: Insights from Spinal Cord and Cortex Transcriptomes. Genes.

[16] Rizzuti M., Sali L., Melzi V., Scarcella S., Costamagna G., Ottoboni L., Quetti L., Brambilla L., Papadimitriou D., Verde F. (2023). Genomic and transcriptomic advances in amyotrophic lateral sclerosis. Ageing Res. Rev..

[17] Humphrey J., Venkatesh S., Hasan R., Herb J.T., de Paiva Lopes K., Küçükali F., Byrska-Bishop M., Evani U.S., Narzisi G., Fagegaltier D. (2023). Integrative transcriptomic analysis of the amyotrophic lateral sclerosis spinal cord implicates glial activation and suggests new risk genes. Nat. Neurosci..

[18] Durrenberger P.F., Fernando F.S., Kashefi S.N., Bonnert T.P., Seilhean D., Nait-Oumesmar B., Schmitt A., Gebicke-Haerter P.J., Falkai P., Grünblatt E. (2015). Common mechanisms in neurodegeneration and neuroinflammation: A BrainNet Europe gene expression microarray study. J. Neural Transm..

[19] Durrenberger P.F., Fernando F.S., Magliozzi R., Kashefi S.N., Bonnert T.P., Ferrer I., Seilhean D., Nait-Oumesmar B., Schmitt A., Gebicke-Haerter P.J. (2012). Selection of novel reference genes for use in the human central nervous system: A BrainNet Europe Study. Acta Neuropathol..

[20] Newton D.F., Yang R., Gutierrez J., Hofmann J.W., Yeh F.L., Biever A., Friedman B.A. (2024). TDP43 proteinopathy exhibits disease, tissue, and context-specific cryptic splicing signatures. bioRxiv.

[21] Brohawn D.G., O’Brien L.C., Bennett J.P. (2016). RNAseq Analyses Identify Tumor Necrosis Factor-Mediated Inflammation as a Major Abnormality in ALS Spinal Cord. PLoS ONE.

[22] La Cognata V., Gentile G., Aronica E., Cavallaro S. (2020). Splicing Players Are Differently Expressed in Sporadic Amyotrophic Lateral Sclerosis Molecular Clusters and Brain Regions. Cells.

[23] Cox L.E., Ferraiuolo L., Goodall E.F., Heath P.R., Higginbottom A., Mortiboys H., Hollinger H.C., Hartley J.A., Brockington A., Burness C.E. (2010). Mutations in CHMP2B in lower motor neuron predominant amyotrophic lateral sclerosis (ALS). PLoS ONE.

[24] Rabin S.J., Kim J.M., Baughn M., Libby R.T., Kim Y.J., Fan Y., Libby R.T., La Spada A., Stone B., Ravits J. (2010). Sporadic ALS has compartment-specific aberrant exon splicing and altered cell-matrix adhesion biology. Hum. Mol. Genet..

[25] Kirby J., Ning K., Ferraiuolo L., Heath P.R., Ismail A., Kuo S.W., Valori C.F., Cox L., Sharrack B., Wharton S.B. (2011). Phosphatase and tensin homologue/protein kinase B pathway linked to motor neuron survival in human superoxide dismutase 1-related amyotrophic lateral sclerosis. Brain A J. Neurol..

[26] Highley J.R., Kirby J., Jansweijer J.A., Webb P.S., Hewamadduma C.A., Heath P.R., Higginbottom A., Raman R., Ferraiuolo L., Cooper-Knock J. (2014). Loss of nuclear TDP-43 in amyotrophic lateral sclerosis (ALS) causes altered expression of splicing machinery and widespread dysregulation of RNA splicing in motor neurones. Neuropathol. Appl. Neurobiol..

[27] Cooper-Knock J., Bury J.J., Heath P.R., Wyles M., Higginbottom A., Gelsthorpe C., Highley J.R., Hautbergue G., Rattray M., Kirby J. (2015). C9ORF72 GGGGCC Expanded Repeats Produce Splicing Dysregulation which Correlates with Disease Severity in Amyotrophic Lateral Sclerosis. PLoS ONE.

[28] Krach F., Batra R., Wheeler E.C., Vu A.Q., Wang R., Hutt K., Rabin S.J., Baughn M.W., Libby R.T., Diaz-Garcia S. (2018). Transcriptome-pathology correlation identifies interplay between TDP-43 and the expression of its kinase CK1E in sporadic ALS. Acta Neuropathol..

[29] Nizzardo M., Taiana M., Rizzo F., Aguila Benitez J., Nijssen J., Allodi I., Melzi V., Bresolin N., Comi G.P., Hedlund E. (2020). Synaptotagmin 13 is neuroprotective across motor neuron diseases. Acta Neuropathol..

[30] Pineda S.S., Lee H., Ulloa-Navas M.J., Linville R.M., Garcia F.J., Galani K., Engelberg-Cook E., Castanedes M.C., Fitzwalter B.E., Pregent L.J. (2024). Single-cell dissection of the human motor and prefrontal cortices in ALS and FTLD. Cell.

[31] Limone F., Mordes D.A., Couto A., Joseph B.J., Mitchell J.M., Therrien M., Ghosh S.D., Meyer D., Zhang Y., Goldman M. (2024). Single-nucleus sequencing reveals enriched expression of genetic risk factors in extratelencephalic neurons sensitive to degeneration in ALS. Nat. Aging.

[32] Li J., Jaiswal M.K., Chien J.F., Kozlenkov A., Jung J., Zhou P., Gardashli M., Pregent L.J., Engelberg-Cook E., Dickson D.W. (2023). Divergent single cell transcriptome and epigenome alterations in ALS and FTD patients with C9orf72 mutation. Nat. Commun..

[33] Swindell W.R. (2024). Meta-analysis of differential gene expression in lower motor neurons isolated by laser capture microdissection from post-mortem ALS spinal cords. Front. Genet..

[34] Toro-Domínguez D., Villatoro-García J.A., Martorell-Marugán J., Román-Montoya Y., Alarcón-Riquelme M.E., Carmona-Sáez P. (2021). A survey of gene expression meta-analysis: Methods and applications. Brief. Bioinform..

[35] Ramasamy A., Mondry A., Holmes C.C., Altman D.G. (2008). Key issues in conducting a meta-analysis of gene expression microarray datasets. PLoS Med..

[36] Campain A., Yang Y.H. (2010). Comparison study of microarray meta-analysis methods. BMC Bioinform..

[37] Grad L.I., Rouleau G.A., Ravits J., Cashman N.R. (2017). Clinical Spectrum of Amyotrophic Lateral Sclerosis (ALS). Cold Spring Harb. Perspect. Med..

[38] Goyal N.A., Berry J.D., Windebank A., Staff N.P., Maragakis N.J., van den Berg L.H., Genge A., Miller R., Baloh R.H., Kern R. (2020). Addressing heterogeneity in amyotrophic lateral sclerosis CLINICAL TRIALS. Muscle Nerve.

[39] Su X.W., Broach J.R., Connor J.R., Gerhard G.S., Simmons Z. (2014). Genetic heterogeneity of amyotrophic lateral sclerosis: Implications for clinical practice and research. Muscle Nerve.

[40] Li X., Wang C.Y. (2021). From bulk, single-cell to spatial RNA sequencing. Int. J. Oral Sci..

[41] Law C.W., Chen Y., Shi W., Smyth G.K. (2014). Voom: Precision weights unlock linear model analysis tools for RNA-seq read counts. Genome Biol..

[42] Vestal B.E., Wynn E., Moore C.M. (2022). LmerSeq: An R package for analyzing transformed RNA-Seq data with linear mixed effects models. BMC Bioinform..

[43] Yadav A., Matson K.J.E., Li L., Hua I., Petrescu J., Kang K., Alkaslasi M.R., Lee D.I., Hasan S., Galuta A. (2023). A cellular taxonomy of the adult human spinal cord. Neuron.

[44] Maniatis S., Äijö T., Vickovic S., Braine C., Kang K., Mollbrink A., Fagegaltier D., Andrusivová Ž., Saarenpää S., Saiz-Castro G. (2019). Spatiotemporal dynamics of molecular pathology in amyotrophic lateral sclerosis. Science (New York NY).

[45] Sollis E., Mosaku A., Abid A., Buniello A., Cerezo M., Gil L., Groza T., Güneş O., Hall P., Hayhurst J. (2023). The NHGRI-EBI GWAS Catalog: Knowledgebase and deposition resource. Nucleic Acids Res..

[46] Malaspina A., Kaushik N., de Belleroche J. (2001). Differential expression of 14 genes in amyotrophic lateral sclerosis spinal cord detected using gridded cDNA arrays. J. Neurochem..

[47] Offen D., Barhum Y., Melamed E., Embacher N., Schindler C., Ransmayr G. (2009). Spinal cord mRNA profile in patients with ALS: Comparison with transgenic mice expressing the human SOD-1 mutant. J. Mol. Neurosci. MN.

[48] Andrés-Benito P., Moreno J., Aso E., Povedano M., Ferrer I. (2017). Amyotrophic lateral sclerosis, gene deregulation in the anterior horn of the spinal cord and frontal cortex area 8: Implications in frontotemporal lobar degeneration. Aging.

[49] D’Erchia A.M., Gallo A., Manzari C., Raho S., Horner D.S., Chiara M., Valletti A., Aiello I., Mastropasqua F., Ciaccia L. (2017). Massive transcriptome sequencing of human spinal cord tissues provides new insights into motor neuron degeneration in ALS. Sci. Rep..

[50] Dangond F., Hwang D., Camelo S., Pasinelli P., Frosch M.P., Stephanopoulos G., Stephanopoulos G., Brown R.H., Gullans S.R. (2004). Molecular signature of late-stage human ALS revealed by expression profiling of postmortem spinal cord gray matter. Physiol. Genom..

[51] Butovsky O., Jedrychowski M.P., Cialic R., Krasemann S., Murugaiyan G., Fanek Z., Greco D.J., Wu P.M., Doykan C.E., Kiner O. (2015). Targeting miR-155 restores abnormal microglia and attenuates disease in SOD1 mice. Ann. Neurol..

[52] Kodama Y., Shumway M., Leinonen R. (2012). The Sequence Read Archive: Explosive growth of sequencing data. Nucleic Acids Res..

[53] Brown J., Pirrung M., McCue L.A. (2017). FQC Dashboard: Integrates FastQC results into a web-based, interactive, and extensible FASTQ quality control tool. Bioinformatics.

[54] TrimGalore. https://github.com/FelixKrueger/TrimGalore.

[55] BBMap Short Read Aligner, and Other Bioinformatic Tools. https://sourceforge.net/projects/bbmap/.

[56] Dobin A., Davis C.A., Schlesinger F., Drenkow J., Zaleski C., Jha S., Batut P., Chaisson M., Gingeras T.R. (2013). STAR: Ultrafast universal RNA-seq aligner. Bioinformatics.

[57] Li H., Handsaker B., Wysoker A., Fennell T., Ruan J., Homer N., Marth G., Abecasis G., Durbin R. (2009). The Sequence Alignment/Map format and SAMtools. Bioinformatics.

[58] Wang L., Wang S., Li W. (2012). RSeQC: Quality control of RNA-seq experiments. Bioinformatics.

[59] Pertea M., Pertea G.M., Antonescu C.M., Chang T.C., Mendell J.T., Salzberg S.L. (2015). StringTie enables improved reconstruction of a transcriptome from RNA-seq reads. Nat. Biotechnol..

[60] Ramsköld D., Wang E.T., Burge C.B., Sandberg R. (2009). An abundance of ubiquitously expressed genes revealed by tissue transcriptome sequence data. PLoS Comput. Biol..

[61] Hart T., Komori H.K., LaMere S., Podshivalova K., Salomon D.R. (2013). Finding the active genes in deep RNA-seq gene expression studies. BMC Genom..

[62] Breiman L. (2001). Random Forests. Mach. Learn..

[63] Anders S., Huber W. (2010). Differential expression analysis for sequence count data. Genome Biol..

[64] Ritchie M.E., Silver J., Oshlack A., Holmes M., Diyagama D., Holloway A., Smyth G.K. (2007). A comparison of background correction methods for two-colour microarrays. Bioinformatics.

[65] Bolstad B.M., Irizarry R.A., Astrand M., Speed T.P. (2003). A comparison of normalization methods for high density oligonucleotide array data based on variance and bias. Bioinformatics.

[66] Li H., Zhu D., Cook M. (2008). A statistical framework for consolidating “sibling” probe sets for Affymetrix GeneChip data. BMC Genom..

[67] Weng L., Dai H., Zhan Y., He Y., Stepaniants S.B., Bassett D.E. (2006). Rosetta error model for gene expression analysis. Bioinformatics.

[68] Sarkans U., Füllgrabe A., Ali A., Athar A., Behrangi E., Diaz N., Fexova S., George N., Iqbal H., Kurri S. (2021). From ArrayExpress to BioStudies. Nucleic Acids Res..

[69] Workman C., Jensen L.J., Jarmer H., Berka R., Gautier L., Nielser H.B., Saxild H.H., Nielsen C., Brunak S., Knudsen S. (2002). A new non-linear normalization method for reducing variability in DNA microarray experiments. Genome Biol..

[70] Hedges L.V. (1981). Distribution theory for Glass’s estimator of effect size and related estimators. J. Educ. Stat..

[71] Hedges L.V., Olkin I. (2014). Statistical Methods for Meta-Analysis.

[72] Lipsey M.W., Wilson D.B. (2001). Practical Meta-Analysis.

[73] Schwarzer G., Carpenter J.R., Rücker G. (2015). Meta-Analysis with R.

[74] Benjamini Y., Hochberg Y. (1995). Controlling the false discovery rate: A powerful and practical approach to multiple testing. J. R. Stat. Soc. B.

[75] Hafemeister C., Satija R. (2019). Normalization and variance stabilization of single-cell RNA-seq data using regularized negative binomial regression. Genome Biol..

[76] Lorenzi L., Chiu H.S., Avila Cobos F., Gross S., Volders P.J., Cannoodt R., Nuytens J., Vanderheyden K., Anckaert J., Lefever S. (2021). The RNA Atlas expands the catalog of human non-coding RNAs. Nat. Biotechnol..

[77] Oeckl P., Weydt P., Thal D.R., Weishaupt J.H., Ludolph A.C., Otto M. (2020). Proteomics in cerebrospinal fluid and spinal cord suggests UCHL1, MAP2 and GPNMB as biomarkers and underpins importance of transcriptional pathways in amyotrophic lateral sclerosis. Acta Neuropathol..

[78] Ramos-Zaldívar H.M., Polakovicova I., Salas-Huenuleo E., Corvalán A.H., Kogan M.J., Yefi C.P., Andia M.E. (2022). Extracellular vesicles through the blood-brain barrier: A review. Fluids Barriers CNS.

[79] Swindell W.R., Kruse C.P.S., List E.O., Berryman D.E., Kopchick J.J. (2019). ALS blood expression profiling identifies new biomarkers, patient subgroups, and evidence for neutrophilia and hypoxia. J. Transl. Med..

[80] Newman A.M., Liu C.L., Green M.R., Gentles A.J., Feng W., Xu Y., Hoang C.D., Diehn M., Alizadeh A.A. (2015). Robust enumeration of cell subsets from tissue expression profiles. Nat. Methods.

[81] Swindell W.R., Johnston A., Voorhees J.J., Elder J.T., Gudjonsson J.E. (2013). Dissecting the psoriasis transcriptome: Inflammatory- and cytokine-driven gene expression in lesions from 163 patients. BMC Genom..

[82] Paolicelli R.C., Sierra A., Stevens B., Tremblay M.E., Aguzzi A., Ajami B., Amit I., Audinat E., Bechmann I., Bennett M. (2022). Microglia states and nomenclature: A field at its crossroads. Neuron.

[83] Guvenek A., Parikshak N., Zamolodchikov D., Gelfman S., Moscati A., Dobbyn L., Stahl E., Shuldiner A., Coppola G. (2024). Transcriptional profiling in microglia across physiological and pathological states identifies a transcriptional module associated with neurodegeneration. Commun. Biol..

[84] Martins-Ferreira R., Calafell-Segura J., Leal B., Rodríguez-Ubreva J., Martínez-Saez E., Mereu E., Pinho E.C.P., Laguna A., Ballestar E. (2025). The Human Microglia Atlas (HuMicA) unravels changes in disease-associated microglia subsets across neurodegenerative conditions. Nat. Commun..

[85] Keren-Shaul H., Spinrad A., Weiner A., Matcovitch-Natan O., Dvir-Szternfeld R., Ulland T.K., David E., Baruch K., Lara-Astaiso D., Toth B. (2017). A Unique Microglia Type Associated with Restricting Development of Alzheimer’s Disease. Cell.

[86] Chiu I.M., Morimoto E.T., Goodarzi H., Liao J.T., O’Keeffe S., Phatnani H.P., Muratet M., Carroll M.C., Levy S., Tavazoie S. (2013). A neurodegeneration-specific gene-expression signature of acutely isolated microglia from an amyotrophic lateral sclerosis mouse model. Cell Rep..

[87] Valihrach L., Matusova Z., Zucha D., Klassen R., Benesova S., Abaffy P., Kubista M., Anderova M. (2022). Recent advances in deciphering oligodendrocyte heterogeneity with single-cell transcriptomics. Front. Cell. Neurosci..

[88] Marques S., Zeisel A., Codeluppi S., van Bruggen D., Mendanha Falcão A., Xiao L., Li H., Häring M., Hochgerner H., Romanov R.A. (2016). Oligodendrocyte heterogeneity in the mouse juvenile and adult central nervous system. Science.

[89] Sadick J.S., O’Dea M.R., Hasel P., Dykstra T., Faustin A., Liddelow S.A. (2022). Astrocytes and oligodendrocytes undergo subtype-specific transcriptional changes in Alzheimer’s disease. Neuron.

[90] Jäkel S., Agirre E., Mendanha Falcão A., van Bruggen D., Lee K.W., Knuesel I., Malhotra D., Ffrench-Constant C., Williams A., Castelo-Branco G. (2019). Altered human oligodendrocyte heterogeneity in multiple sclerosis. Nature.

[91] Swindell W.R., Xing X., Voorhees J.J., Elder J.T., Johnston A., Gudjonsson J.E. (2014). Integrative RNA-seq and microarray data analysis reveals GC content and gene length biases in the psoriasis transcriptome. Physiol. Genom..

[92] Kim J.H. (2019). Multicollinearity and misleading statistical results. Korean J. Anesthesiol..

[93] Yu H., Jiang S., Land K.C. (2015). Multicollinearity in hierarchical linear models. Soc. Sci. Res..

[94] Yoo W., Mayberry R., Bae S., Singh K., He Q.P., Lillard J.W. (2014). A Study of Effects of MultiCollinearity in the Multivariable Analysis. Int. J. Appl. Sci. Technol..

[95] Ioannidis J.P. (2005). Why most published research findings are false. PLoS Med..

[96] Yu L. (2020). RNA-Seq Reproducibility Assessment of the Sequencing Quality Control Project. Cancer Inform..

[97] Lemon R.N. (2008). Descending pathways in motor control. Annu. Rev. Neurosci..

[98] Lemon R. (2024). The Corticospinal System and Amyotrophic Lateral Sclerosis: IFCN handbook chapter. Clin. Neurophysiol. Off. J. Int. Fed. Clin. Neurophysiol..

[99] Zhou T., Ahmad T.K., Gozda K., Truong J., Kong J., Namaka M. (2017). Implications of white matter damage in amyotrophic lateral sclerosis. Mol. Med. Rep..

[100] Fischer L.R., Culver D.G., Tennant P., Davis A.A., Wang M., Castellano-Sanchez A., Khan J., Polak M.A., Glass J.D. (2004). Amyotrophic lateral sclerosis is a distal axonopathy: Evidence in mice and man. Exp. Neurol..

[101] Toko M., Ohshita T., Nakamori M., Ueno H., Akiyama Y., Maruyama H. (2025). Myelin measurement in amyotrophic lateral sclerosis with synthetic MRI: A potential diagnostic and predictive method. J. Neurol. Sci..

[102] Chen H.J., Zhan C., Cai L.M., Lin J.H., Zhou M.X., Zou Z.Y., Yao X.F., Lin Y.J. (2021). White matter microstructural impairments in amyotrophic lateral sclerosis: A mean apparent propagator MRI study. NeuroImage Clin..

[103] Rajagopalan V., Pioro E.P. (2025). Graph theory network analysis reveals widespread white matter damage in brains of patients with classic ALS. Amyotroph. Lateral Scler. Front. Degener..

[104] Desai A.B., Agarwal A., Mohamed A.S., Mohamed K.H., Middlebrooks E.H., Bhatt A.A., Gupta V., Kumar N., Sechi E., Flanagan E.P. (2025). Motor Neuron Diseases and Central Nervous System Tractopathies: Clinical-Radiologic Correlation and Diagnostic Approach. Radiogr. A Rev. Publ. Radiol. Soc. N. Am. Inc..

[105] Almgren H., Mahoney C.J., Huynh W., D’Souza A., Berte S., Lv J., Wang C., Kiernan M.C., Calamante F., Tu S. (2025). Quantifying neurodegeneration within subdivisions of core motor pathways in amyotrophic lateral sclerosis using diffusion MRI. J. Neurol..

[106] Wendebourg M.J., Kesenheimer E., Sander L., Weigel M., Weidensteiner C., Haas T., Madoerin P., Diebold M., Deigendesch N., Neuhaus D. (2024). The Lateral Corticospinal Tract Sign: An MRI Marker for Amyotrophic Lateral Sclerosis. Radiology.

[107] Brettschneider J., Toledo J.B., Van Deerlin V.M., Elman L., McCluskey L., Lee V.M., Trojanowski J.Q. (2012). Microglial activation correlates with disease progression and upper motor neuron clinical symptoms in amyotrophic lateral sclerosis. PLoS ONE.

[108] Xiong X.Y., Liu L., Yang Q.W. (2016). Functions and mechanisms of microglia/macrophages in neuroinflammation and neurogenesis after stroke. Prog. Neurobiol..

[109] Perlmutter L.S., Scott S.A., Barrón E., Chui H.C. (1992). MHC class II-positive microglia in human brain: Association with Alzheimer lesions. J. Neurosci. Res..

[110] Mathys H., Adaikkan C., Gao F., Young J.Z., Manet E., Hemberg M., De Jager P.L., Ransohoff R.M., Regev A., Tsai L.H. (2017). Temporal Tracking of Microglia Activation in Neurodegeneration at Single-Cell Resolution. Cell Rep..

[111] McGeer P.L., Kawamata T., Walker D.G., Akiyama H., Tooyama I., McGeer E.G. (1993). Microglia in degenerative neurological disease. Glia.

[112] Dachet F., Liu J., Ravits J., Song F. (2019). Predicting disease specific spinal motor neurons and glia in sporadic ALS. Neurobiol. Dis..

[113] Imamura K., Hishikawa N., Sawada M., Nagatsu T., Yoshida M., Hashizume Y. (2003). Distribution of major histocompatibility complex class II-positive microglia and cytokine profile of Parkinson’s disease brains. Acta Neuropathol..

[114] Hayes G.M., Woodroofe M.N., Cuzner M.L. (1987). Microglia are the major cell type expressing MHC class II in human white matter. J. Neurol. Sci..

[115] Weterman M.A., Ajubi N., van Dinter I.M., Degen W.G., van Muijen G.N., Ruitter D.J., Bloemers H.P. (1995). NMB, a novel gene, is expressed in low-metastatic human melanoma cell lines and xenografts. Int. J. Cancer.

[116] Barreto-Núñez R., Béland L.C., Boutej H., Picher-Martel V., Dupré N., Barbeito L., Kriz J. (2024). Chronically activated microglia in ALS gradually lose their immune functions and develop unconventional proteome. Glia.

[117] Tanaka H., Shimazawa M., Kimura M., Takata M., Tsuruma K., Yamada M., Takahashi H., Hozumi I., Niwa J., Iguchi Y. (2012). The potential of GPNMB as novel neuroprotective factor in amyotrophic lateral sclerosis. Sci. Rep..

[118] Budge K.M., Neal M.L., Richardson J.R., Safadi F.F. (2018). Glycoprotein NMB: An Emerging Role in Neurodegenerative Disease. Mol. Neurobiol..

[119] Budge K.M., Neal M.L., Richardson J.R., Safadi F.F. (2020). Transgenic Overexpression of GPNMB Protects Against MPTP-Induced Neurodegeneration. Mol. Neurobiol..

[120] Zhu S., Wuolikainen A., Wu J., Öhman A., Wingsle G., Moritz T., Andersen P.M., Forsgren L., Trupp M. (2019). Targeted Multiple Reaction Monitoring Analysis of CSF Identifies UCHL1 and GPNMB as Candidate Biomarkers for ALS. J. Mol. Neurosci. MN.

[121] Liddelow S., Barres B. (2015). SnapShot: Astrocytes in Health and Disease. Cell.

[122] Izrael M., Slutsky S.G., Revel M. (2020). Rising Stars: Astrocytes as a Therapeutic Target for ALS Disease. Front. Neurosci..

[123] Simons M., Nave K.A. (2015). Oligodendrocytes: Myelination and Axonal Support. Cold Spring Harb. Perspect. Biol..

[124] Stadelmann C., Timmler S., Barrantes-Freer A., Simons M. (2019). Myelin in the Central Nervous System: Structure, Function, and Pathology. Physiol. Rev..

[125] Festa L.K., Grinspan J.B., Jordan-Sciutto K.L. (2024). White matter injury across neurodegenerative disease. Trends Neurosci..

[126] Barton S.K., Gregory J.M., Selvaraj B.T., McDade K., Henstridge C.M., Spires-Jones T.L., James O.G., Mehta A.R., Story D., Burr K. (2021). Dysregulation in Subcellular Localization of Myelin Basic Protein mRNA Does Not Result in Altered Myelination in Amyotrophic Lateral Sclerosis. Front. Neurosci..

[127] Kang S.H., Li Y., Fukaya M., Lorenzini I., Cleveland D.W., Ostrow L.W., Rothstein J.D., Bergles D.E. (2013). Degeneration and impaired regeneration of gray matter oligodendrocytes in amyotrophic lateral sclerosis. Nat. Neurosci..

[128] Philips T., Bento-Abreu A., Nonneman A., Haeck W., Staats K., Geelen V., Hersmus N., Küsters B., Van Den Bosch L., Van Damme P. (2013). Oligodendrocyte dysfunction in the pathogenesis of amyotrophic lateral sclerosis. Brain A J. Neurol..

[129] Pons A.L., Higginbottom A., Cooper-Knock J., Alrafiah A., Alofi E., Kirby J., Shaw P.J., Wood J.D., Highley J.R. (2020). Oligodendrocyte pathology exceeds axonal pathology in white matter in human amyotrophic lateral sclerosis. J. Pathol..

[130] Sadler G.L., Lewis K.N., Narayana V.K., De Souza D.P., Mason J., McLean C., Gonsalvez D.G., Turner B.J., Barton S.K. (2022). Lipid Metabolism Is Dysregulated in the Motor Cortex White Matter in Amyotrophic Lateral Sclerosis. Metabolites.

[131] Sirisi S., Querol-Vilaseca M., Dols-Icardo O., Pegueroles J., Montal V., Muñoz L., Torres S., Ferrer-Raventós P., Iulita M.F., Sánchez-Aced É. (2022). Myelin loss in C9orf72 hexanucleotide expansion carriers. J. Neurosci. Res..

[132] Floriddia E.M., Lourenço T., Zhang S., van Bruggen D., Hilscher M.M., Kukanja P., Dos Santos J.P.G., Altınkök M., Yokota C., Llorens-Bobadilla E. (2020). Distinct oligodendrocyte populations have spatial preference and different responses to spinal cord injury. Nat. Commun..

[133] Zeisel A., Hochgerner H., Lönnerberg P., Johnsson A., Memic F., van der Zwan J., Häring M., Braun E., Borm L.E., La Manno G. (2018). Molecular Architecture of the Mouse Nervous System. Cell.

