# Supplementary material for: Meta-Analysis of Gene Expression in Bulk-Processed Post-Mortem Spinal Cord from ALS Patients and Normal Controls
Source: NeuroSci. 2025 Jul 16;6(3):65. doi: 10.3390/neurosci6030065 (PMC12286074; doi:10.3390/neurosci6030065)
Supplement: Supplementary file 1 [file neurosci-06-00065-s001.zip › neurosci-3659146-supplementary/Supplement.pdf]

Supplemental Figures

Meta-analysis of gene expression in bulk-processed post-mortem spinal cord from ALS patients and normal controls

William R. Swindell[1†]
[1]University of Texas Southwestern Medical Center, Dallas, TX, United States
[†]Corresponding author: William.Swindell@UTSouthwestern.edu

Supplementary Figure Legends

Figure S1. GSE137810 mapping results (*n* = 481 samples). (A) Number of reads per sample prior to quality filtering. (B) Number of reads per sample after quality filtering. (C) Percentage of mapped reads. (D) Percentage of reads mapped uniquely. (E) Percentage of reads mapped to intragenic regions. (F) Percentage of reads mapped to exonic regions. (G) Percentage of reads mapped to ribosomal genes. (H) Percentage of protein-coding genes with detectable expression. Boxplots are shown in the top margin of each figure (boxes: 25th to 75th percentiles; whiskers: 10th to 90th percentiles; open symbols used for samples below the 10th or above the 90th percentiles).

Figure S2. GSE137810 covariates and variable importance (*n* = 481 samples). (A - J) Principal component scatterplots. Each point corresponds to a single sample. Samples are plotted with respect to the first two principal component axes. Symbols are color-coded as indicated (top margin legend). The dashed green line in (A) denotes the linear discriminant function separating ALS and CTL samples with balanced classification accuracy shown (top right). (K) Random forest variable importance scores. Samples were assigned to groups based upon unsupervised hierarchical clustering (Euclidean distance). Random forest was then used to predict group membership based on the variables listed (left margin). The horizontal axis plots importance scores for each variable, defined as the percent out-of-bag misclassification increase when the listed variable is permuted. (G) Likelihood ratio test (LRT) of variable importance. An LRT was used to compare univariate "full" models (including only the listed variable) to intercept-only "reduced" models. LRT p-values were calculated based on the decrease in model deviance (full model vs. reduced model). Analyses were replicated for all protein-coding genes. Boxplots outline the distribution of $\log_{10}$-transformed p-values obtained across genes for each variable (boxes: 25th to 75th percentiles; whiskers: 10th to 90th percentiles). The median $\log_{10}$-transformed p-value is listed (right margin).

Figure S3. Hierarchical cluster analyses. Samples were clustered using the Euclidean distance metric with average linkage (ALS: red font; CTL: black font). (A) GSE137810-N. (B) GSE137810-H. (C) GSE255683. (D) SRP064478. (E) GSE26927. (F) E-MTAB-8638. In part (F), samples with an asterisk (*) were excluded from differential expression testing.

Figure S4. GSE255683 read mapping results (*n* = 20 samples). (A) Number of reads per sample prior to quality filtering. (B) Number of reads per sample after quality filtering. (C) Percentage of mapped reads. (D) Percentage of reads mapped uniquely. (E) Percentage of reads mapped to intragenic regions. (F) Percentage of reads mapped to exonic regions. (G) Percentage of reads mapped to ribosomal genes. (H) Percentage of protein-coding genes with detectable expression. In (A) - (H), the final column indicates the average value across all samples.

Figure S5. SRP064478 read mapping results (*n* = 15 samples). (A) Number of reads per sample prior to quality filtering. (B) Number of reads per sample after quality filtering. (C) Percentage of mapped reads. (D) Percentage of reads mapped uniquely. (E) Percentage of reads mapped to intragenic regions. (F) Percentage of reads mapped to exonic regions. (G) Percentage of reads mapped to ribosomal genes. (H) Percentage of protein-coding genes with detectable expression. In (A) - (H), the final column indicates the average value across all samples.

Figure S6. Microarray signal intensity distributions and number of detected genes (GSE26927). (A) Non-normalized signal intensity. (B) Background-adjusted signal intensity (*). (C) Background-adjusted quantile-normalized signal intensity (**). (D) Number of protein-coding genes with detectable expression in each microarray sample. In (A) - (C), boxplots outline the middle 50% of values among microarray probes (whiskers: 10th to 90th percentiles). In (D), the number of protein-coding genes with detectable expression is shown for each array sample.

Figure S7. Microarray signal intensity distributions and number of detected genes (E-MTAB-8635). (A) Cy3 signal foreground (FG) intensity. (B) Cy3 signal background (BG) intensity. (C) Background-adjusted Cy3 signal (Cy3*). (D) Background-adjusted quantile-normalized Cy3 signal (Cy3**). (E) Background-adjusted quantile-normalized spatially-corrected Cy3 signal (Cy3***). This value represents the final intensity estimate used for differential expression testing. (F) Number of protein-coding genes with detectable expression in each microarray sample. In (A) - (E), boxplots outline the middle 50% of values among microarray probes (whiskers: 10th to 90th percentiles). In (F), the number of protein-coding genes with detectable expression is shown for each array sample. In (A) - (F), sample labels with an asterisk (*) denote those filtered out prior to differential expression testing (due to spatial artifacts).

Figure S8. Microarray pseudoimages (E-MTAB-8635). Heatmaps show background-corrected quantile-normalized Cy-3 signals following z-score normalization (see scale). An asterisk is used (bottom margin label) to denote samples excluded from differential expression testing due to spatial artifacts.

Figure S9. Microarray pseudoimages (E-MTAB-8635). Heatmaps show background-corrected quantile-normalized spatially-corrected Cy-3 signals following z-score normalization (see scale). An asterisk is used (bottom margin label) to denote samples excluded from differential expression testing due to spatial artifacts.

Figure S10. Spatial transcriptomic quality control metrics for human lumbar spinal cord samples (GSE222322, 10x Genomics Visium array). (A) Molecular count distribution across spots. (B) Gene count distribution across spots. (C) Percentage of molecular counts associated with mitochondrial genes. Boxes shown for each sample span the 25th to 75th percentiles of observed values among spots (whiskers: 10th to 90th percentiles). The median value among all spots is represented by the middle line within each box and printed in the upper margin of each figure. Different colors are used for samples obtained from different donors.

Figure S11. Spatial transcriptomic molecular counts for human lumbar spinal cord samples (GSE222322, 10x Genomics Visium array). H&E slides are shown for each sample (upper left) with an enlarged image that is color-coded based on molecular counts across spots (right). The color scale shown for each sample (lower left) spans the middle 90% of counts observed among all spots on the slide.

Figure S12. Spatial transcriptomic gene counts for human lumbar spinal cord samples (GSE222322, 10x Genomics Visium array). H&E slides are shown for each sample (upper left) with an enlarged image that is color-coded based on gene counts across spots (right). The color scale shown for each sample (lower left) spans the middle 90% of counts observed among all spots on the slide.

Figure S13. Spatial transcriptomic mitochondrial molecular count percentage for human lumbar spinal cord samples (GSE222322, 10x Genomics Visium array). H&E slides are shown for each sample (upper left) with an enlarged image that is color-coded based on mitochondrial count percentage across spots (right). The color scale shown for each sample (lower left) spans the middle 90% of mitochondrial percentages observed among all spots on the slide.

Figure S14. Principal component analyses. (A – F) Principal component analyses. Samples from each dataset are plotted with respect to the first two PC axes (ALS: red symbols; CTL: black symbols). The linear discriminant function is shown (dashed green line) with balanced classification accuracy (green font). (G - L). Principal component radial plots. Average scores by group are shown with respect to the first 12 PC axes (ALS: red line; CTL: black line; dashed lines: ± 1 standard error; open symbols: multivariate mean). An asterisk (*) denotes PC axes with a significant difference between ALS and CTL samples ($P < 0.05$, two-sample two-tailed t-test).

Figure S15. Analysis of differential expression p-values (ALS vs. CTL). (A - F) Quantile-quantile plots. Differential expression T-statistic quantiles are compared to theoretical quantiles of the Student's t distribution. Deviation for a straight line (null) is indicative of a higher percentage of differentially expressed genes. (G - L) Raw p-value histograms. P-values derived from differential expression testing are shown in each histogram. (M - R) Raw p-value empirical cumulative distribution functions (CDFs). Each plot shows the empirical CDF of raw p-values obtained from each differential expression analysis.

Figure S16. Differential expression volcano and MA plots. (A – F) Volcano plots. The -log10-transformed p-value (vertical axis) is plotted with respect to the estimated standardized median difference (SMD) (horizontal axis). The number of differentially expressed genes is indicated (top margin). (G – L) MA plots. SMD (vertical axis) is plotted with respect to the average expression level of genes, as measured by FPKM (RNA-seq) or normalized signal intensity (microarray). The local polynomial regression estimate (loess) is shown (green line).

Figure S17. Differential expression associations with gene length and GC content. (A - F) Gene length analysis. The percentage of DEGs is shown (vertical axis) across groups of genes stratified by gene length (horizontal axis). (G - L) GC content analysis. The percentage of DEGs is shown (vertical axis) across groups of genes stratified by GC content (horizontal axis). In (A) - (L), an asterisk is shown at the top of each bar if the total percentage of DEGs (increased + decreased) in that group is larger than expected by chance ($P < 0.05$, Fisher's exact test). Similarly, the percentage within each bar is shown in yellow font if the percentage of increased or decreased DEGs, respectively, is larger than expected by chance ($P < 0.05$, Fisher's exact test).

Figure S18. Differential expression meta-analyses (ALS vs. CTL). (A) Raw p-value histogram. The meta-analysis p-value histogram is shown. (B) Raw p-value empirical cumulative distribution function (CDF). The empirical CDF of raw p-values obtained from the differential expression meta-analysis is shown. (C) Volcano plot. The -log10-transformed p-value (vertical axis) is plotted with respect to the estimated standardized median difference (SMD) (horizontal axis). The number of differentially expressed genes is indicated (top margin). (D) MA plot. The SMD (vertical axis) is plotted with respect to the average expression level of genes (normalized to the [0, 1] interval). The green line represents the local polynomial regression estimate (loess). (E) Gene length analysis. The percentage of DEGs is shown (vertical axis) across groups of genes stratified by gene length (horizontal axis). (F) GC content analysis. The percentage of DEGs is shown (vertical axis) across groups of genes stratified by GC content (horizontal axis). In (E) and (F), an asterisk is shown at the top of each bar if the total percentage of DEGs (increased + decreased) in that group is larger than expected by chance ($P < 0.05$, Fisher's exact test). Similarly, the percentage within each bar is shown in yellow font if the percentage of increased or decreased DEGs, respectively, is larger than expected by chance ($P < 0.05$, Fisher's exact test).

Figure S19. *SLC37A2* expression summary. Violin plots are shown for (A) all datasets merged, (B) GSE137810-N, (C) GSE137810-H, (D) GSE255683, (E) SRP064478, (F) GSE26927 and (G) E-MTAB-8635. In (A) - (G), the total number of CTL and ALS samples is indicated (bottom margin). Plots show expression values for individual samples after applying a z-score transformation to normalized expression intensities on a $\log_2$ scale.

Figure S20. *NDRG1* expression summary. Violin plots are shown for (A) all datasets merged, (B) GSE137810-N, (C) GSE137810-H, (D) GSE255683, (E) SRP064478, (F) GSE26927 and (G) E-MTAB-8635. In (A) - (G), the total number of CTL and ALS samples is indicated (bottom margin). Plots show expression values for individual samples after applying a z-score transformation to normalized expression intensities on a $\log_2$ scale.

Figure S21. GO BP term cluster analysis. (A) Top 30 GO BP terms enriched with respect to ALS-increased DEGs (SMD > 0.80, FDR < 0.05). (B) Top 30 GO BP terms enriched with respect to ALS-decreased DEGs (SMD < -0.80, FDR < 0.05). In (A) and (B), top-ranking GO BP terms are hierarchically clustered, with distance between terms defined based upon the percentage of overlap between ALS-increased or ALS-decreased genes linked to any two terms. Term enrichment was assessed based upon the p-value obtained from a conditional hypergeometric test (R package: GOstats, function: hyperGTest). The $-\log_{10}$-transformed p-value is indicated for each term (see legend, bottom). The font of each GO BP term shown is color-coded based upon its branch within the dendrogram. Example ALS-increased or ALS-decreased DEGs are given in parentheses for each term.

Figure S22. Comparison of meta-analysis DEGs to those previously identified from NYGC ALS Consortium data (Humphrey et al. 2023, Nat Neurosci 26:150-162). (A, B) Venn diagrams. The overlap between (A) ALS-increased and (B) ALS-decreased DEGs from each analysis (p-value: Fisher's exact test). (C, D) Rank-ordered meta-analysis SMD estimates for (C) ALS-increased or (D) ALS-decreased DEGs identified by Humphrey et al. (2023) (red: SMD > 0; blue: SMD < 0; percentage of genes within each group is shown, see legend). Pie charts show

the overall proportion of genes with SMD > 0 (red) and SMD < 0 (blue) (outer circle: all genes included in analysis; inner circle: genes altered in the Humphrey et al. (2023) analysis; p-value: comparison of inner/outer chart proportions, Fisher's exact test). In (A) - (D), DEGs from Humphrey et al. (2023) were significantly altered (FDR < 0.05) in the same direction (FC > 0 or FC < 0) across all 3 spinal cord sections. (E, F) Top genes most strongly (E) ALS-increased or (F) ALS-decreased in both analyses. Genes are ordered based on the meta-analysis SMD estimate. The average FC reported by Humphrey et al. (2023) is plotted with symbols proportional to the average -log10-transformed p-value (see scale). Genes shown in red or blue font met DEG criteria for both analyses (meta-analysis: FDR < 0.05 with SMD > 0.80 or SMD < -0.80; Humphrey: FDR < 0.05 with FC > 1 or FC < 1 in each cord section).

Figure S23. Meta-analysis comparison of ALS gene dysregulation in human spinal cord (bulk tissue vs. LCM-MN). The current bulk tissue meta-analysis was compared to a prior meta-analysis focused on laser capture microdissected motor neurons (LCM-MN) (Swindell 2024, Front Genet 15:1385114). (A, B) Venn diagrams. The overlap between (A) ALS-increased and (B) ALS-decreased DEGs from each meta-analysis (p-value: Fisher's exact test). (C, D) Rank-ordered bulk-SMD estimates for genes (C) increased or (D) decreased in the LCM-MN meta-analysis (red: SMD > 0; blue: SMD < 0; percentage of genes within each group is shown, see legend). Pie charts show the overall proportion of genes with SMD > 0 (red) and SMD < 0 (blue) (outer circle: all genes included in analysis; inner circle: genes altered in LCM-MN meta-analysis; p-value: comparison of inner/outer chart proportions, Fisher's exact test). (E, F) Top genes most strongly (E) ALS-increased or (F) ALS-decreased in bulk spinal cord and LCM-MN meta-analyses. Genes are ordered based on the bulk tissue SMD meta-estimate. The meta-estimate obtained from the LCM-MN analysis is plotted with symbols proportional to the -log10-transformed p-value (see scale). Genes shown in red font were significantly increased in both meta-analyses (SMD > 0.80, FDR < 0.10). Genes shown in blue font were significantly decreased in both meta-analyses (SMD < -0.80, FDR < 0.10).

Figure S24. Overlap between DEGs and proteins dysregulated in ALS spinal cord. Transcriptome meta-analysis DEGs are compared to genes associated with proteins having significantly altered abundance in ALS patient spinal cord (153 ALS-increased proteins, 139 ALS-decreased proteins; FDR = 0.05; see Oeckl et al. 2020, Acta Neuropathologica 139:119-134). (A, B) Venn diagrams. Part (A) shows overlap between ALS-increased DEGs and genes associated with ALS-increased proteins (p-value: Fisher's exact test). Part (B) shows overlap between ALS-decreased DEGs and genes associated with ALS-decreased proteins (p-value: Fisher's exact test). (C, D) Rank-ordered SMD estimates for genes associated with proteins (C) increased in ALS spinal cord or (D) decreased in ALS spinal cord (red: SMD > 0; blue: SMD < 0; percentage of genes within each group is shown, see legend). Pie charts show the overall proportion of genes with SMD > 0 (red) or SMD < 0 (blue) (outer circle: all genes/proteins included in analysis; inner circle: genes linked to ALS-dysregulated proteins; p-value: comparison of inner/outer chart proportions, Fisher's exact test). (E, F) Top genes/proteins most strongly (E) ALS-increased or (F) ALS-decreased in both transcriptome/proteome studies (red font: increased in transcriptome meta-analysis, SMD > 0.80, FDR < 0.05; blue font: decreased in transcriptome meta-analysis, SMD < -0.80, FDR < 0.05). The SMD meta-estimate is shown for each gene and the fold-change estimate (ALS/CTL) for the corresponding protein is also plotted with symbols proportional to the -log10-transformed p-value (see scale).

Figure S25. Overlap between DEGs and genes dysregulated in whole blood from ALS patients. Transcriptome meta-analysis DEGs are compared to genes with significantly altered expression in whole blood from ALS patients (752 ALS-increased genes, 764 ALS-decreased genes, FDR < 0.10 with greater than 10% expression change; see Swindell et al. 2019, J Transl Med 17:170). (A, B) Venn diagrams. Part (A) shows overlap between ALS-increased DEGs and genes associated with increased expression in ALS patient blood (p-value: Fisher's exact test). Part (B) shows overlap between ALS-decreased DEGs and genes with decreased expression in ALS patient blood (p-value: Fisher's exact test). (C, D) Rank-ordered SMD estimates for genes associated with genes (C) increased in ALS blood or (D) decreased in ALS blood (red: SMD > 0; blue: SMD < 0; percentage of genes within each group is shown, see legend). Pie charts show the overall proportion of genes with SMD > 0 (red) or SMD < 0 (blue) (outer circle: all genes/proteins included in analysis; inner circle: genes dysregulated in ALS patient blood; p-value: comparison of inner/outer chart proportions, Fisher's exact test). (E, F) Top genes most strongly (E) ALS-increased or (F) ALS-decreased in both spinal cord and blood (red font: increased in transcriptome meta-analysis, SMD > 0.80, FDR < 0.05; blue font: decreased in transcriptome meta-analysis, SMD < -0.80, FDR < 0.05). The SMD meta-estimate is shown for each gene and the fold-change estimate (ALS/CTL) in blood samples is also plotted with symbols proportional to the -log10-transformed p-value (see scale).

Figure S26. Overlap between DEGs and genes near ALS GWAS loci. (A) Manhattan plot. Genes overlying and near GWAS loci are plotted using different symbols (right margin). An asterisk symbol (*) is used to denote genes having detectable expression in spinal cord samples and included in the differential expression meta-analysis. (B) SMD estimates of ALS-GWAS genes. Estimates are ordered (red: ALS-increased; blue: ALS-decreased). The percentage of ALS-increased and ALS-decreased genes is shown in the bottom right legend. Pie charts (top left) represent the proportion of ALS-GWAS genes with ALS-increased (red) or ALS-decreased (blue) expression (inner chart: ALS-GWAS genes; outer chart: all genes included in the meta-analysis). The p-value tests for a difference between the inner and outer pie chart proportions (Fisher's exact test). (C - E) Venn diagrams. Overlap is shown between DEGs and ALS-GWAS genes. Results are shown for (C) all DEGs (increased + decreased) (D) ALS-increased DEGs and (E) ALS-decreased DEGs. (F) Overlap between DEGs and ALS-GWAS genes. Percent overlap is shown (vertical axis) between increased (red) or decreased (blue) DEGs and ALS-GWAS genes defined at different genomic distances from ALS-associated loci (horizontal axis). Overlap is also shown with respect to non-DEGs (black line). (G - I) Average distance between DEGs and nearest ALS-associated locus. The average distance (arrow) was calculated with respect to (G) all DEGs, (H) ALS-increased DEGs and (I) ALS-decreased DEGs. Background distributions represent the mean distance obtained by randomly sampling the same number of genes from those included in the meta-analysis (1000 simulation trials). (J) Gene Ontology (GO) biological process (BP) terms. (K) GO cell component (CC) terms. (L) KEGG pathway terms. (M) Reactome pathways. In (J) - (M), annotations significantly enriched among ALS-GWAS genes were identified (P < 0.05) and then evaluated for enrichment with respect to ALS-increased DEGs (horizontal axis) or ALS-decreased DEGs (vertical axis) (Fisher's exact test). The fraction of annotations also demonstrating enrichment with respect to ALS-increased (red) and ALS-decreased (blue) DEGs is shown (P < 0.05). For both ALS-increased and -decreased DEGs, this number was compared to that obtained by randomly sampling the same number of genes and

determining the number of annotations having significant enrichment (P < 0.05) with respect to each gene sample (Fisher's exact test; 1000 simulation trials).

Figure S27. ALS-increased DEG comparison to ALS-increased microglia genes from motor/premotor cortical gray matter (snRNA-seq study by Limone et al. 2024, Nature Aging 4:984-997). (A) Venn diagram. Overlap is shown between ALS-increased DEGs (spinal cord meta-analysis) and ALS-increased microglia genes (Limone et al. 2024). The p-value (bottom) was generated using Fisher's exact test. (B) Rank-ordered bulk-SMD estimates for ALS-increased microglia genes (Limone et al. 2024) (red: SMD > 0; blue: SMD < 0; percentage of genes within each group is shown, see legend). Pie charts show the overall proportion of genes with SMD > 0 (red) and SMD < 0 (blue) (outer circle: all meta-analysis genes; inner circle: ALS-increased microglia genes (Limone et al. 2024); p-value: comparison of inner/outer chart proportions, Fisher's exact test). (C) SMD estimates for ALS-increased microglia genes (Limone et al. 2024). Gene labels with magenta font are expressed more highly by microglia than any other spinal cord cell type (based on snRNA-seq analysis of normal spinal cord, GSE222322).

Figure S28. Oligodendrocyte (OD) phenotypes. (A) OD phenotypes and their signature genes. SMD estimates are shown for signature genes linked to each OD phenotype (left margin). The average SMD estimate among signature genes is listed (right margin). OD phenotypes and signature genes are based upon Table 2 from Valihrach et al. 2022 (Front Cell Neurosci 16:1025012). (B, D, F, H) Venn diagrams. Overlap is shown between meta analysis DEGs and genes linked to the indicated OD phenotype. P-values (bottom) were generated using Fisher's exact test. (C, E) ALS-decreased genes linked to the (C) Marques-MOL5 and (E) Sadick-Int3 phenotypes. (G, I) ALS-increased genes linked to the (G) Jakel-ImOLs and (I) Sadick-Int6 phenoytpes. In (C), (E), (G) and (I), gene labels with magenta font are expressed more highly in ODs than any other spinal cord cell type (based on snRNA-seq data, GSE222322). Bars denote meta analysis SMD estimates (left axis).

Figure S29. Regional enrichment of ALS-increased DEG expression in normal human spinal cord (GSE222322, 10x Genomics Visium array). Expression of ALS-increased DEGs was compared to all other expressed genes at each spot on each slide (Wilcoxon rank sum test). P-values from this comparison were -log10-transformed and the color scale denotes only spots for which expression of ALS-increased DEGs was higher than other genes (P < 0.05). The color scale for each sample shows -log10-transformed p-values ranging from 1.30 (P = 0.05) up to the 98% percentile value observed among all spots on each slide.

Figure S30. Regional enrichment of ALS-decreased DEG expression in normal human spinal cord (GSE222322, 10x Genomics Visium array). Expression of ALS-decreased DEGs was compared to all other expressed genes at each spot on each slide (Wilcoxon rank sum test). P-values from this comparison were -log10-transformed and the color scale denotes only spots for which expression of ALS-decreased DEGs was higher than other genes (P < 0.05). The color scale for each sample shows -log10-transformed p-values ranging from 1.30 (P = 0.05) up to the 98% percentile value observed among all spots on each slide.

Figure S31. ALS-decreased DEGs with high spatial heterogeneity in normal human spinal cord (GSE222322). (A) Moran's I statistic. The heatmap shows ALS-decreased DEGs with the

highest average Moran's I statistic across 20 tissue sections (10x Genomics Visium array). The average Moran's I statistic is listed in parentheses for each gene (left margin). The top 3 samples with the highest Moran’s I statistic for each gene are indicated in each row. (B) *STMN1* expression (sample D-43-10). (C) *KLC1* expression (sample D-43-10). (D) *DHCR24* expression (sample D-43-9). (E) *EDIL3* expression (sample C-47-8). (F) *RAPGEF5* expression (sample C-47-8). In (B) - (F), the raw H&E image is shown (upper left) alongside the same image overlaid with spots color-coded based upon gene expression (see scale). Colors indicate expression of the gene based upon SCT-normalized expression values scaled to the [0, 100] interval. The word cloud (bottom left) indicates average expression of the gene among spinal cord cell types, with larger font sizes used to denote cell types having relatively higher expression of the indicated gene.

Figure S32. ALS-increased DEGs with high spatial heterogeneity in ALS patient spinal cord sections (Maniatis et al. 2019, Science 364: 89-93). (A) Moran's I statistic. The heatmap shows ALS-increased DEGs with the highest average Moran's I statistic across a filtered subset of 50 tissue sections (selected from 80 total sections). Tissue sections from a spinal cord region associated with disease onset are indicated (magenta labels, bottom margin). The average Moran's I statistic is listed in parentheses for each gene (left margin). The top 3 samples with highest Moran’s I statistic for each gene are indicated in each row. (B - E) Average expression of genes by anatomic region. Images show average SCT-normalized and scaled expression by region including all spots from 80 tissue sections. (F) *APOE* expression (sample L8CN7-D1). (G) *GPNMB* expression (sample L8CN152-C2). (H) *APOC1* expression (sample L8CN151-D2). In (F) - (H), the raw H&E image is shown (left) along with the same image color-coded by anatomic region (middle) or gene expression (right). Gene expression is color-coded based upon SCT-normalized expression values scaled to the [0, 100] interval. Word clouds (bottom left) indicate average expression of the indicated gene among spinal cord cell types (GSE190442), with larger font sizes used to denote cell types having relatively higher expression of the gene.

Figure S33. ALS-decreased DEGs and their regional expression in ALS patient cervical/lumbar cord segments (Maniatis et al. 2019, Science 364: 89-93). (A) Cluster analysis. The heatmap shows average expression of ALS-decreased DEGs across 11 spinal cord regions. DEGs have been hierarchically clustered using average linkage and the Euclidean distance. (B) Top-ranked ALS-decreased DEGs. The heatmap shows top ALS-decreased DEGs and their average expression across 11 spinal cord regions. The heatmap color corresponds to average expression and circles indicate the percentage of regional spots with detectable expression. (C) Percentage of DEGs assigned to each spinal cord region. ALS-decreased DEGs were assigned to the region for which average expression was highest. (D) Expression level of DEGs versus non-DEGs by region. (E) Percentage of regional spots with detectable expression among DEGs versus non-DEGs. In (D) and (E), boxes outline the middle 50% of values (whiskers: 10th to 90th percentiles). Clear boxes (background) correspond to non-DEGs whereas colored boxes correspond to DEGs. Filled triangles (top margin) denote cases in which DEG expression is significantly greater than non-DEGs (up-triangles) or significantly less than non-DEGs (down-triangles) (Wilcoxon rank sum test, FDR < 0.05).

Figure S34. ALS-decreased DEGs with high spatial heterogeneity in ALS patient spinal cord sections (Maniatis et al. 2019, Science 364: 89-93). (A) Moran's I statistic. The heatmap

shows ALS-increased DEGs with the highest average Moran's I statistic across a filtered subset of 50 tissue sections (selected from 80 total sections). Tissue sections from a spinal cord region associated with disease onset are indicated (magenta labels, bottom margin). The average Moran's I statistic is listed in parentheses for each gene (left margin). The top 3 samples with highest Moran's I statistic for each gene are indicated in each row. (B - E) Average expression of genes by anatomic region. Images show average SCT-normalized and scaled expression by region including all spots from 80 tissue sections. (F) *SELENOP* (sample L8CN6-D1). (G) *KLC1* expression (sample L8CN6-C1). (H) *EDIL3* expression (sample L8CN12-D1). In (F) - (H), the raw H&E image is shown (left) along with the same image color-coded by anatomic region (middle) or gene expression (right). Gene expression is color-coded based upon SCT-normalized expression values scaled to the [0, 100] interval. Word clouds (bottom left) indicate average expression of the indicated gene among spinal cord cell types (GSE190442), with larger font sizes used to denote cell types having relatively higher expression of the gene.

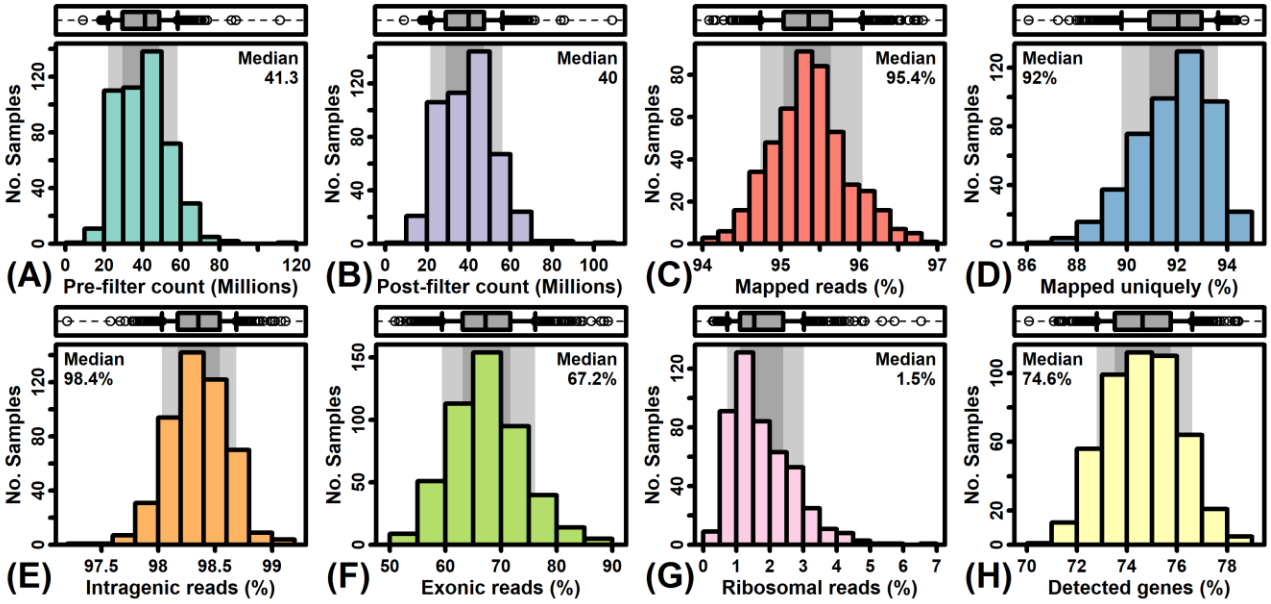

Figure S1. GSE137810 mapping results (*n* = 481 samples).

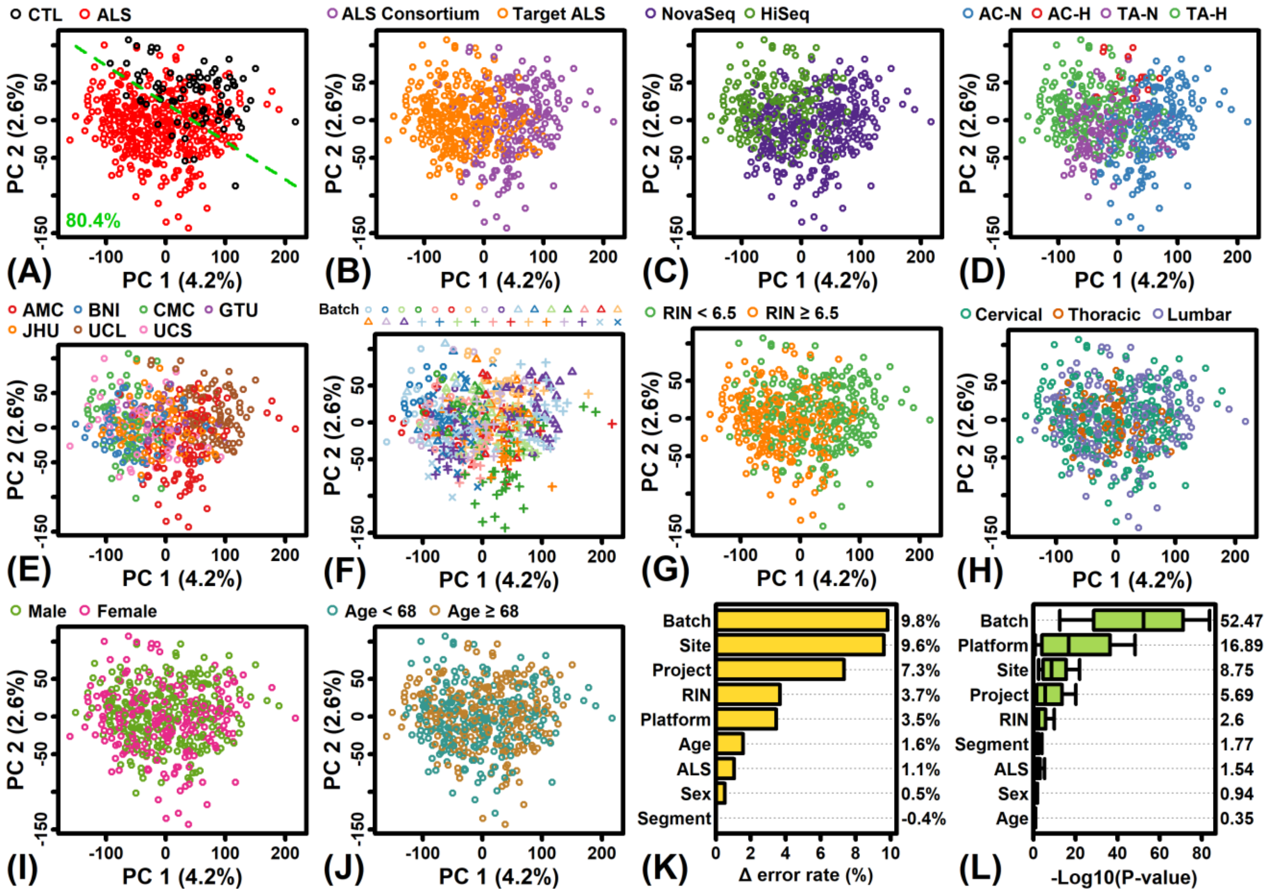

Figure S2. GSE137810 covariates and variable importance (*n* = 481 samples).

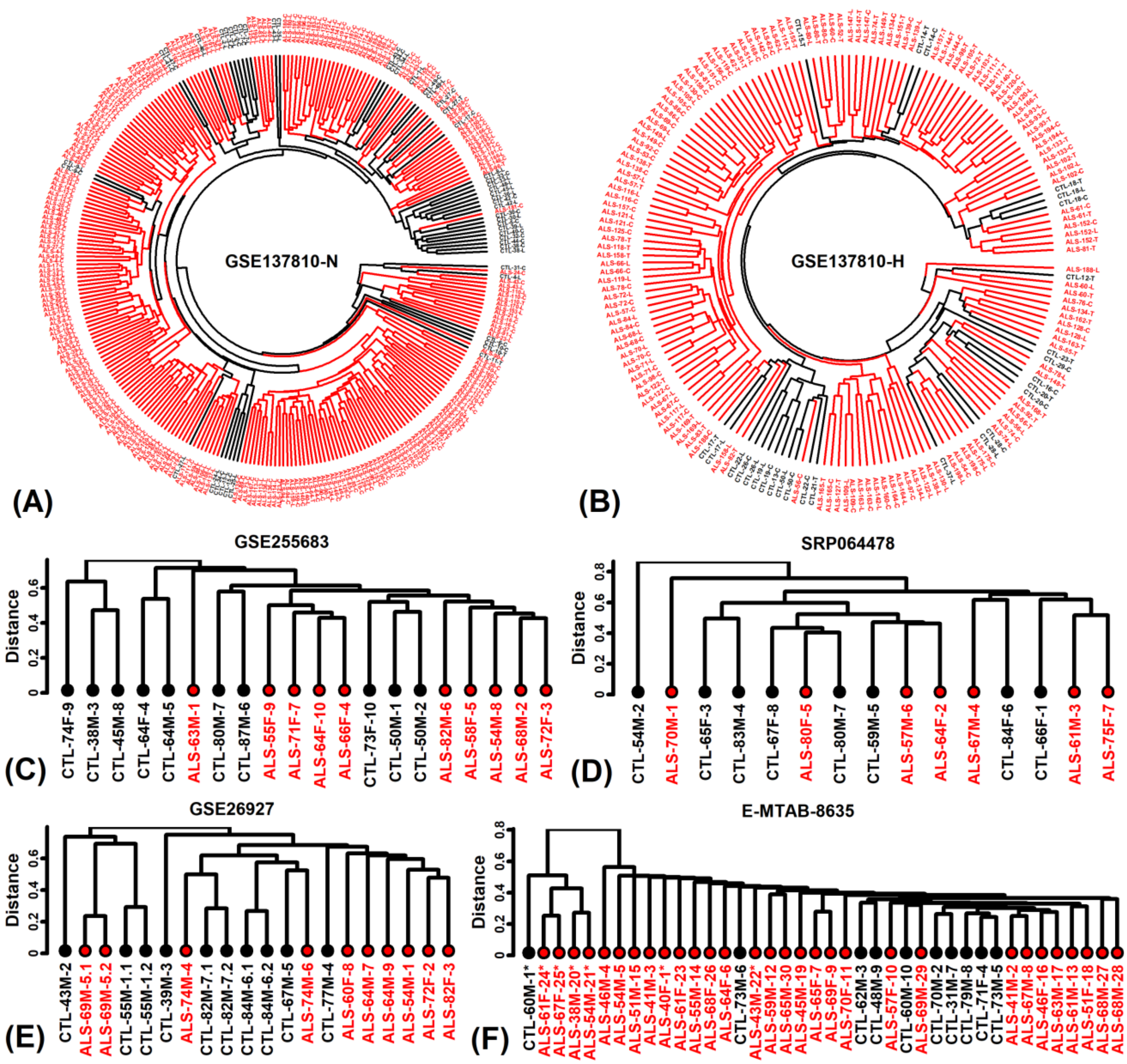

Figure S3. Hierarchical cluster analyses.

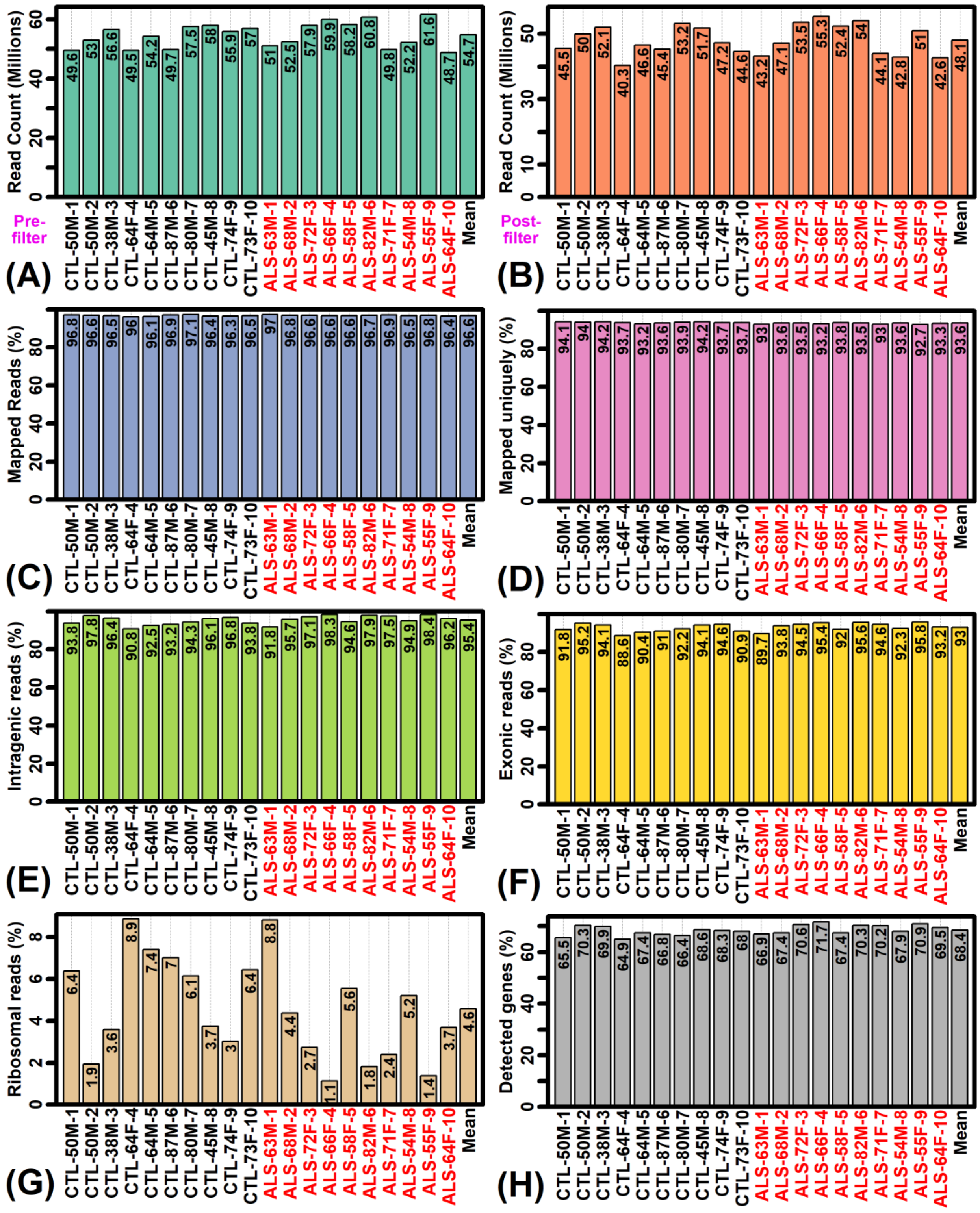

Figure S4. GSE255683 read mapping results (*n* = 20 samples).

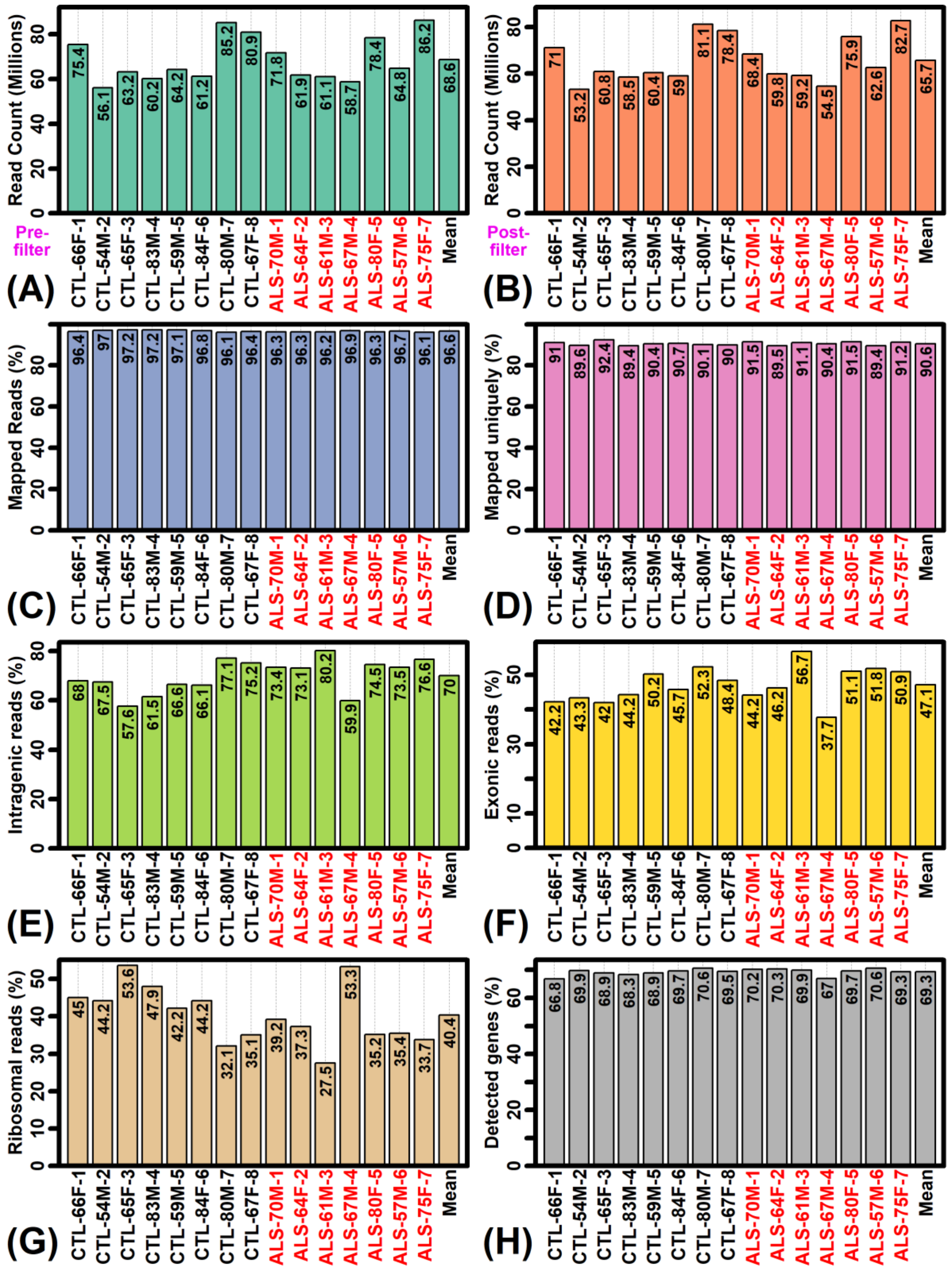

Figure S5. SRP064478 read mapping results (*n* = 15 samples).

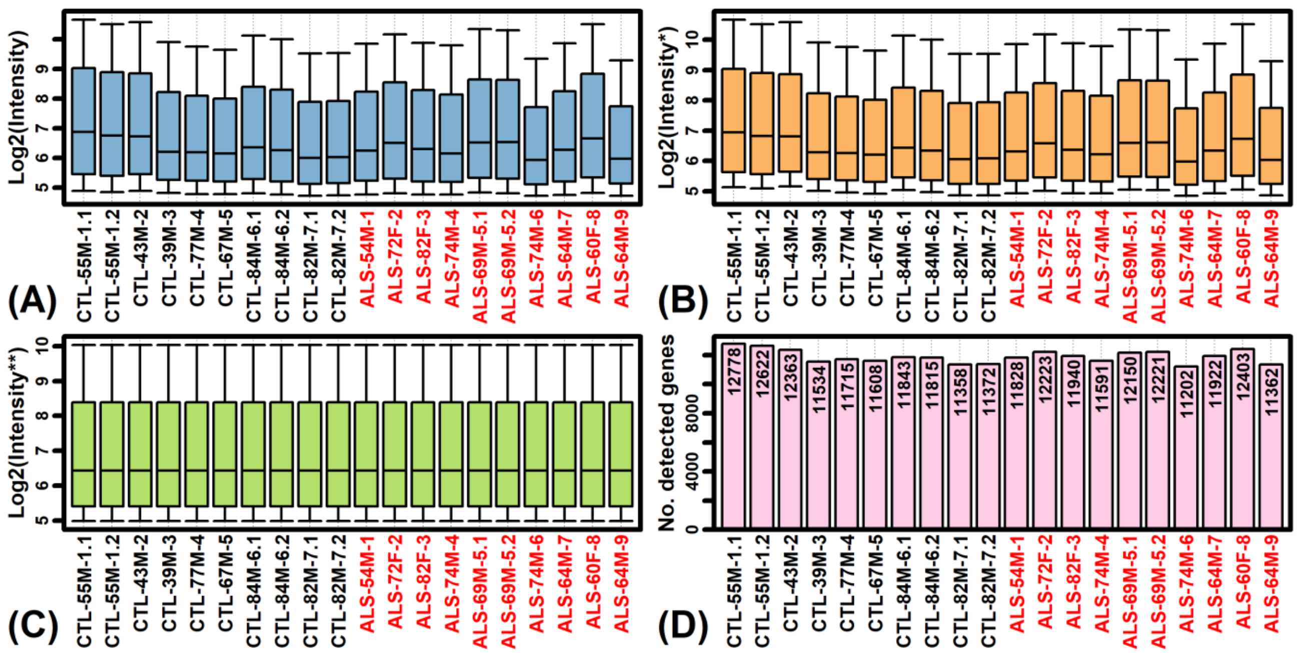

Figure S6. Microarray signal intensity distributions and number of detected genes (GSE26927).

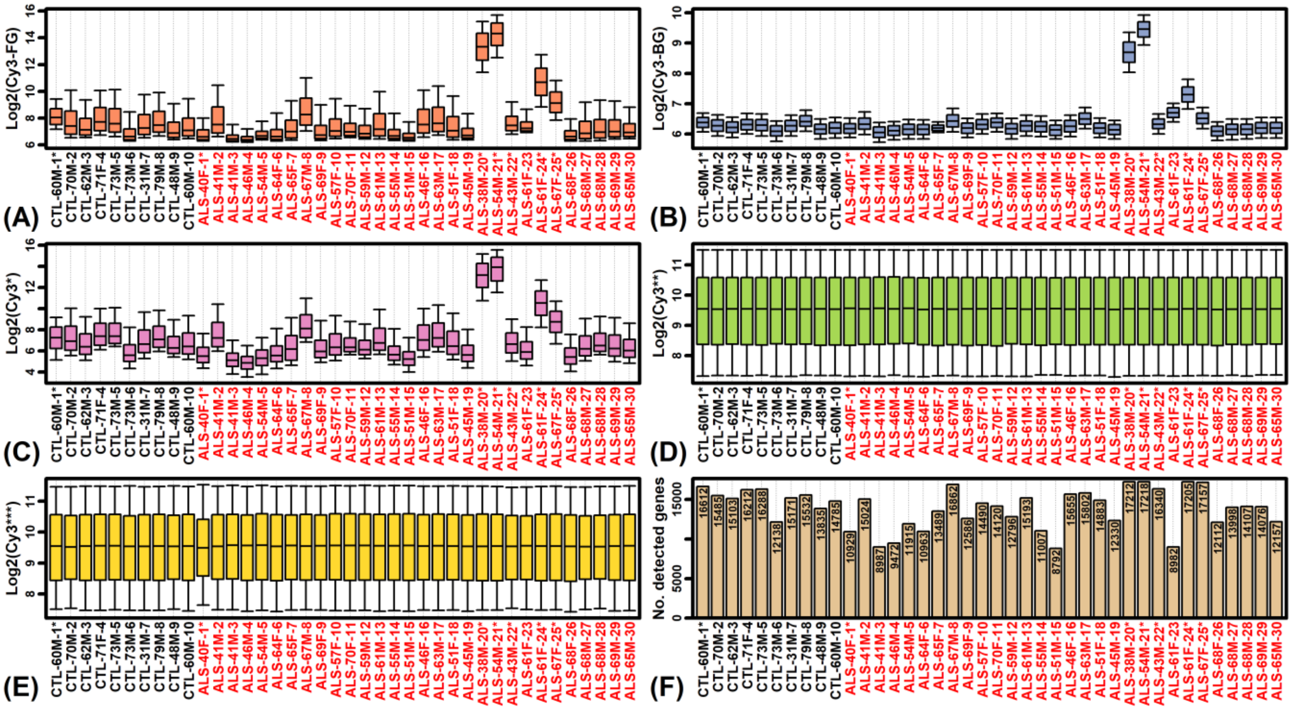

Figure S7. Microarray signal intensity distributions and number of detected genes (E-MTAB-8635).

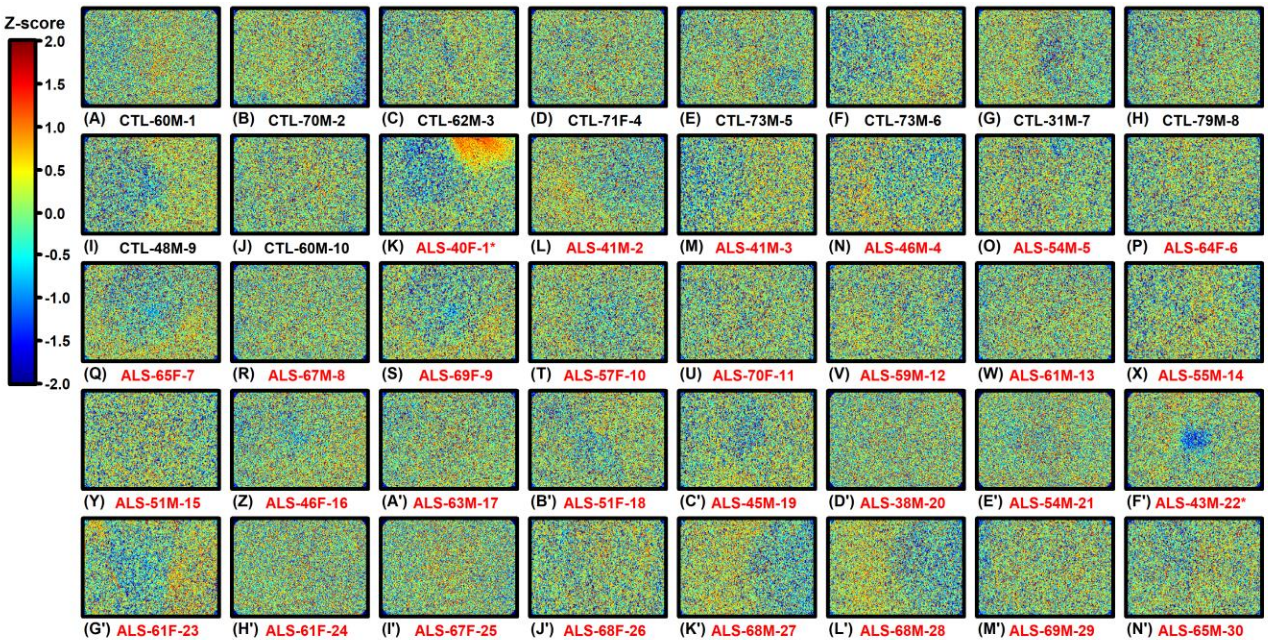

Figure S8. Microarray pseudoimages (E-MTAB-8635).

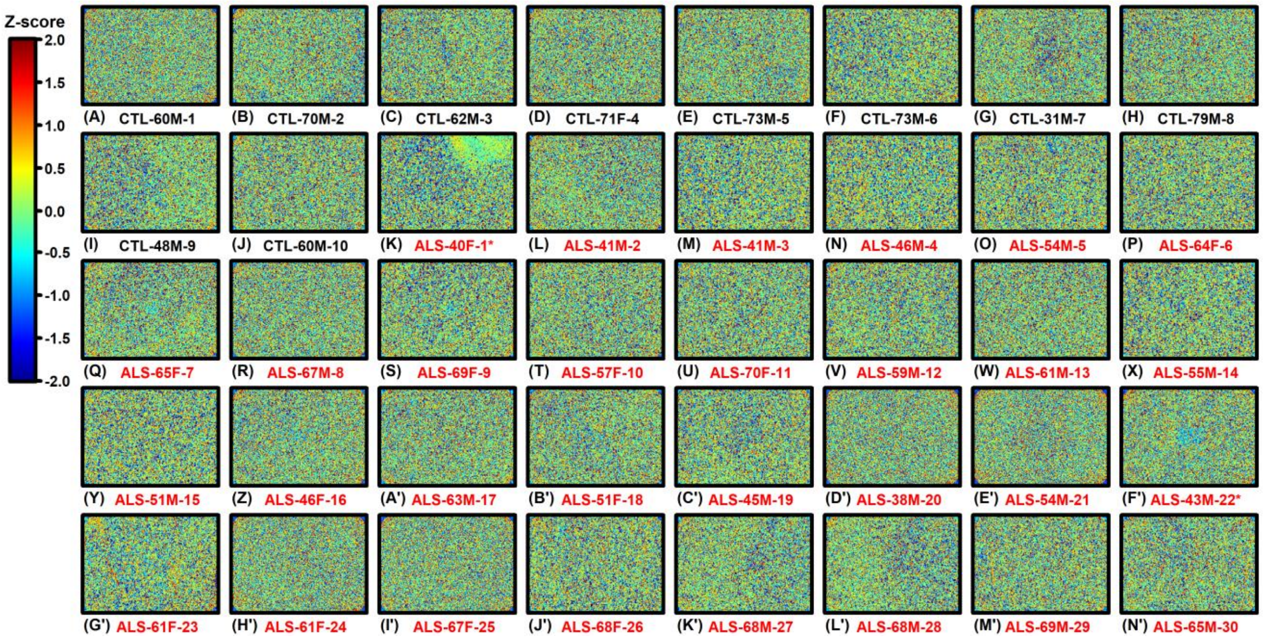

Figure S9. Microarray pseudoimages (E-MTAB-8635).

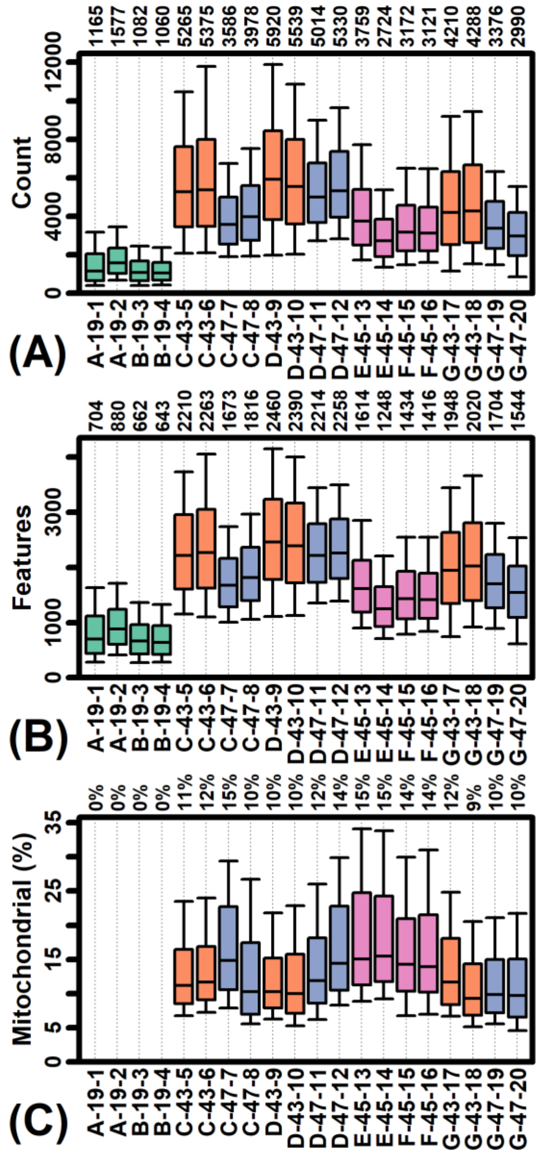

Figure S10. Spatial transcriptomic quality control metrics for human lumbar spinal cord samples (GSE222322, 10x Genomics Visium array).

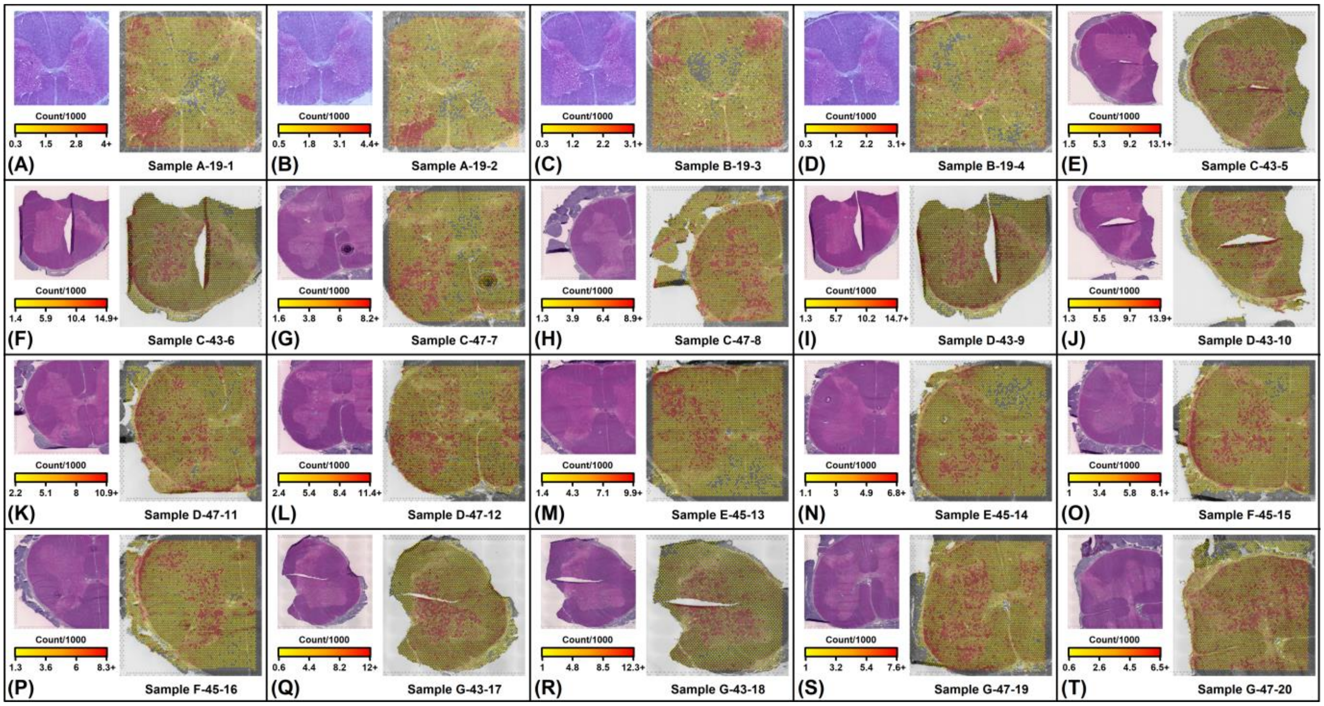

Figure S11. Spatial transcriptomic molecular counts for human lumbar spinal cord samples (GSE222322, 10x Genomics Visium array).

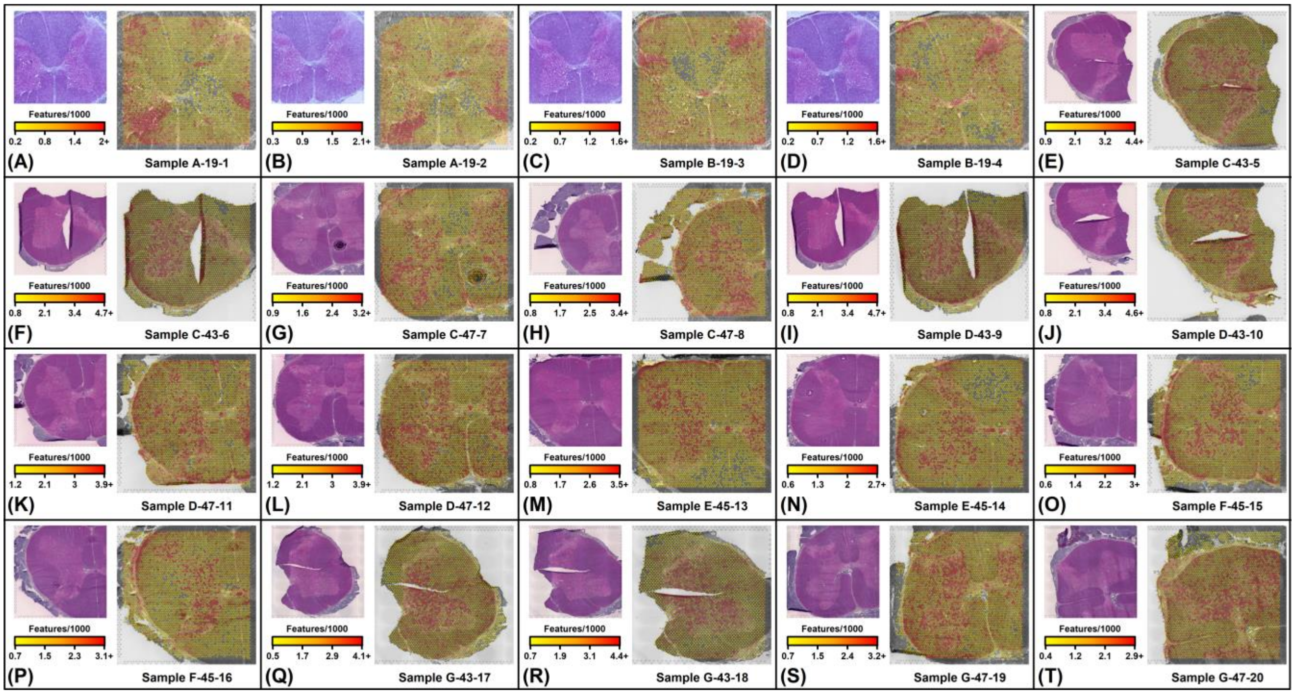

Figure S12. Spatial transcriptomic gene counts for human lumbar spinal cord samples (GSE222322, 10x Genomics Visium array).

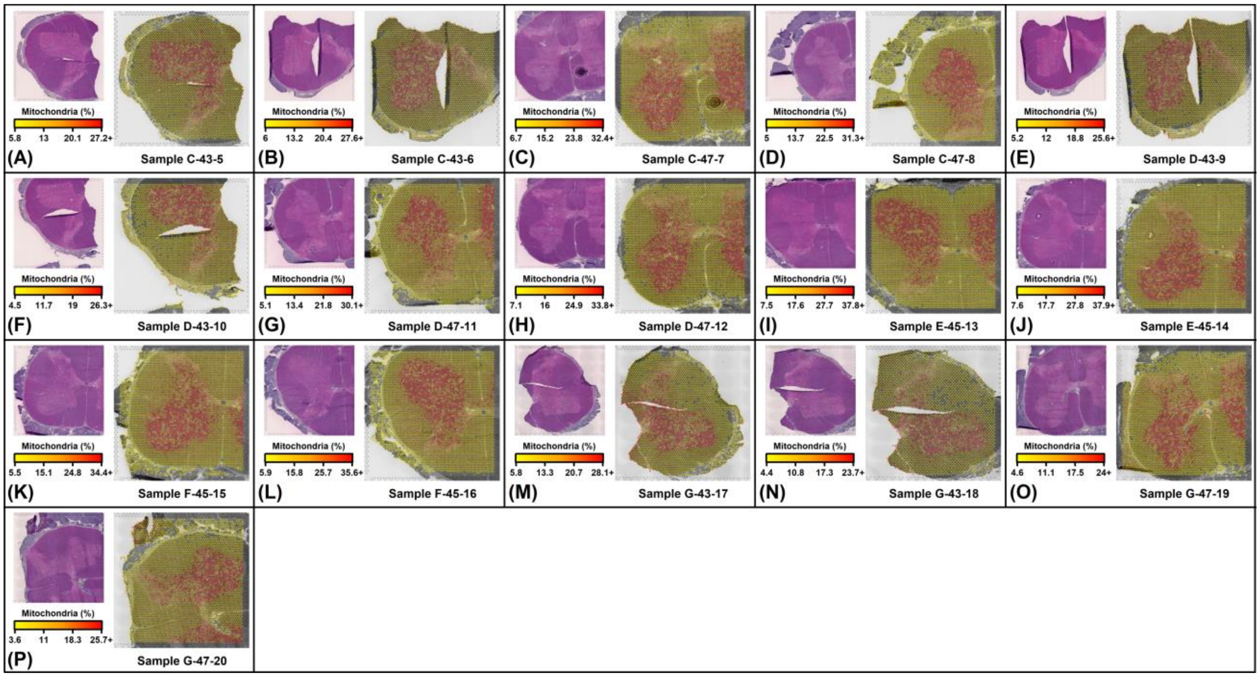

Figure S13. Spatial transcriptomic mitochondrial molecular count percentage for human lumbar spinal cord samples (GSE222322, 10x Genomics Visium array).

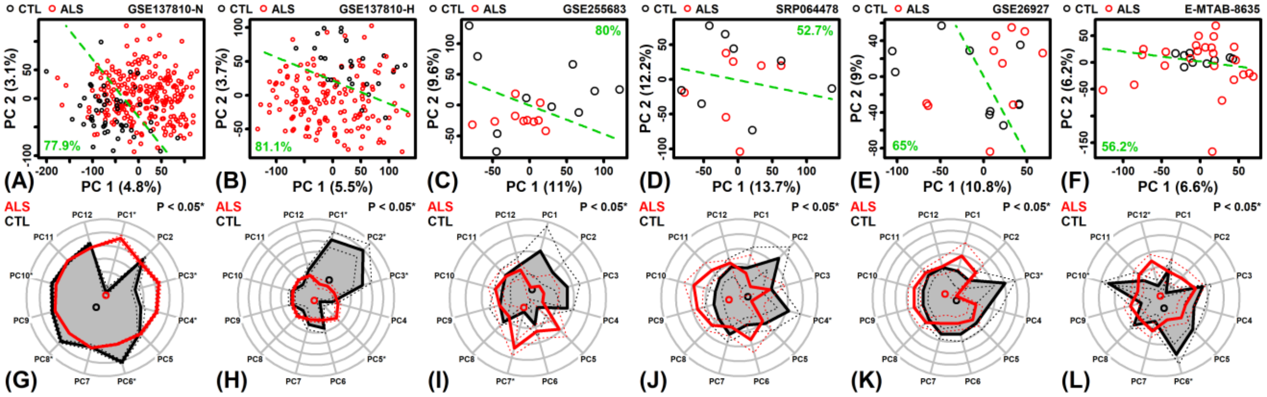

Figure S14. Principal component analyses.

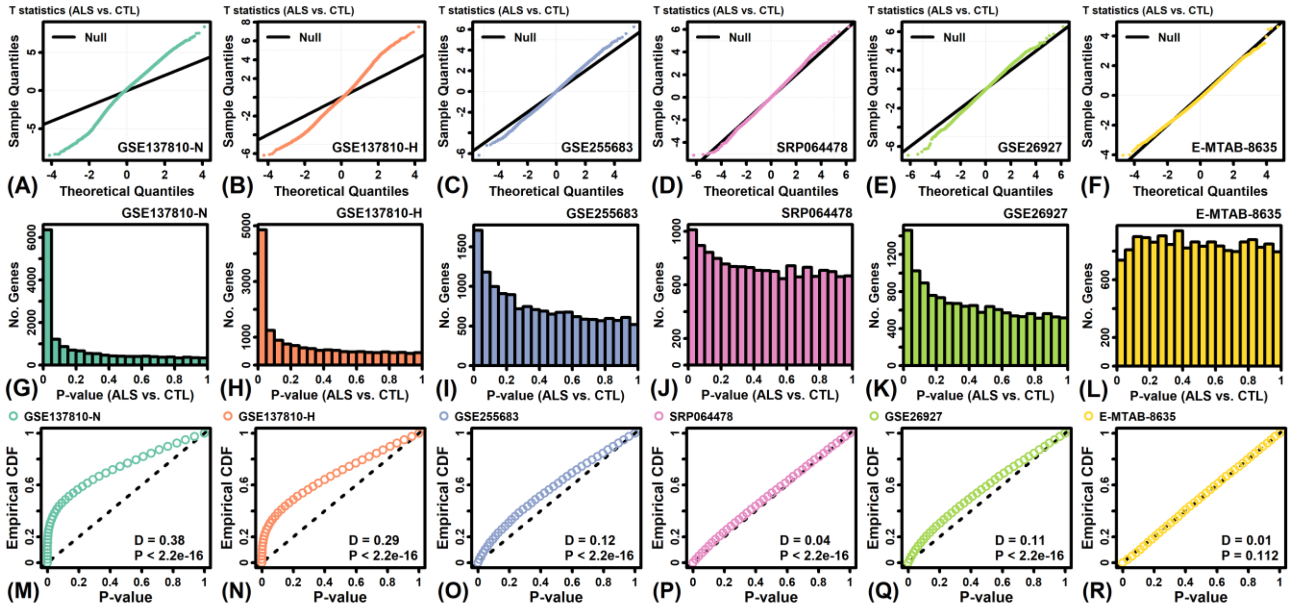

Figure S15. Analysis of differential expression p-values (ALS vs. CTL).

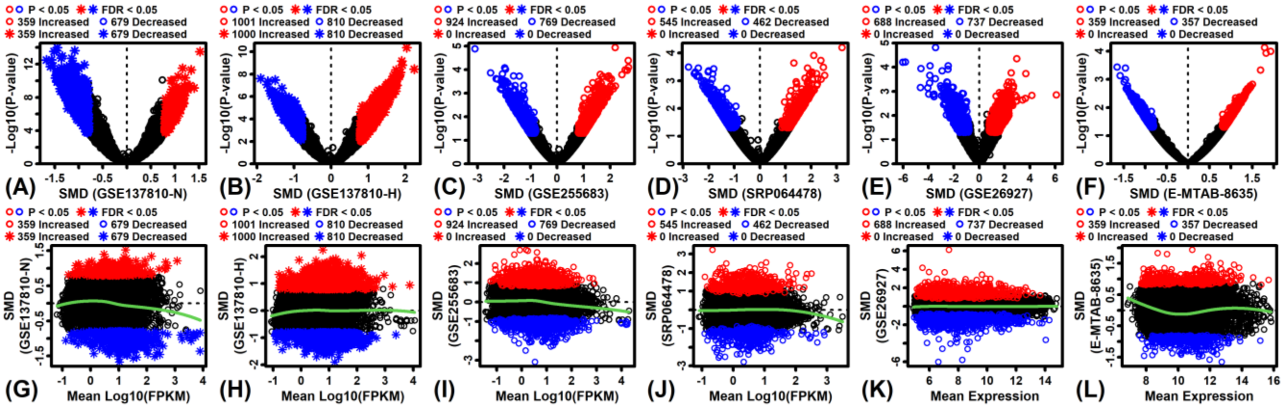

Figure S16. Differential expression volcano and MA plots.

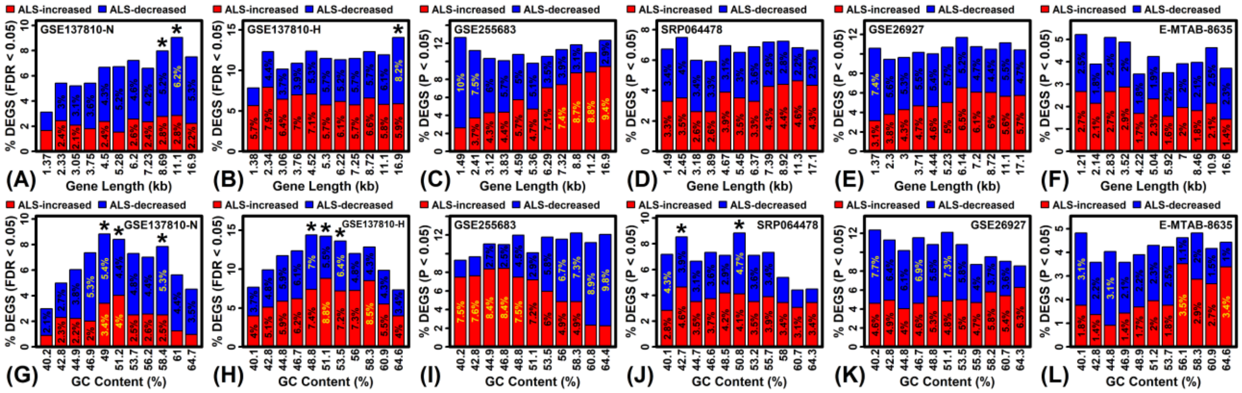

Figure S17. Differential expression associations with gene length and GC content.

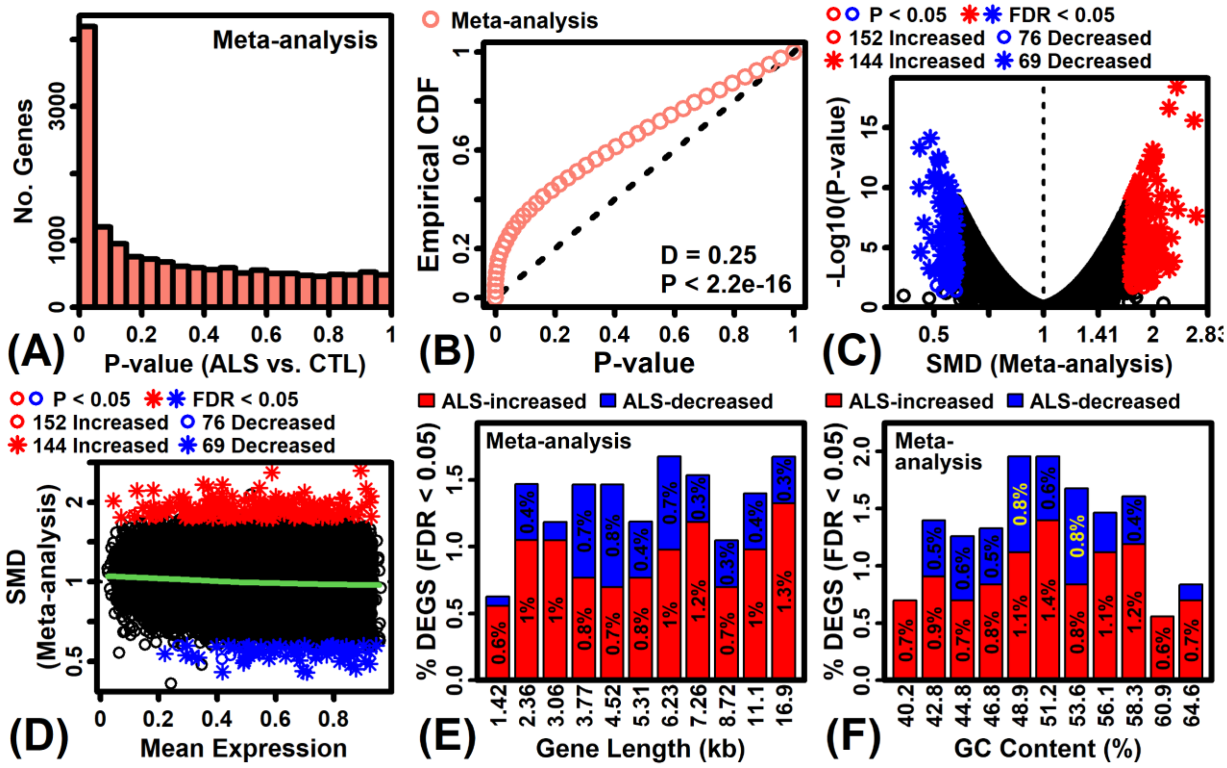

Figure S18. Differential expression meta-analyses (ALS vs. CTL).

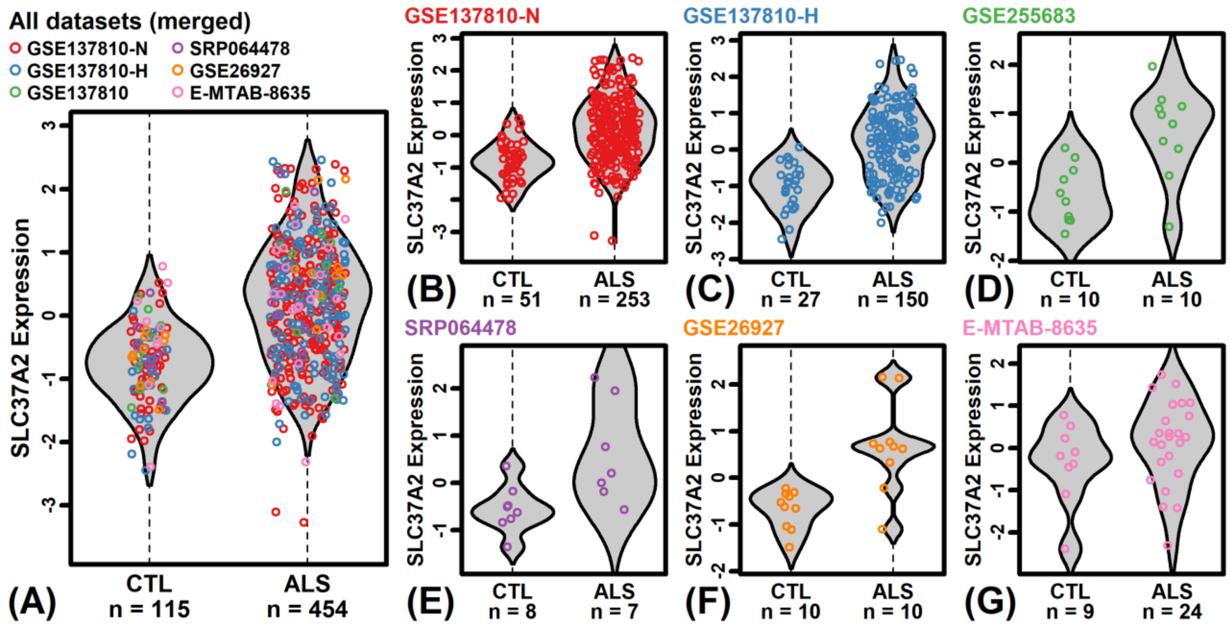

Figure S19. ***SLC37A2*** **expression summary.**

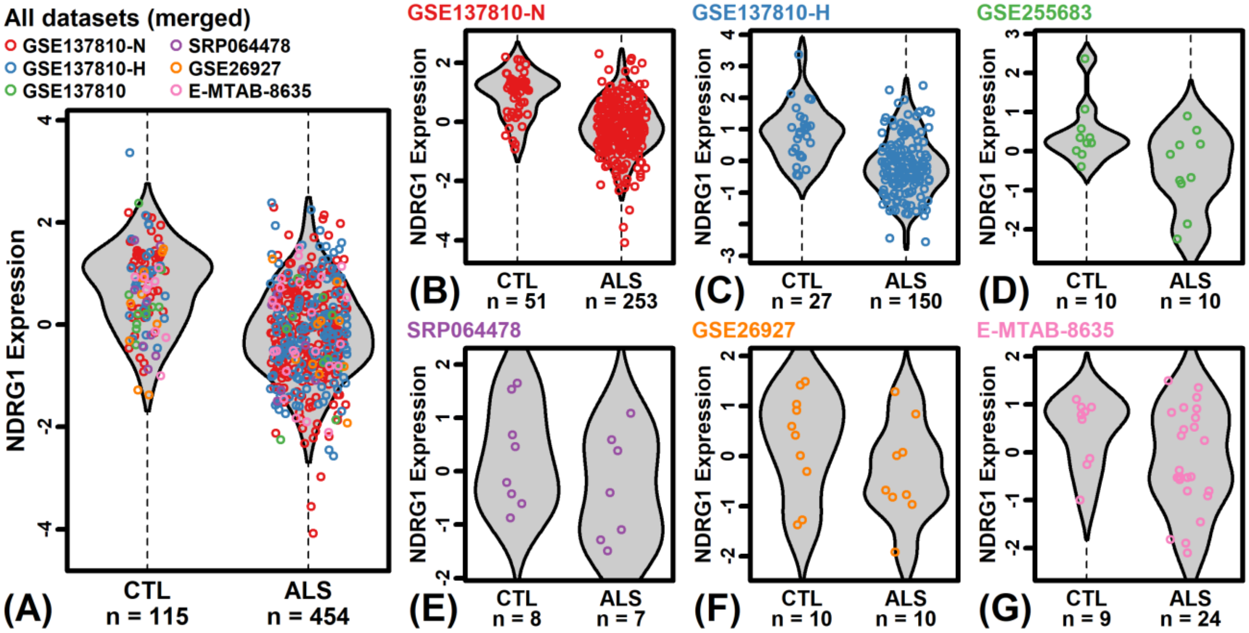

Figure S20. ***NDRG1*** **expression summary.**

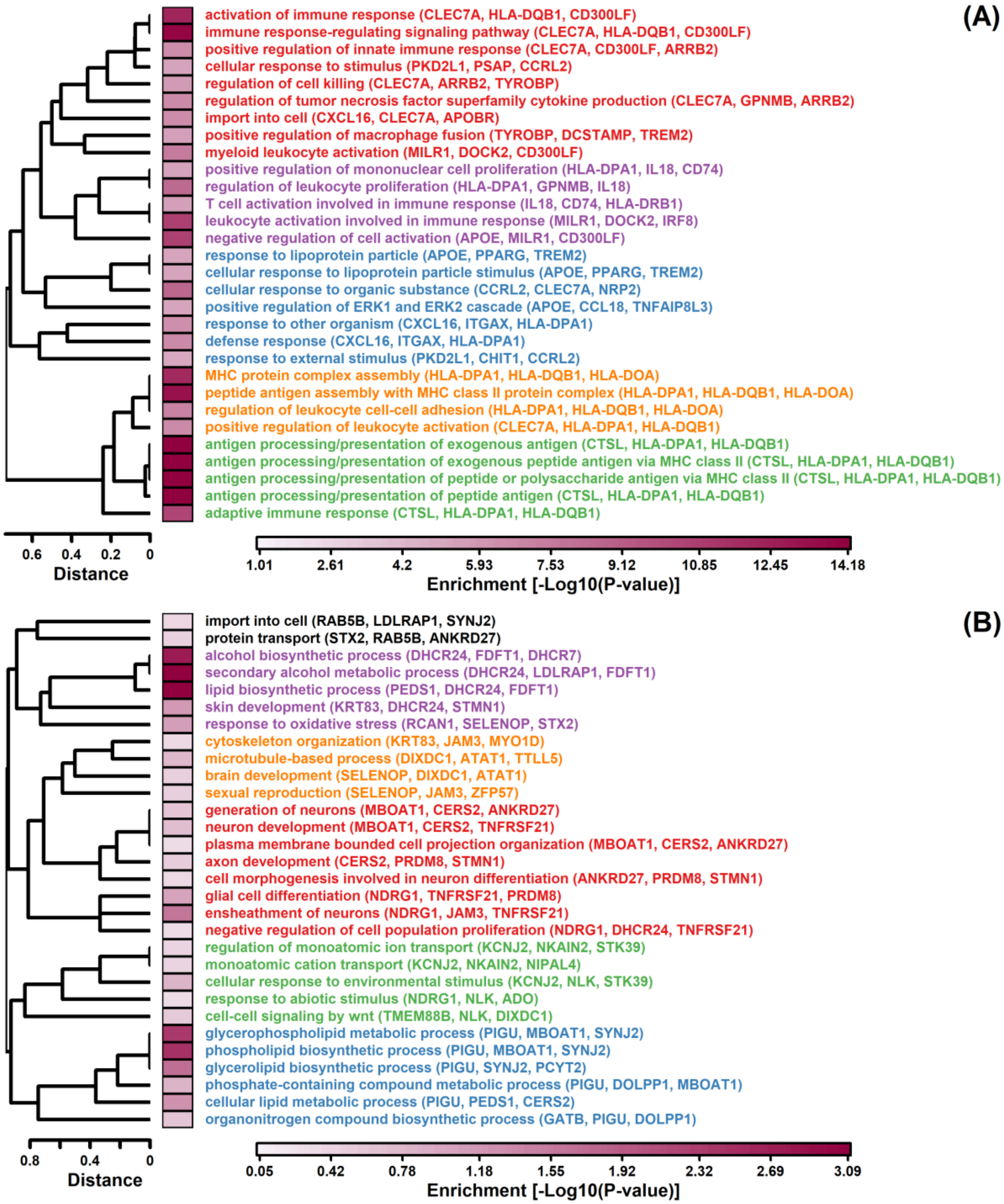

Figure S21. GO BP term cluster analysis.

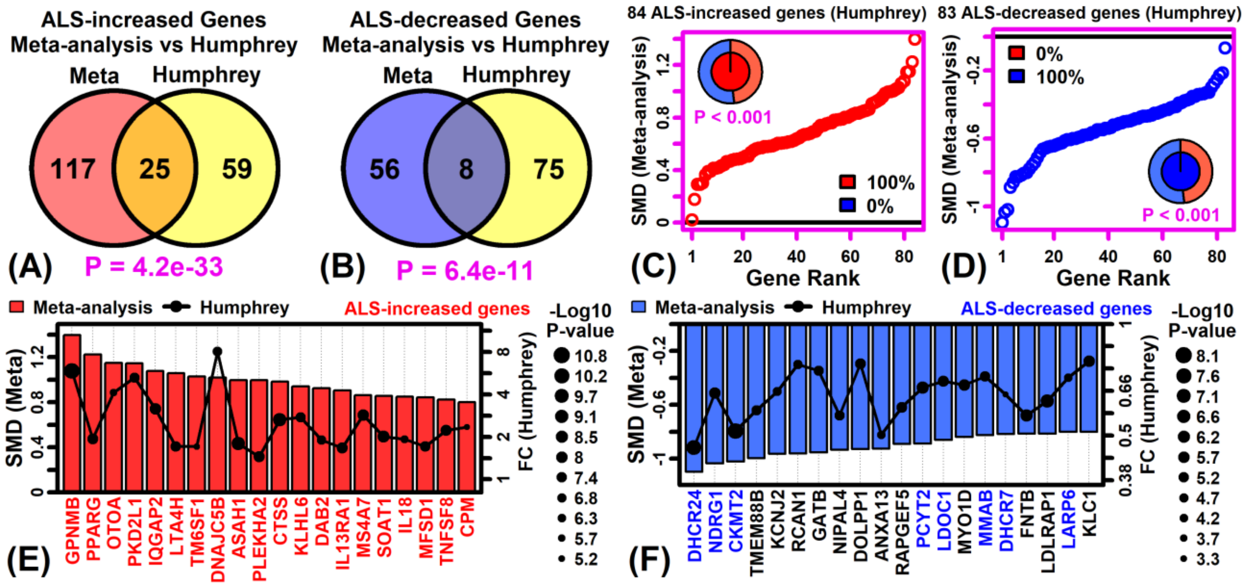

Figure S22. Comparison of meta-analysis DEGs to those previously identified from NYGC ALS Consortium data (Humphrey et al. 2023, Nat Neurosci 26:150-162).

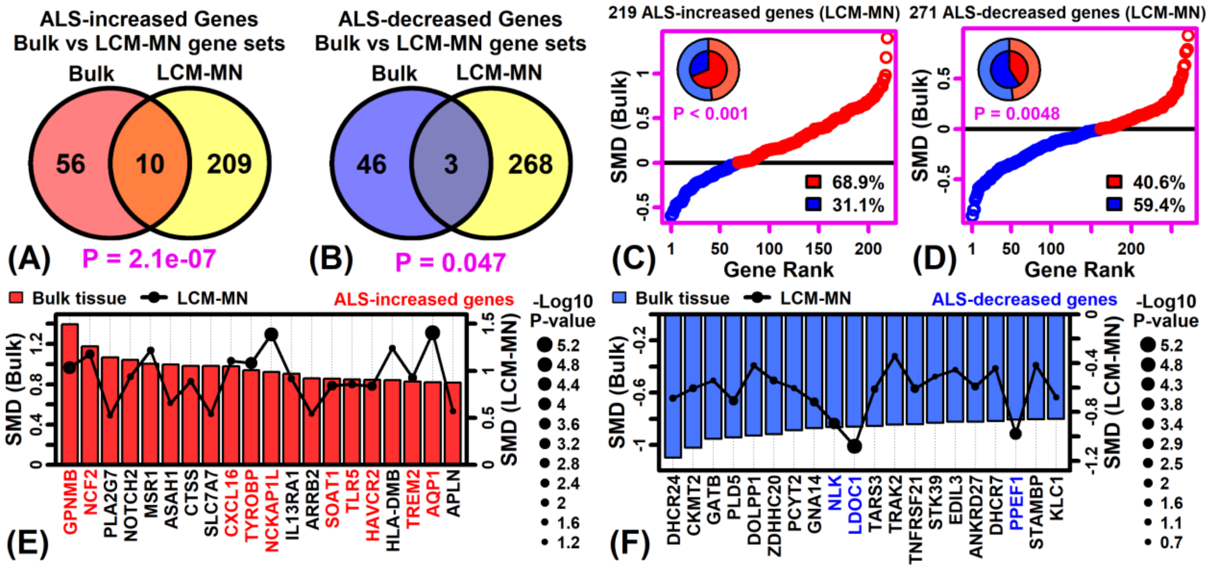

Figure S23. Meta-analysis comparison of ALS gene dysregulation in human spinal cord (bulk tissue vs. LCM-MN).

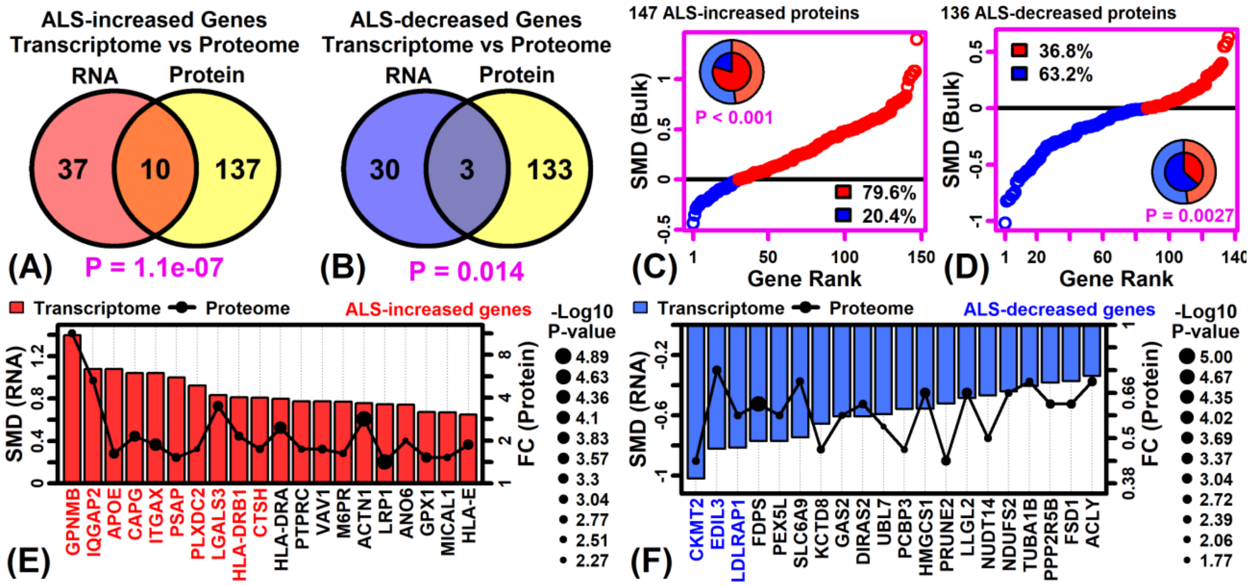

Figure S24. Overlap between DEGs and proteins dysregulated in ALS spinal cord.

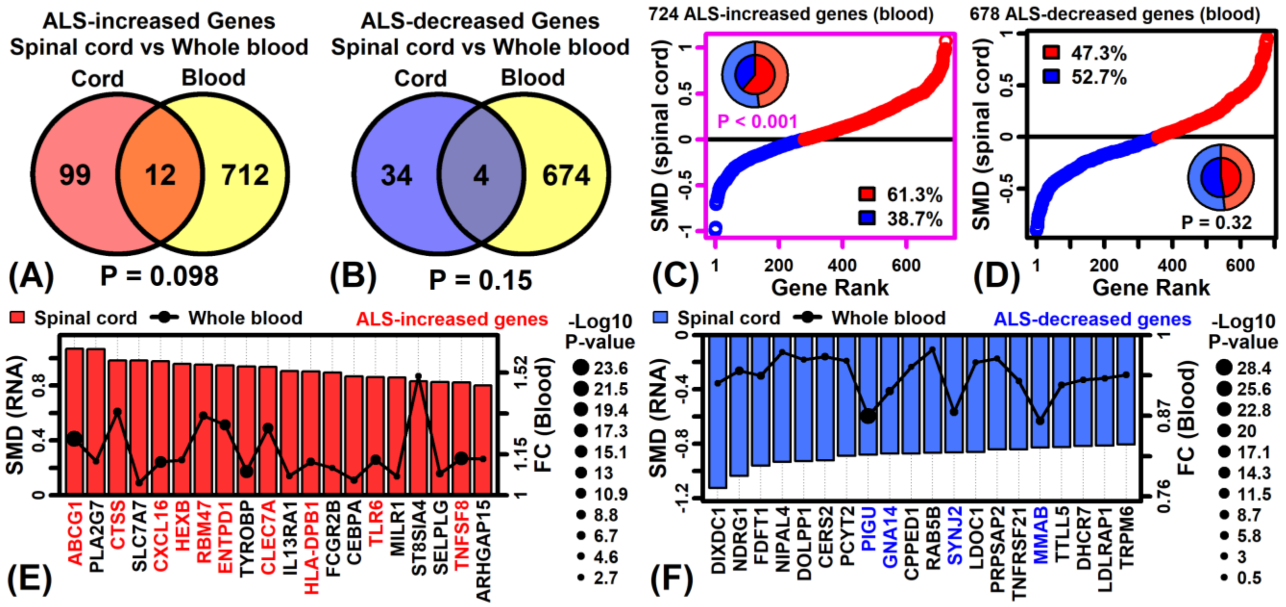

Figure S25. Overlap between DEGs and genes dysregulated in whole blood from ALS patients.

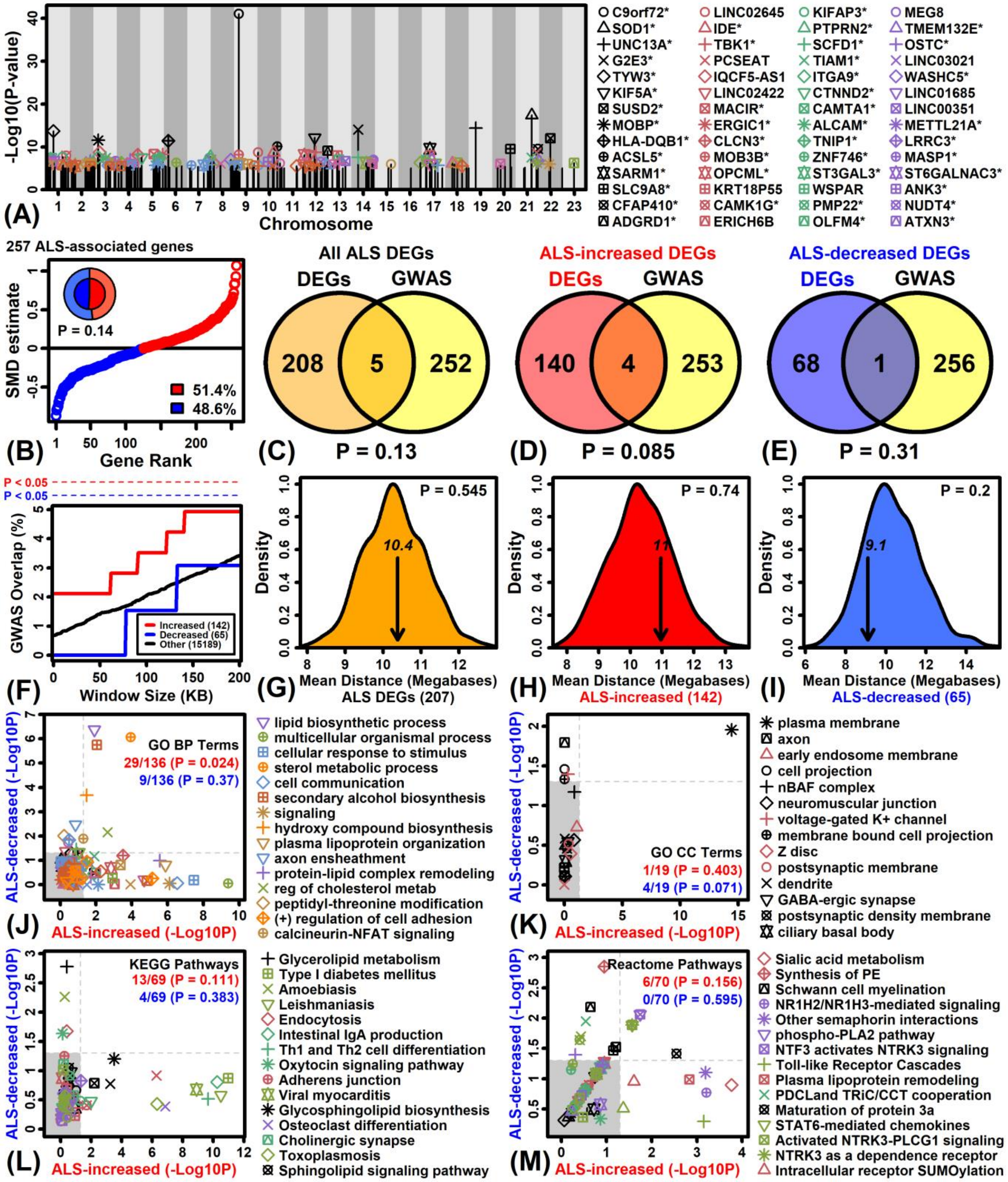

Figure S26. Overlap between DEGs and genes near ALS GWAS loci.

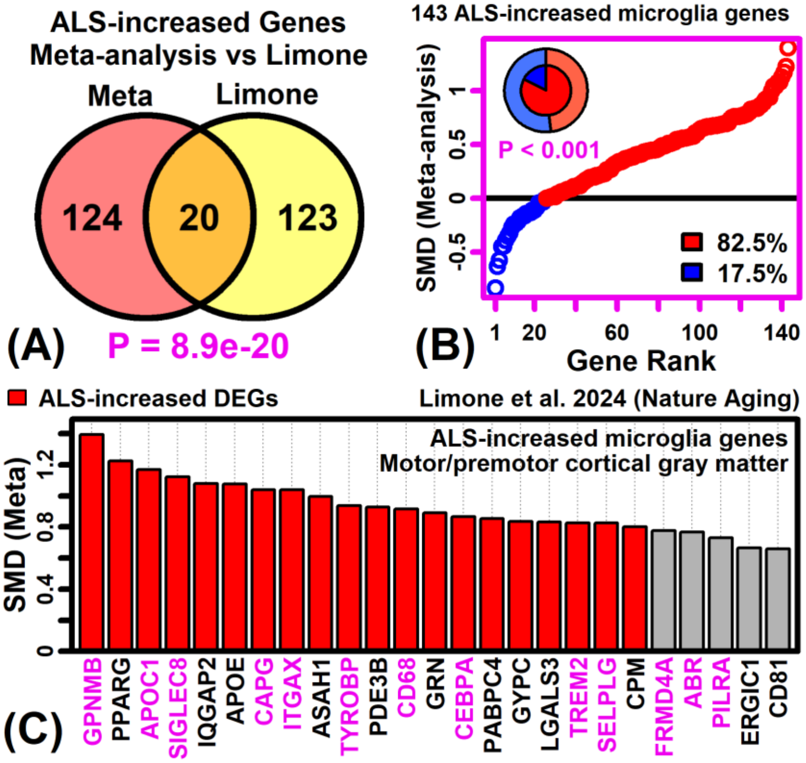

Figure S27. ALS-increased DEG comparison to ALS-increased microglia genes from motor/premotor cortical gray matter (snRNA-seq study by Limone et al. 2024, Nature Aging 4:984-997).

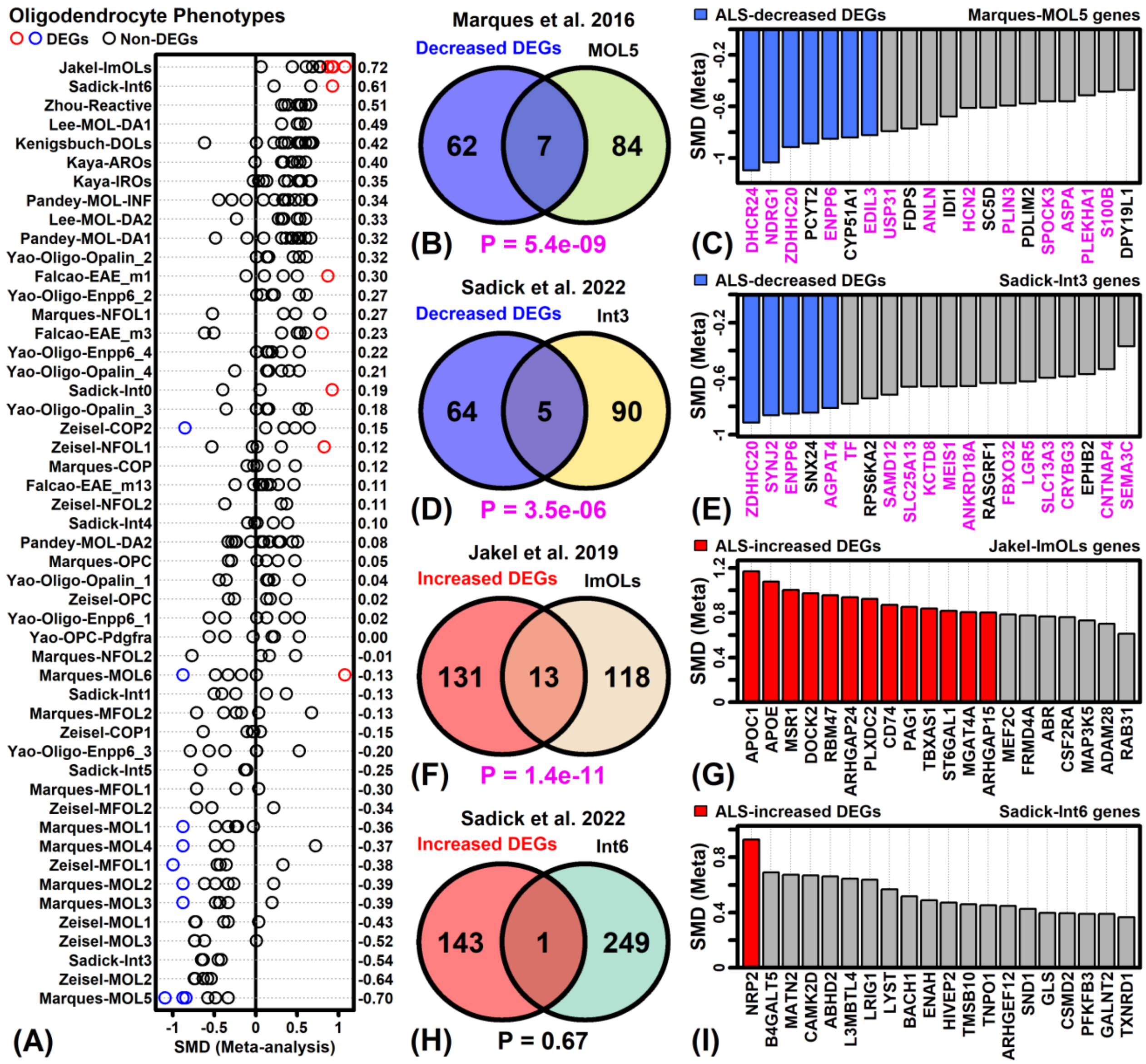

Figure S28. Oligodendrocyte (OD) phenotypes.

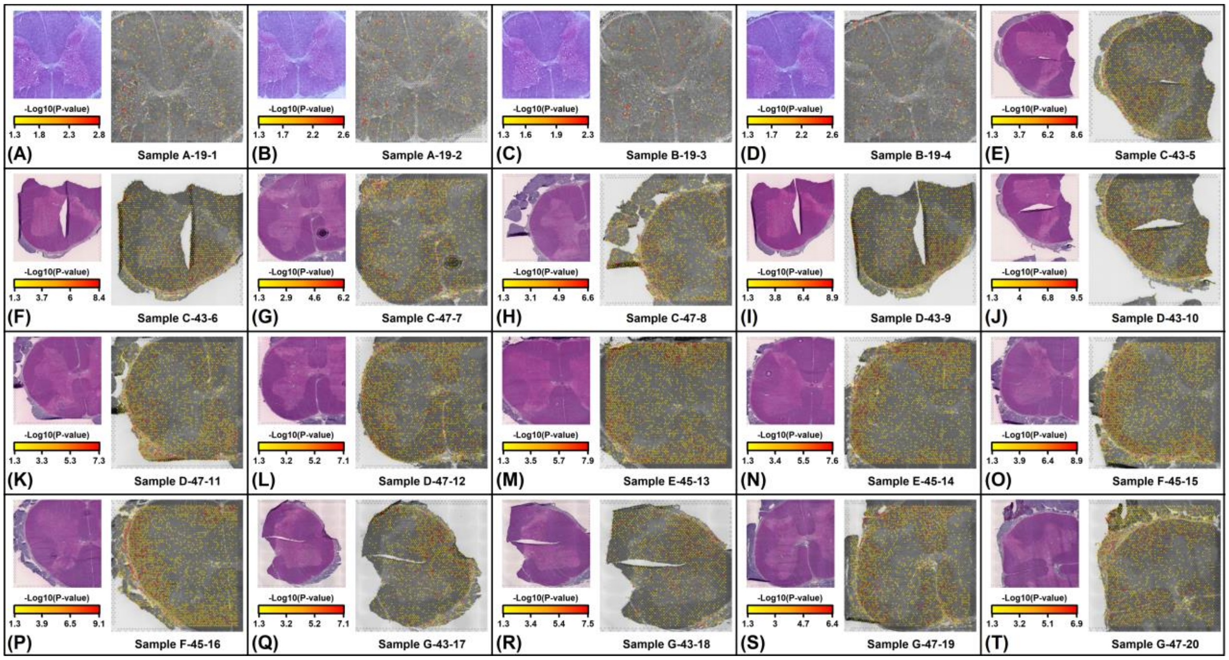

Figure S29. Regional enrichment of ALS-increased DEG expression in normal human spinal cord (GSE222322, 10x Genomics Visium array).

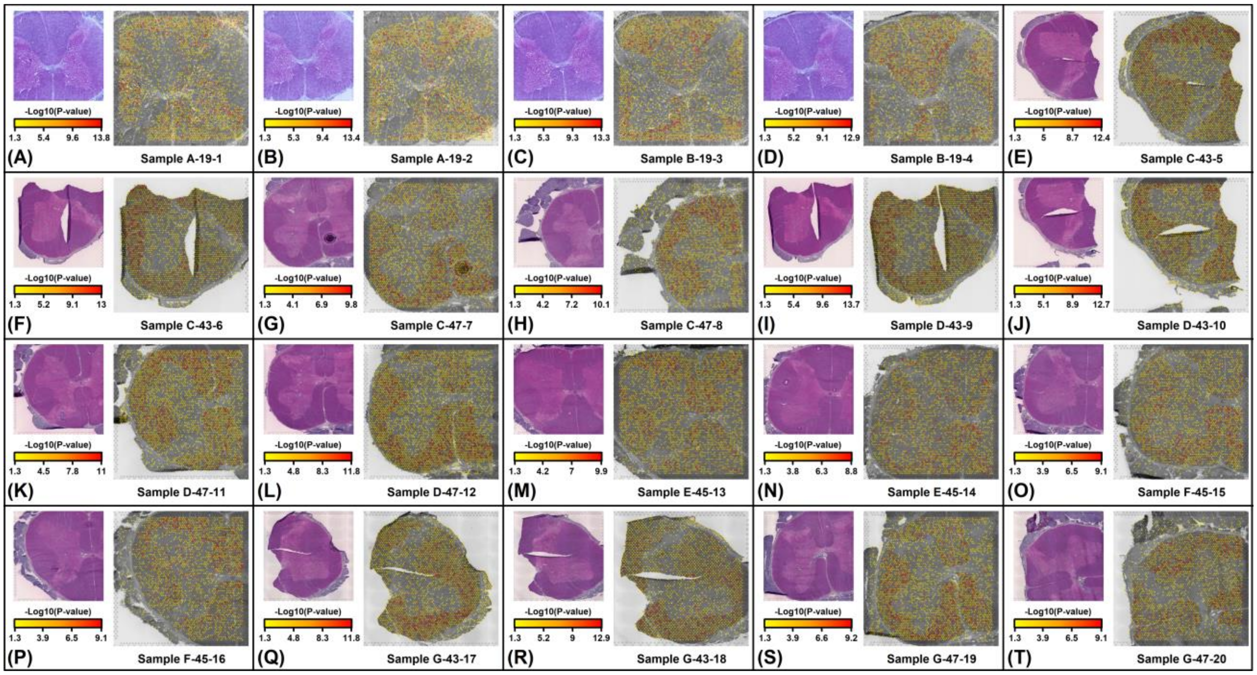

Figure S30. Regional enrichment of ALS-decreased DEG expression in normal human spinal cord (GSE222322, 10x Genomics Visium array).

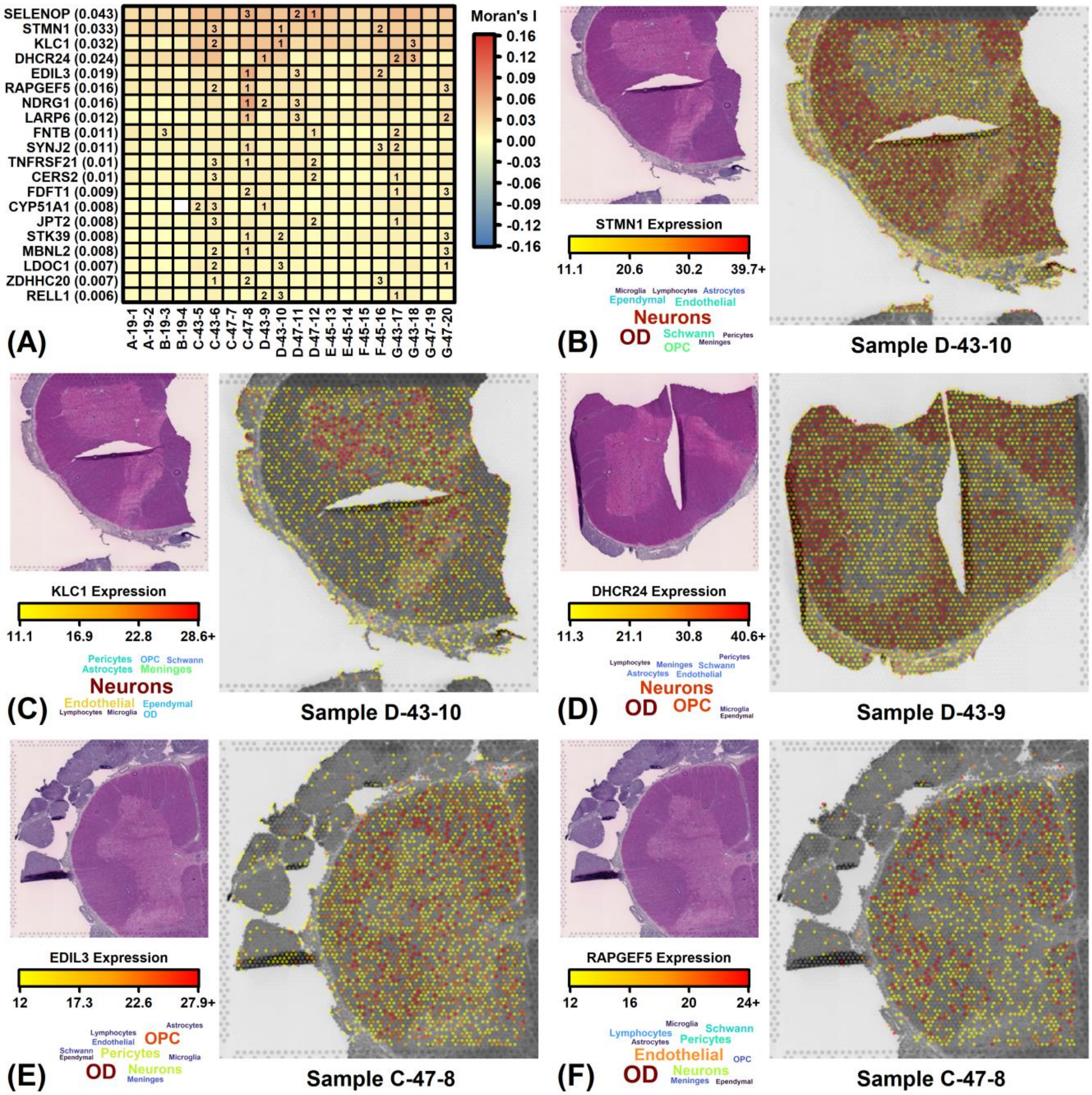

Figure S31. ALS-decreased DEGs with high spatial heterogeneity in normal human spinal cord (GSE222322).

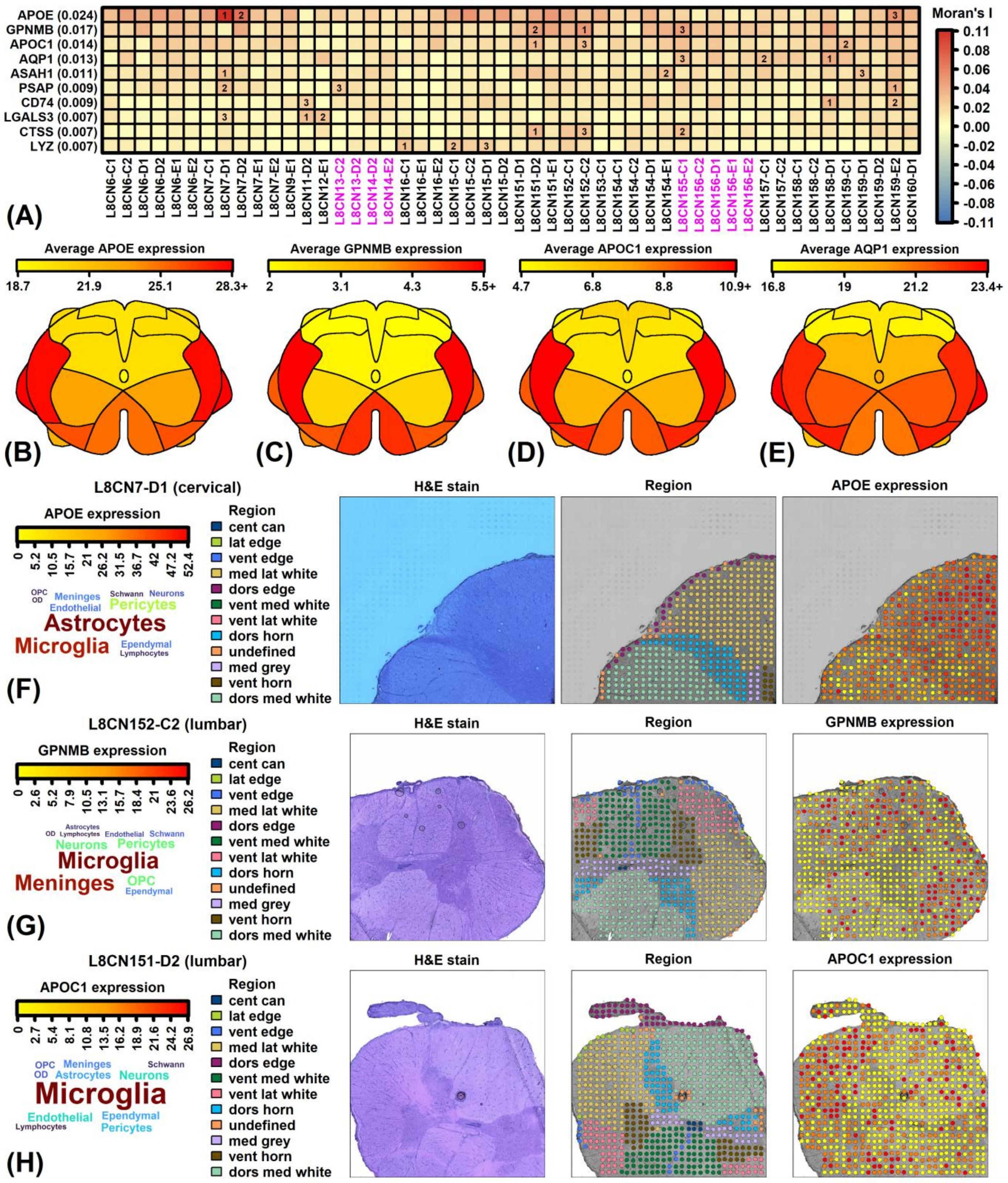

Figure S32. ALS-increased DEGs with high spatial heterogeneity in ALS patient spinal cord sections (Maniatis et al. 2019, Science 364: 89-93).

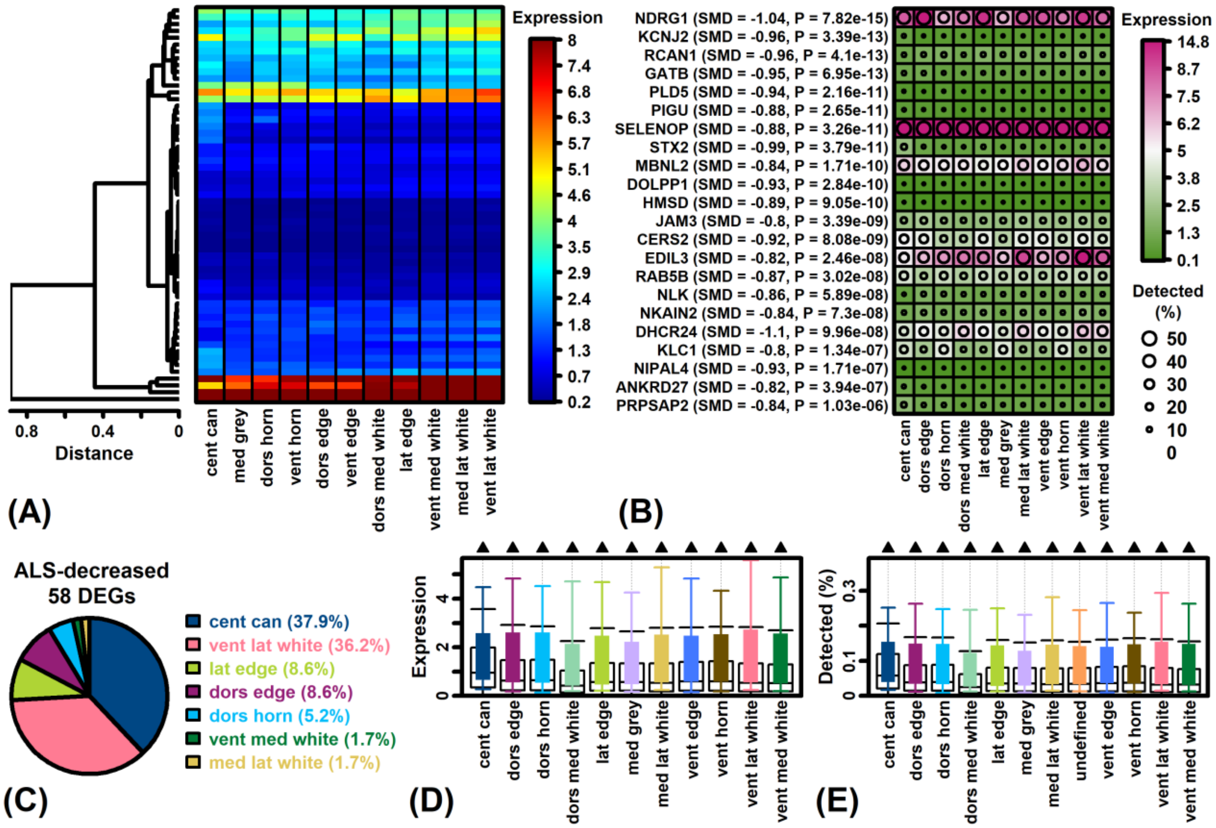

Figure S33. ALS-decreased DEGs and their regional expression in ALS patient cervical/lumbar cord segments (Maniatis et al. 2019, Science 364: 89-93).

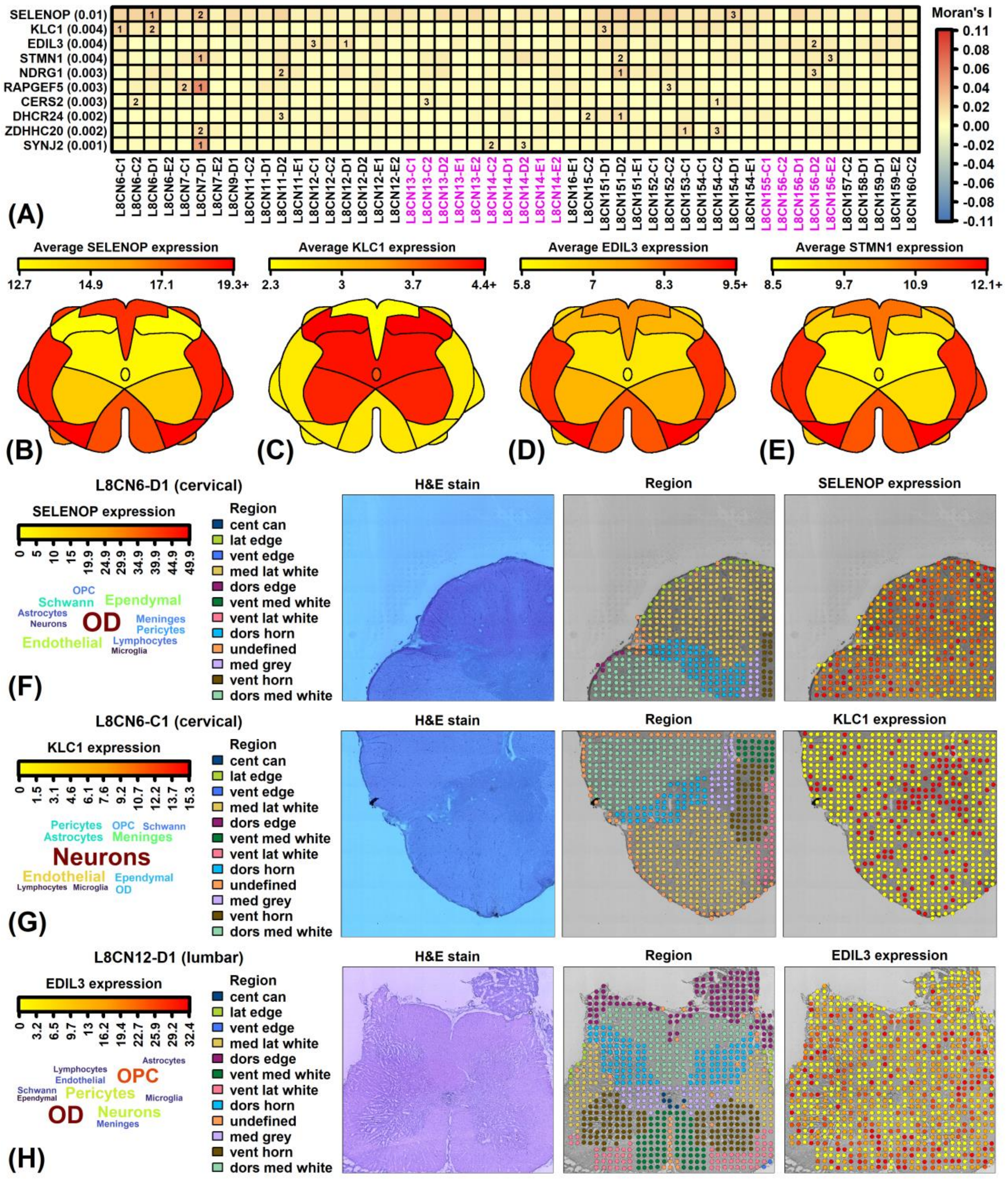

Figure S34. ALS-decreased DEGs with high spatial heterogeneity in ALS patient spinal cord sections (Maniatis et al. 2019, Science 364: 89-93).
